# Scaffold-Hopping Strategy on a Series of Proteasome Inhibitors Led to a Preclinical Candidate for the Treatment of Visceral Leishmaniasis

**DOI:** 10.1021/acs.jmedchem.1c00047

**Published:** 2021-04-27

**Authors:** Michael Thomas, Stephen Brand, Manu De Rycker, Fabio Zuccotto, Iva Lukac, Peter G. Dodd, Eun-Jung Ko, Sujatha Manthri, Kate McGonagle, Maria Osuna-Cabello, Jennifer Riley, Caterina Pont, Frederick Simeons, Laste Stojanovski, John Thomas, Stephen Thompson, Elisabet Viayna, Jose M. Fiandor, Julio Martin, Paul G. Wyatt, Timothy J. Miles, Kevin D. Read, Maria Marco, Ian H. Gilbert

**Affiliations:** † Drug Discovery Unit, Wellcome Centre for Anti-Infectives Research, Division of Biological Chemistry, University of Dundee, Dundee DD1 5EH, U.K.; ‡ Global Health R&D, GlaxoSmithKline, Tres Cantos 28760, Spain

## Abstract

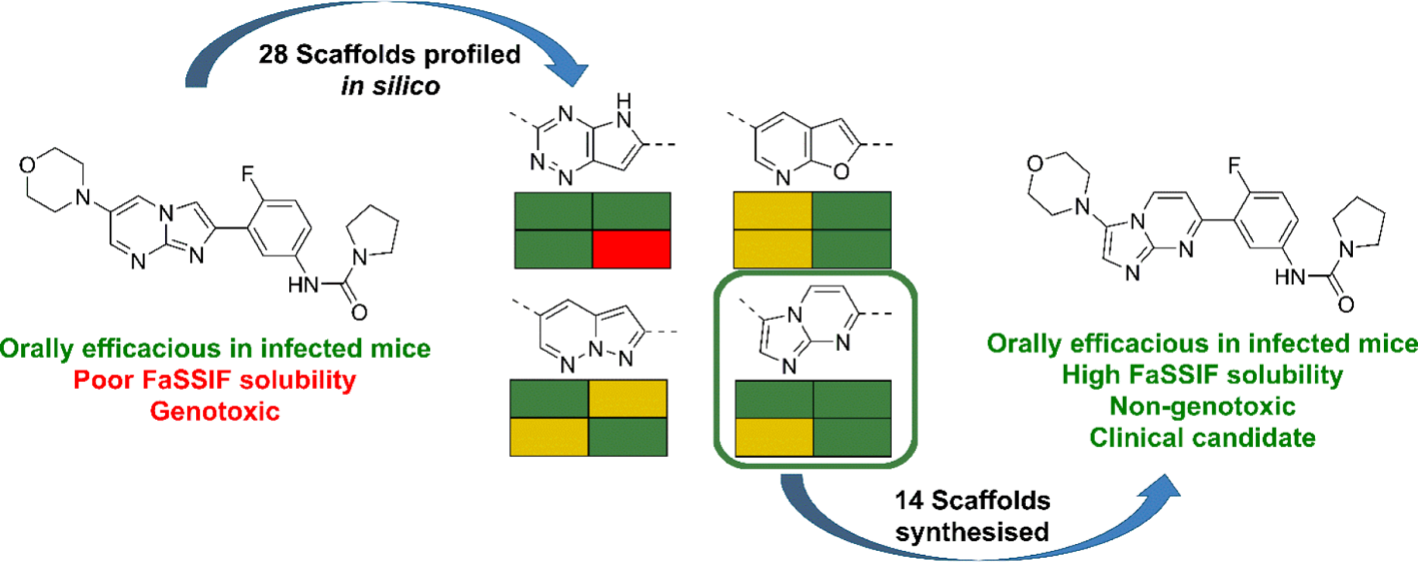

There is an urgent need for new treatments for visceral leishmaniasis (VL), a parasitic infection which impacts heavily large areas of East Africa, Asia, and South America. We previously reported on the discovery of GSK3494245/DDD01305143 (**1**) as a preclinical candidate for VL and, herein, we report on the medicinal chemistry program that led to its identification. A hit from a phenotypic screen was optimized to give a compound with *in vivo* efficacy, which was hampered by poor solubility and genotoxicity. The work on the original scaffold failed to lead to developable compounds, so an extensive scaffold-hopping exercise involving medicinal chemistry design, *in silico* profiling, and subsequent synthesis was utilized, leading to the preclinical candidate. The compound was shown to act via proteasome inhibition, and we report on the modeling of different scaffolds into a cryo-EM structure and the impact this has on our understanding of the series’ structure–activity relationships.

## Introduction

Bites from infected sandflies are known to transmit *Leishmania* parasites, a protozoan parasite. There are more than 20 different species of *Leishmania,* which cause a group of diseases that manifest in three main clinical forms: cutaneous, mucocutaneous, and visceral leishmaniasis (VL, also referred to as kala-azar).^1−3^ There is also the complication of VL, called post kala-azar dermal leishmaniasis. While the other three forms cause major health problems such as nonhealing ulcers and scarring, VL is the most severe and becomes lethal if not treated. Recurrent epidemics of VL in East Africa (Ethiopia, Kenya, South Sudan, and Sudan) have caused high morbidity and mortality in those countries and together with Brazil, India, and Somalia accounted for more than 90% of the new cases reported in 2018.^4^ VL is usually due to infection with either *Leishmania donovani* or *Leishmania infantum*. The infection causes irregular bouts of fever, substantial weight loss, swelling of the spleen and liver, and anemia. Current available treatments are not adequate due to high toxicity, resistance issues, treatment cost and duration, or the need for hospitalization to administer the treatment, a particularly relevant aspect given the settings where the disease is endemic. Indeed, the only effective oral treatment for VL is miltefosine, although it requires 28 days of dosing, and contraception is necessary for women both during and after treatment due to its teratogenicity. For reasons that are poorly understood, clinical outcomes for these drugs seem to vary from region to region of the world, with much reduced cure rates being observed in East Africa and South America. Given these factors, the need for safer, oral, short-course, and low-cost medicines for the treatment of VL is urgent.

**Figure 1:**
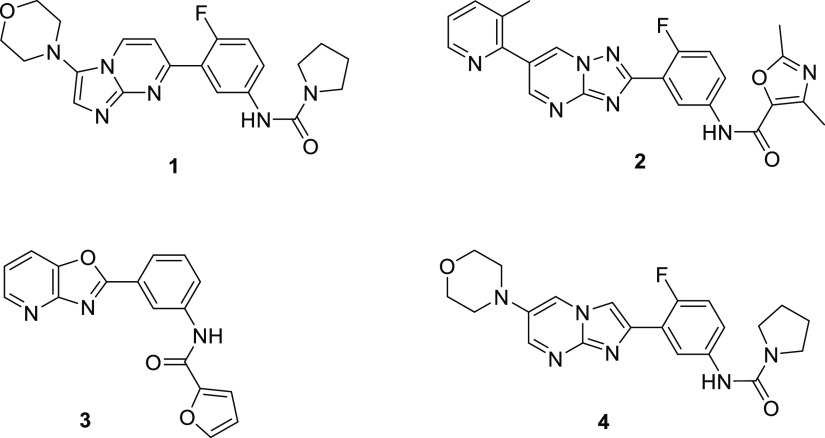
Compounds **1** (DDD01305143/GSK3494245), **2** (LXE408), **3** (initial hit), and **4** (early lead).

There is some cause for optimism, as for the first time, there are a number of compounds in late preclinical or early clinical development, as indicated on the Drugs for Neglected Diseases *Initiative* (DND*i*) website.^5^ We recently reported a leishmanial CRK-12 inhibitor as a preclinical candidate;^6,7^ Novartis (LXE408, compound **2**)^8,9^ and ourselves (GSK3494245/DDD01305143, compound **1**)^10^ have both reported proteasome inhibitors (Figure 1); DND*i* have other compounds in clinical development, notably an oxaborole and a nitroimidazole, although the mechanism of action (MoA) of these has not been reported.^5^ In all these cases, the optimization was run phenotypically, with the MoA only being elucidated during or after the medicinal chemistry program. It must be noted^10^ that there is a lack of validated drug targets for *Leishmania*.

As we reported recently, compound **1** was the product of a program which started from substituted 2-phenyloxazolo[4,5-*b*]pyridine **3** which was identified through phenotypic screening against the related pathogen *Trypanosoma cruzi*, the causative agent of Chagas’ disease (Figure 1). Subsequently, compound **3** was screened against the physiologically relevant intramacrophage form of *L. donovani,* where it showed weak activity. In parallel to our efforts, *N*-(4-chloro-3-(oxazolo[4,5-*b*]pyridin-2-yl)phenyl)furan-2-carboxamide (the 4-chloro phenyl analogue of **3**) had also been identified by the groups of Buckner, Gelb, Baell, and Guy as a compound with antikinetoplastid activity, with their optimization being subsequently reported.^8,11−15^

As previously described, an initial scaffold hop from **3** led to a 2-phenylimidazo[1,2-*a*]pyrimidine and optimization of the 6-position of the bi-cycle led to a 6-morpholino substituent which increased the potency and metabolic stability. Alongside this, the fluorination of the phenyl ring increased potency and replacement of the furanyl amide with a pyrrolidinyl urea improved solubility (similar changes had also been observed on related scaffolds by other groups^13,15^). This led to compound **4**, which showed efficacy in a mouse model of VL.^10^ The key to the success of this compound series was a further set of scaffold hops from 2-phenylimidazo[1,2-*a*]pyrimidine, and this strategy, along with the discussion of the structure–activity relationships (SARs) around the 2-amino group, is described in detail herein. Although the series was developed phenotypically, it was subsequently found to act principally through the inhibition of the β5-subunit of the proteasome, specifically at the “chymotrypsin” site, and the relationship between the antiparasitic SAR and the identified target is also discussed in detail.^10^

## Results

Compounds were initially screened against *L. donovani* in an intracellular assay (*L. donovani* cultured in differentiated THP1 cells, referred to as the “INMAC assay”^16^), where we targeted pEC_50_ values >5.8, alongside stability in mouse liver microsomes of <5.0 mL/min/g (Cl_i_) and kinetic aqueous solubility >100 μM (measured by chemiluminescent nitrogen detection, CLND). We had successfully used these criteria in the hit to lead stage to identify compounds for *in vivo* proof-of-concept studies in another program, which subsequently led to the identification of preclinical candidate DDD853651/GSK3186899 active against CRK12.^7^ As reported previously^10^ and shown in Table 1, *in vitro* profiling of **4** shows that it met these targets, with an INMAC pEC_50_ value of 6.2, Cl_i_ of <0.5 mL/min/g in mouse liver microsomes, and aqueous solubility of 150 μM, alongside high mouse and human plasma stability (100% compound remaining after 3 h). A pharmacokinetic (PK) study was thus performed, dosed at 10 mg/kg po and 3 mg/kg iv. An oral dose of 10 mg/kg showed **4** to have blood levels above EC_90_ for around 2 h (pEC_90_ 5.7, 780 ng/mL), which would translate to coverage above 6 h for a 50 mg/kg dose, assuming a linear dose increase (see Supporting Information). With the limited knowledge of the PK/PD drivers for the treatment of VL, we considered this to be a suitable profile for the progression of **4** into a previously reported mouse efficacy model of VL,^6,7,17,18^ where it was dosed twice daily at 50 mg/kg ip in order to further maximize exposure and give the best chance of achieving proof-of-concept.

**Table 1:** In Vitro and In Vivo Profile of Compound **4**

*in vitro* profile	
INMAC pEC_50_^a^^,^^b^	6.2
THP-1 cells pEC_50_^a^^,^^b^	<4.3
aqueous solubility (μM)^c^	150
FaSSIF solubility (μM)^a^	15
CL_i_ mL/min/g^a^^,^^d^	<0.5

i*n vivo* profile^e^		
iv PK (3 mg/kg)	*T*_1/2_ (h)	0.5
	AUC ng·min/mL	135,000
	Clb mL/min/Kg	22
po PK (10 mg/kg)	*C*_max_ (ng/mL)	1300
	*T*_max_ (h)	0.5
	AUC ng·min/mL	258,000
	Vdss L/kg	0.8
	*F* %	57
% reduction in parasitemia^f^		98

a Data reported previously.^10^

b INMAC is the intramacrophage assay carried out in THP-1 cells with *L. donovani* amastigotes. Data are the result of five independent replicates and standard deviations are ≤0.2.

c Aqueous solubility is kinetic solubility measured by CLND.

d Cl_i_ is the mouse liver microsomal intrinsic clearance.

e Mouse PK studies dosed at 10 mg/kg po and 3 mg/kg iv.

f Infected mouse model of VL, dosed at 50 mg/kg ip, b.i.d for 5 days.

After dosing twice daily for 5 days, a reduction in parasitemia of 98% was observed (Table 1). This established the proof-of-concept for this series in VL and, in fact, met our criteria for a preclinical development candidate.^7^ Unfortunately, despite reasonable kinetic aqueous solubility, **4** was poorly soluble in FaSSIF (Fasted State Simulated Intestinal Fluid)^19,20^ media and this, alongside a positive result in the Ames assay,^21−23^ as reported previously,^10^ which indicated potential genotoxicity issues, precluded further development of this particular compound.

Having achieved the *in vivo* proof-of-concept, the series moved into the lead-optimization phase, with an initial aim to identify a compound with a suitable profile for rodent toxicology studies. As this would require us to achieve an exposure significantly higher than the minimum efficacious dose, we continued to target compounds with pEC_50_ values >6 and now aimed for FaSSIF solubilities >100 μM and to maintain Cl_i_ < 1 mg/mL/g. We carried out a systematic exploration of the molecule, initially preparing further analogues of **4** with variations to the pendant substituents of the 2-phenylimidazo[1,2-*a*]pyrimidine core. However, this failed to identify any better molecules (data not shown).

We next focused our attention on the core phenyl-substituted bi-cycle. Initially, we investigated changes to the phenyl ring and, as shown in Table 2, modifications to this part of the scaffold led to analogues that maintained stability in mouse liver microsomes and also showed improved solubility in both aqueous (CLND) and FaSSIF media. Unfortunately, these changes, such as moving or removing fluorine (**5** and **6**, respectively), introducing alternative substituents (e.g., OMe analogues **7** and **8**), or replacing phenyl with six-membered heterocycles (e.g., pyridine **9**, pyrimidines **10** and **12**, pyrazine **11**, and pyridazine **13**), all led to a >10-fold loss of potency. We noted that similar findings were also reported by Tatipaka et al.^13^ and Ferrins et al.^14^ on the related scaffolds. Analogue **4**, with the fluorine ortho to the bi-cyclic substituent, appeared to be optimal and our subsequent knowledge of the binding mode of these compounds allowed us to rationalize this (seen later). We were interested to note that the changes made did have a big impact on FaSSIF solubility, with methoxy analogue **8**, pyrazine **11**, pyridazine **13**, and pyrimidines **10** and **12** all having FaSSIF solubilities well above 100 μM. Also, **10–13**, all had intrinsic clearances of <0.5 mL/min/g. Encouraged by this, we turned our attention to the core bi-cycle, surmising that changes to the pattern of hydrogen bond acceptors (HBAs) could lead to similar improvements in solubility.

**Table 2:**
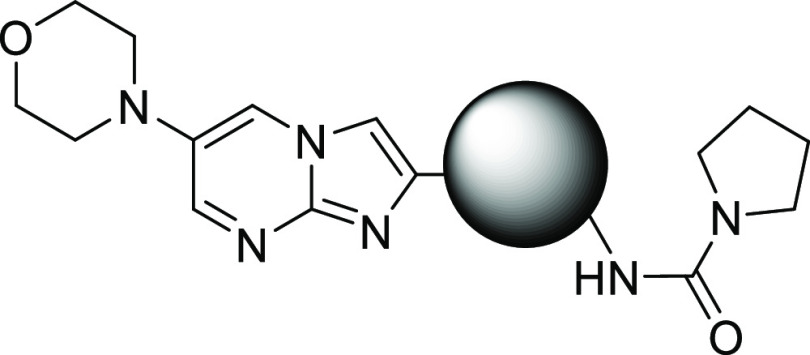

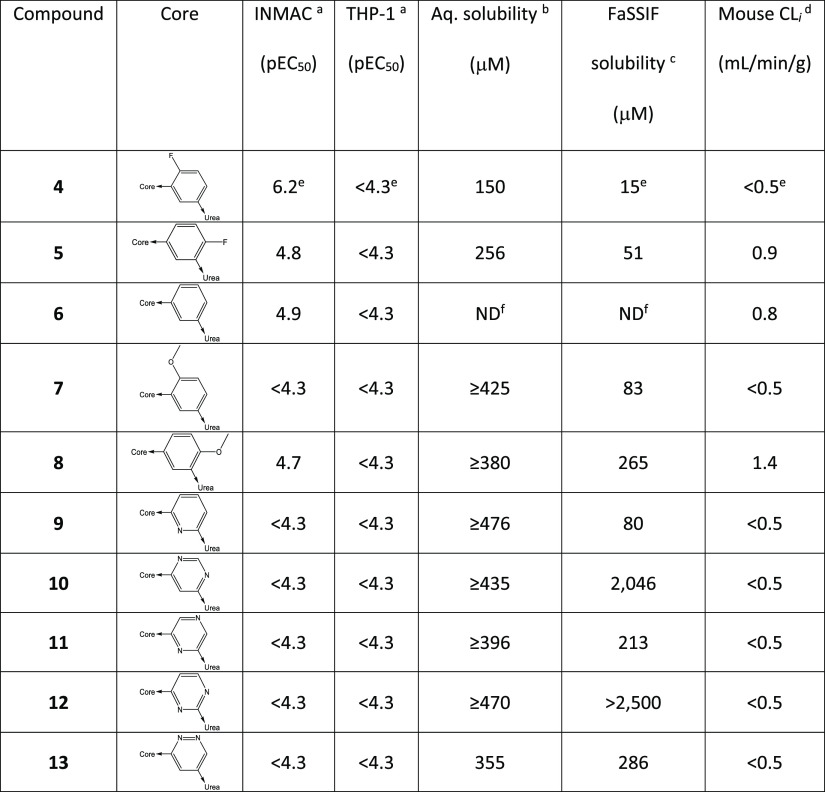
Profile of Analogues **4–13**, with Substitutions and Nitrogen Insertions on the Central Phenyl Ring

a INMAC is the intramacrophage assay carried out in THP-1 cells with *L. donovani* amastigotes. Data are the result of at least three independent replicates and standard deviations are ≤0.4.

b Aqueous solubility is the kinetic solubility measured by CLND.

c FaSSIF solubility is the fasted state simulated intestinal fluid solubility.

d Cl_i_ is the mouse liver microsomal intrinsic clearance.

e Data reported previously.^10^

f ND means not determined.

In order to define a set of alternative bi-cycles for synthesis, we first looked at the SAR from our early hit discovery efforts, on scaffolds with no 6-substituent (Table 3). On comparing with initial hit **3**, we noted that a scaffold which removed the HBA in the 8-position (e.g., **14**) showed no activity in the INMAC assay. We therefore focused our scaffold-hopping efforts on bi-cycles, which contained a HBA in the 8-position, and then prioritized them based on cChromLogD (ChromLogD^24^ is the chromatographically determined log *D* and cChromLogD is the calculated version of this), cp*K*_a_ (calculated using ACD p*K*_a_ predictor), calculated aqueous solubility (using Gastroplus v9.7), and synthetic tractability (based on literature precedent and in-house knowledge). Without a robust model for activity in the Ames test, this was not included in the design process, the intention being to profile compounds in the assay when they demonstrated suitable efficacy in the VL mouse model to warrant further study.

**Table 3:**
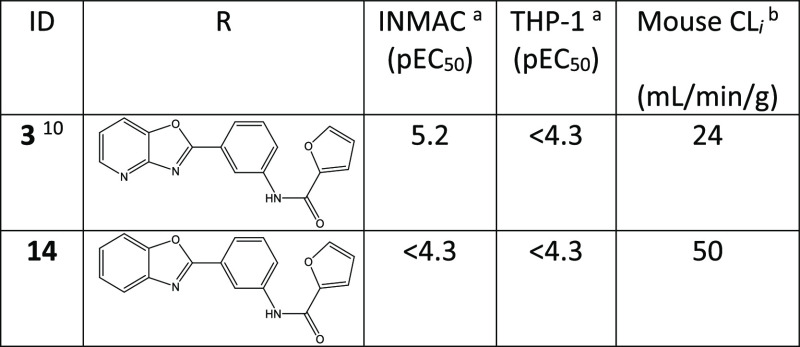
Initial Modifications of the Bi-cyclic Core

a INMAC is the intramacrophage assay carried out in THP-1 cells with *L. donovani* amastigotes. Data are the result of at least three independent replicates and standard deviations are ≤0.4.

b Cl_i_ is the mouse liver microsomal intrinsic clearance.

**Figure 2:**
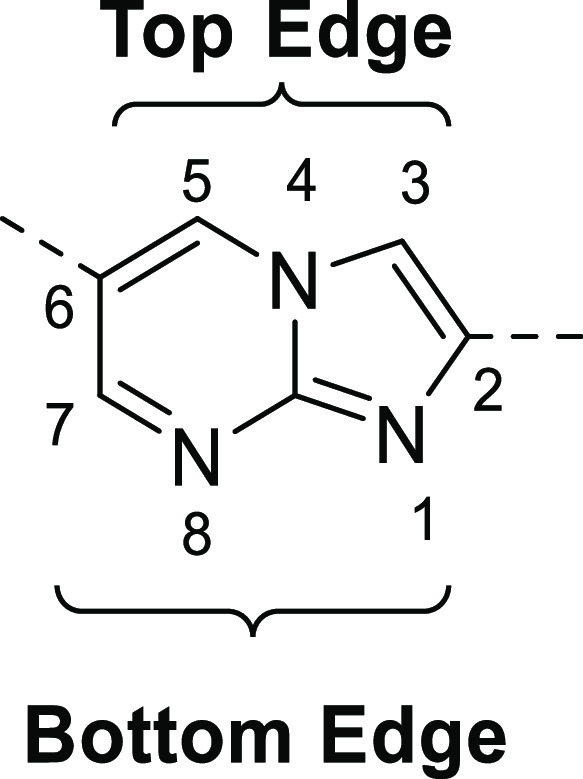
Numbering for the imidazo[1,2-*a*]pyrimidine scaffold.

A total of 28 alternative scaffolds were evaluated *in silico*, all maintaining a HBA in the 8-position (for comparison purposes, the numbering for the imidazo[1,2-*a*]pyrimidine scaffold in Figure 2 is used in this section), plus the correct orientation of the morpholinyl and phenyl substituents. We explored HBDs and HBAs in various positions around the bi-cycle and also examined the effect of reversing the 6,5-fused system so that morpholine was attached to the five-membered ring and phenyl to the six-membered ring. Finally, we also profiled a fused 5,5 bi-cycle.

As we reported for a previous series, we had focused on reducing lipophilicity as a key strategy for improving solubility.^7^ In that case, we targeted a PFI^25^ <7 (PFI = property forecast index, ChromLogD + #Ar). Within the current series which contained three aromatic rings, this would equate to a cChromLogD below 4, so we utilized this as a key property for the designed scaffolds. We also targeted scaffolds which had a higher predicted solubility than **4** and scaffolds which increased cp*K*_a_. The increased cp*K*_a_ would indicate potential for improved solubility, as well as allowing possible salt formation. We then further prioritized based on the potential synthetic tractability (based on literature precedent and in-house synthetic expertise), as with each scaffold requiring a bespoke synthesis, there would be a limited chemistry resource to develop completely novel routes.

**Figure 3:**
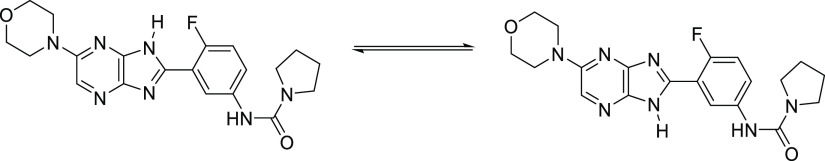
Tautomerization of scaffold **S-11**.

The initial scaffolds all examined the nature of the atom in the 1-position of the bi-cycle (**S-1** to **S-16**) (Table 4). Scaffolds **S-1–S-8** all examined the removal of the HBA, where the best predicted profiles were obtained for **S-1**, **S-4**, **S-5**, and **S-6**, all predicted to have cChromLogD below 4 and either higher cp*K*_a_, improved solubility, or both when compared to **3** (Table 4). Scaffolds **S-9** and **S-10** would demonstrate whether a hydrogen bond donor (HBD) was acceptable in position 1. Although **S-9** gave a more direct comparison to **3**, **S-10** proved to be more synthetically tractable. On this basis, **S-1** and **S-10** were synthesized with the desired substituents, **S-5** was synthesized as the non-fluorinated analogue, and **S-9** as the phenyl analogue. Although **S-4**, **S-6**, and **S-11** had good *in silico* profiles, the syntheses of **S-4** and **S-6** were not completed when it became apparent that the removal of HBA at the 1-position would lead to an inactive compound, and for **S-11**, there would be a possible tautomerization of the five-membered ring, making any data generated difficult to interpret so this scaffold was not synthesized (see Figure 3). **S-12** would further probe the nature of the heteroatom in the 1-position and had a very good *in silico* profile. Scaffolds **S-13–S-16** all examined the replacement of the nitrogen in the 1-position with oxygen. All these compounds showed some advantage over **3** and so were targeted for synthesis, although **S-15** was synthesized without the fluoro substituent and **S-16** was not synthesized due to lack of a tractable route.

**Table 4:**
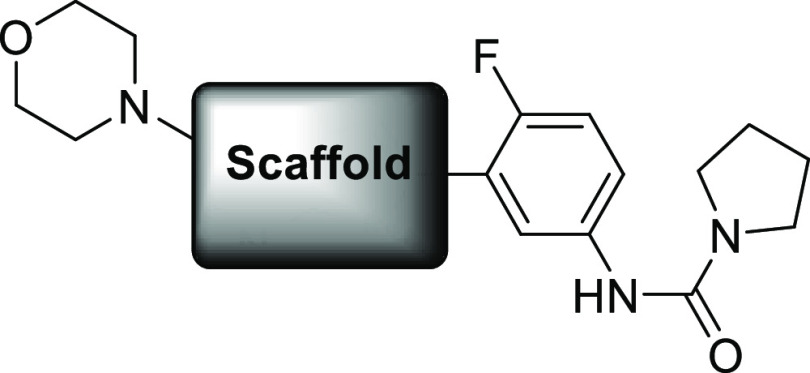

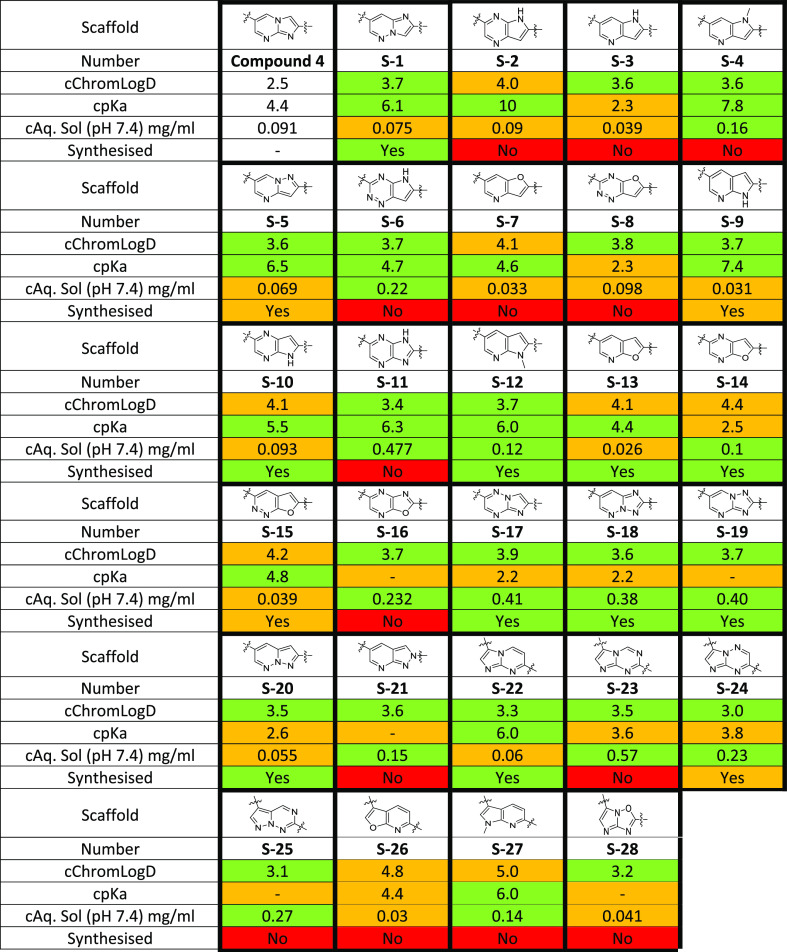
Scaffolds Identified by the Medicinal Chemistry Team for *In Silico* Profiling^a^

a cChromLogD calculated using GSK predictive software. Colored as green <4 and amber >4. cp*K*_a_ calculated using ACD labs p*K*_a_ calculator for the most basic nitrogen on the bi-cycle. Colored as green if >compound **4** (4.4) and amber if <compound **4**. cAq. Sol (pH 7.4) mg/mL is GastroPlus calculated aqueous solubility at pH 7.4, colored green if >compound **4** and amber if ≤compound **4**. Synthesis color scheme: green = desired compound synthesized, orange = alternative analogue synthesized (e.g., no F or phenyl instead of morpholinyl), red = not synthesized (no successful route or abandoned due to SAR learnings).

Scaffolds **S-17–S-21** all retained the HBA nitrogen in positions 1 and 8 and examined the effect of varying the heteroatoms in the other positions around the rings. All of them showed some potential advantage over **3** and so were selected for synthesis (although a tractable synthesis of **S-21** was not successfully developed).

Scaffolds **S-22–S-27** all examined the reversal of the 6,5-fused scaffold to a 5,6-fused scaffold. **S-22** had a cChromLogD below 4 and higher predicted p*K*_a_ compared to **3** and so was selected for synthesis. While **S-23–S-25** all had higher predicted solubility, they proved to be synthetically challenging and we only developed a tractable synthetic route to **S-24** with phenyl, rather than the morpholine substituent. **S-26**, which replaced the nitrogen in the five-membered ring with oxygen, had a poor *in silico* profile and so was not synthesized. Although **S-27** had predicted improved solubility, it was not synthesized due to our understanding that the *N*-methyl group would not be tolerated. Finally, we profiled some 5,5-bi-cycles, as exemplified by **S-28**. The only advantage this might confer would be a cChromLogD below 4, as the predicted solubility was lower than **3**, and the core did not contain the basic nitrogen. Because of this, and also because the vectors of the substituents would be very different to **3**, as well as a lack of literature precedent for synthesis, we elected not to synthesize this analogue.

This led to a set of 10 scaffolds synthesized with the desired substituents, plus a further 4 which were synthesized with alternative substituents (Table 5). From this, we gained some key SAR learnings. Scaffolds **S-1** (compound **15**) and **S-5** (compound **16**) showed that removing the HBA nitrogen from position 1 led to inactive compounds in the INMAC assay. Scaffolds **S-9** and **S-10** showed that a HBD in position 1 was not tolerated (compounds **17** and **18**, respectively). Scaffold **S-12** (compound **19**), with *N*-Me in the 1-position, was inactive. Scaffolds **S-13–S-15** (compounds **20–22**) which replaced the HBD nitrogen with oxygen showed a range of potencies, although all had poor aqueous solubility compared to **3**, with no improvement in FaSSIF solubility either. Scaffolds **S-17–S-20** (compounds **23–26**), which retained the HBD nitrogens in positions 1 and 8, all maintained good activity, with pEC_50_ values of 6 or above. Scaffolds **S-17**, **S-18**, and **S-19** (compounds **23**, **24**, and **25** respectively) all showed a much improved FaSSIF solubility compared to **3**, with **24** and **25** having overall very similar profiles. The **S-19** scaffold has recently been published by Novartis.^8,26^ Although **S-20** (compound **26**) did have good potency, it showed no improvement in FaSSIF solubility compared to **3**. The compounds with a “reversed” bi-cycle (**S-22** and **S-24**, compounds **1** and **27**, respectively) proved to be very interesting. Compound **27** was only synthesized as the phenyl analogue; although this showed similar potency to **3**, it had very poor FaSSIF solubility. On the other hand, **1** was only slightly less potent than **3** and had the highest FaSSIF solubility of all the scaffold hop compounds. Based on these findings, compounds **23** (**S-17**) and **1** (**S-22**) were investigated further (**23** was selected over **24** as it proved much more straightforward to scale-up the synthesis to get suitable quantities for *in vivo* studies). The progression of **23** was subsequently stopped as the aniline resulting from the hydrolysis of the urea was positive in the Ames assay,^10^ indicating a potential genotoxicity issue. This led us to focus on **1** as the most promising scaffold for further development; although it was slightly less potent than **23**, it was the compound with the best solubility profile and had high stability in mouse liver microsomes.

**Table 5:**
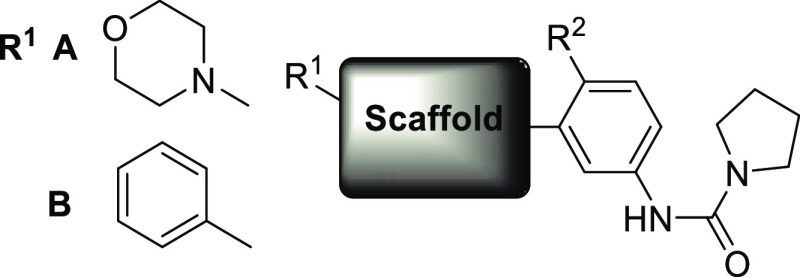

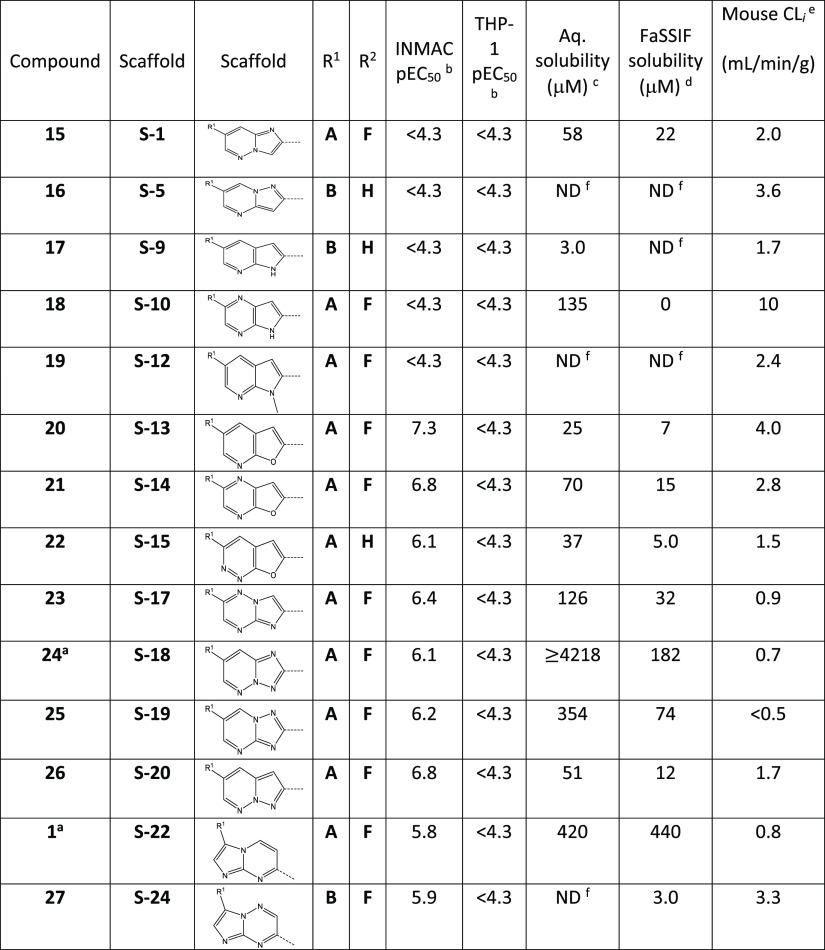
Effect of Scaffold Hops on Activity and Physicochemical Properties

a Data reported previously for **1** and **24**.^10^

b INMAC is the intramacrophage assay carried out in THP-1 cells with *L. donovani* amastigotes. Data are the result of at least three independent replicates and standard deviations are ≤0.4.

c Aqueous solubility is the kinetic solubility measured by CLND.

d FaSSIF solubility is the fasted state simulated intestinal fluid solubility.

e Cl_i_ is the mouse liver microsomal intrinsic clearance.

f ND means not determined.

**Figure 4:**
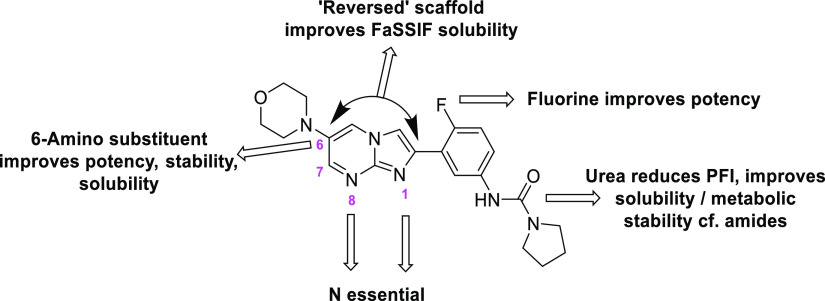
Summary of the SAR around the core scaffold.

To summarize the SAR, a HBD was essential in positions 8 (N) and 1 (N or O), whereas a HBD in the 7-position was detrimental to activity (Figure 4). A 6-substituent, particularly morpholine, was essential for activity and metabolic stability.^10^ In the other positions around the ring (e.g., 3, 4, 5, 7, and 9) heteroatoms were tolerated but not essential to activity. Regarding the central phenyl ring, 4-fluoro boosted activity and 1-urea gave a better balance of potency, solubility, and metabolic stability than the corresponding amides (data reported previously).^10^ Finally, it proved possible to reverse the central bi-cycle, a change which had a small detrimental effect on potency that was compensated for by the improvement in FaSSIF solubility.

A key aim of the scaffold-hopping exercise was to identify compounds with improved solubility, in both aqueous and FaSSIF media. Of the 10 scaffolds synthesized with the same substituents as **4**, only three showed an improvement in solubility in both media (**24**, **25**, and **1**), with **23** showing an improvement only in FaSSIF solubility (**S-18**, **S-19**, **S-22**, and **S-17**, respectively). Compounds **23**, **24**, and **25** all had better *in silico* predictions for solubility than for compound **4**, whereas **1** was predicted to be similar. However, compound **1** had much better solubility than compound **4**, and of these four compounds, it had the highest FaSSIF solubility. Interestingly, scaffold **S-22** was predicted to have a much higher pKa than **S-17** and **S-18**, indicating that for compound **1** a significantly higher proportion is likely to be protonated at physiological pH. Therefore, while the *in silico* profiling helped to triage the number of scaffolds for synthesis, it was clear that a large number of scaffolds needed to be synthesized in order to identify the optimal scaffolds for progression.

Because reversing the imidazo[1,2-*a*]pyrimidine scaffold (compounds **1** and **27**) was liable to orient the pendant amine and urea substituents slightly differently, we further explored the SAR around the 3-position of the bi-cycle, where morpholine was substituted (Table 6). The replacement of morpholine of **4** with phenyl (**28**) gave an expected increase in potency alongside a decrease in solubility, while switching to pyridyl (**29**) maintained the improved potency alongside an increase in FaSSIF solubility compared to **28**. However, the Cl_i_ was above our targeted level of <1 mL/min/g for a preclinical candidate. We were interested in exploring saturated substituents; isopropyl **30** showed a similar potency to morpholine but with poorer FaSSIF solubility, and isobutyl **31** showed a small improvement in potency but with poorer metabolic stability. In order to improve solubility, we explored the introduction of a basic center with methylene-linked morpholine **32** and directly linked morpholine **33**. While this was successful in delivering compounds with improved FaSSIF solubility compared to **1**, it unfortunately led to a loss of potency.

**Table 6:**
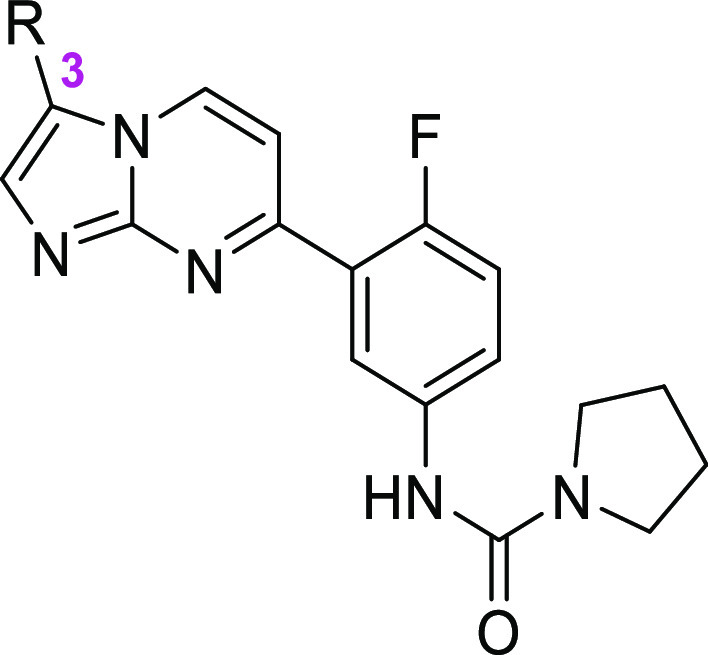

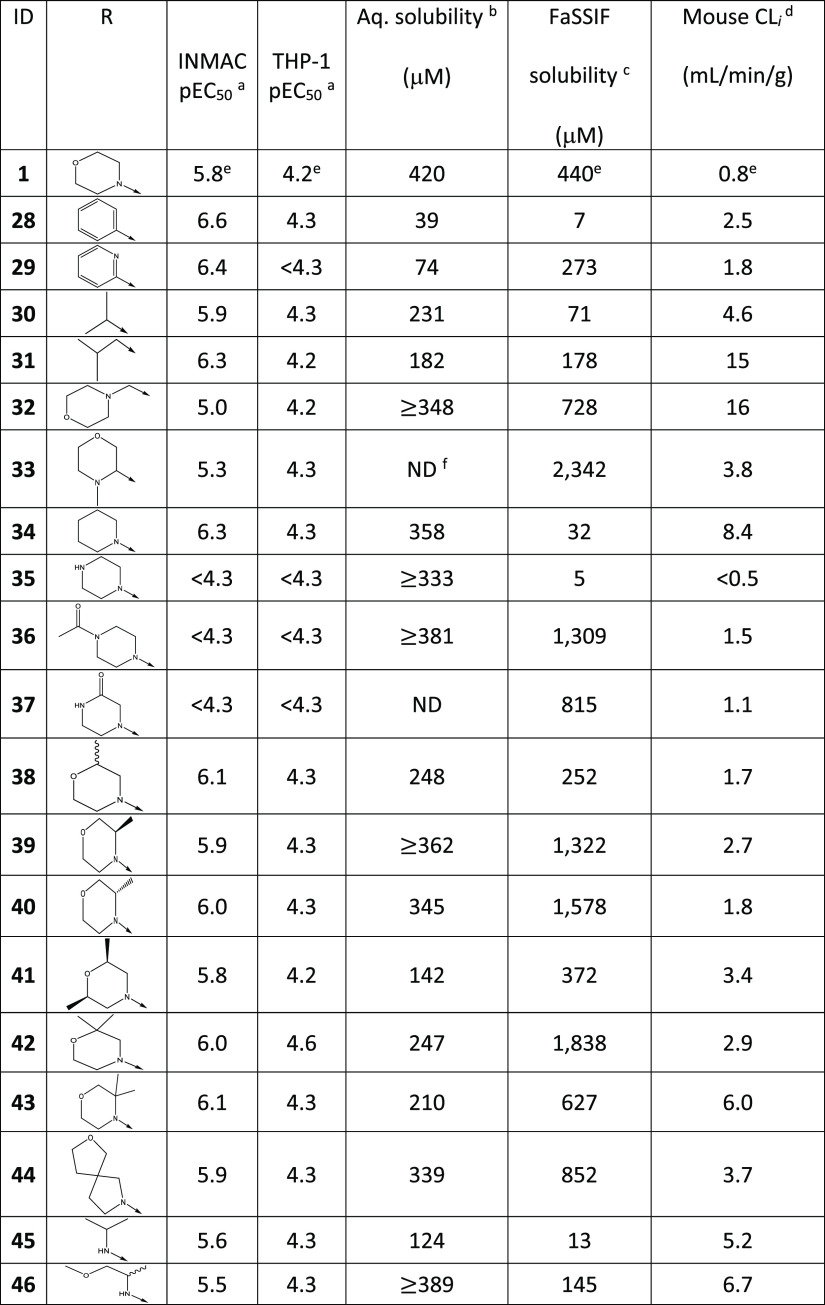
Effect of Modifications or Replacements to the Morpholine Group on Activity and Physicochemical Properties

a INMAC is the intramacrophage assay carried out in THP-1 cells with *L. donovani* amastigotes. Data are the result of at least three independent replicates and standard deviations are ≤0.4.

b Aq. solubility is the kinetic solubility measured by CLND.

c FaSSIF solubility is the fasted state simulated intestinal fluid solubility.

d Cl_i_ is the mouse liver microsomal intrinsic clearance.

e Data reported previously.^10^

f ND means not determined.

We also further explored N-linked amines, where piperidine **34** showed good potency but with poor FaSSIF solubility. Piperazines **35–37** were inactive, although **36** and **37** did show enhanced FaSSIF solubility. As we had seen success with methylmorpholines in other series,^6,7^ we synthesized rac-2-methyl morpholine **38** and the enantiomeric 3-methylmorpholines **39** and **40**. All of these compounds showed similar potency to **1** and improved FaSSIF solubility, although all the three were less metabolically stable. Likewise, dimethylmorpholines **41**, **42**, and **43** were synthesized, all showing similar potency to **1** but with poorer metabolic stability, although **42** and **43** did show improved FaSSIF solubility. Spiro analogue **44** again was less metabolically stable than **1**, and secondary amines **45** and **46** showed a drop in potency compared to **1**.

From all the *in vitro* profiling, none of the changes to the morpholine ring led to compounds with an improved overall profile; therefore, **1** was identified as the most suitable compound for further *in vitro* and *in vivo* studies. As reported previously,^10^ a PK study of **1**, dosed at 25 mg/kg po, supported its progression into the infected mouse model, where it showed a candidate level efficacy against VL at this dose. When dosed orally twice a day for 10 days, **1** had an ED_90_ of 16 mg/kg and ED_99_ of 30 mg/kg. The compound had suitable PK for oral delivery and a good predicted safety margin of at least 37-fold when dosed in rats at 300 mg/kg. Critically, unlike compounds **4** and **23**, both **1** and the aniline derivative potentially released by hydrolysis of its urea were negative in the Ames assay, as reported previously.^10^ Alongside the previously reported safety profiling, **1** was also screened against a representative set of three kinases (LCK, PI3Kγ, and AuroraB) and was inactive against them all (pIC_50_ <4.5). The compound is now being advanced for the first time in human studies for VL.

### Modeling

**Figure 5:**
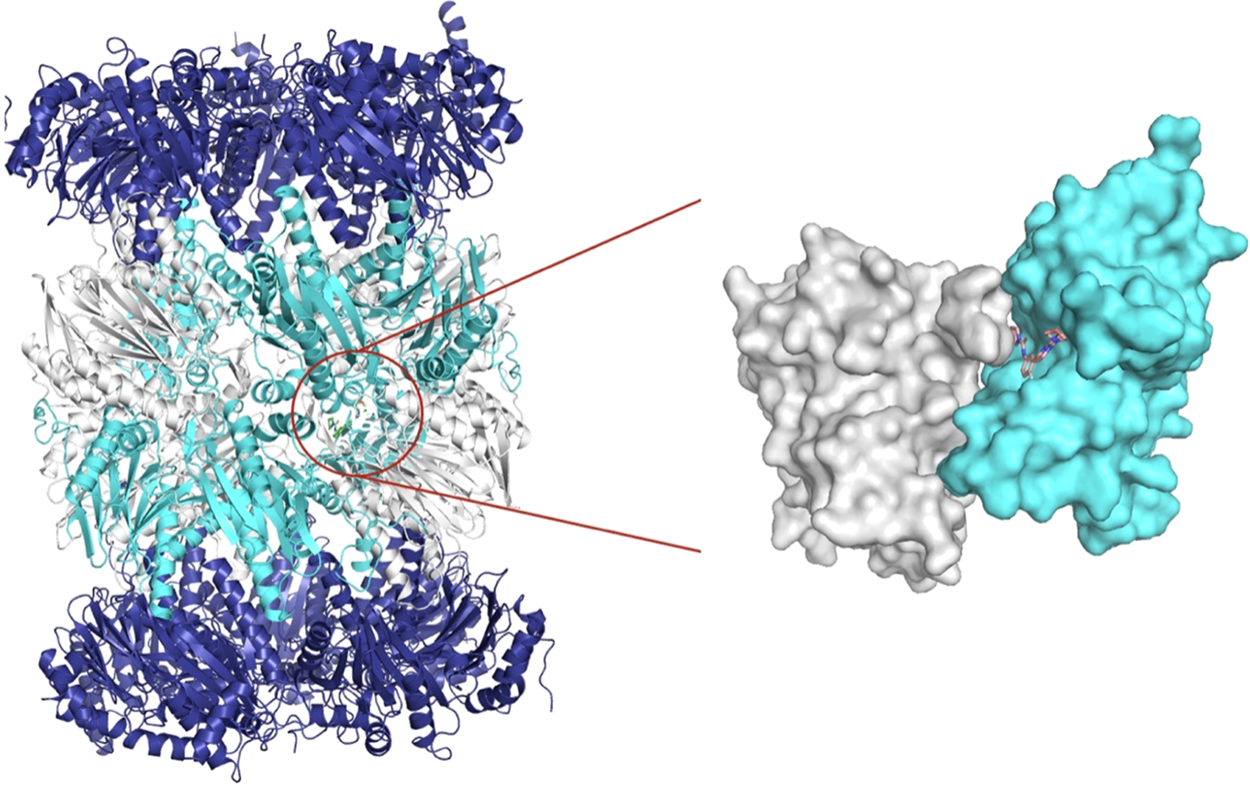
Compound **1** bound to the β4 (light blue) and β5 (white) subunits of the *L. tarentolae* 20S structure. PDB: 6QM7.

In parallel to these phenotypic optimization studies, attempts to identify the target of these series were carried out as previously reported.^10^ It was found that these compounds act through the inhibition of the chymotrypsin-like activity catalyzed by the β5 subunit of the *L. donovani* proteasome, demonstrating good selectivity over the human enzyme. A high-resolution cryo-EM structure of compound **1** bound to the *Leishmania tarentolae* proteasome^10^ revealed a binding site that lies between the β4 and β5 subunits (Figure 5) and exploits an induced cavity that is lined on one side by β4 residues that are divergent between humans and kinetoplastid protozoan. The cryo-EM structure of a related compound in the complex with the *Leishmania tarentolae* proteasome has also been recently published by Novartis.^9^

### Description of the Binding Mode of Compound **1**

**Figure 6:**
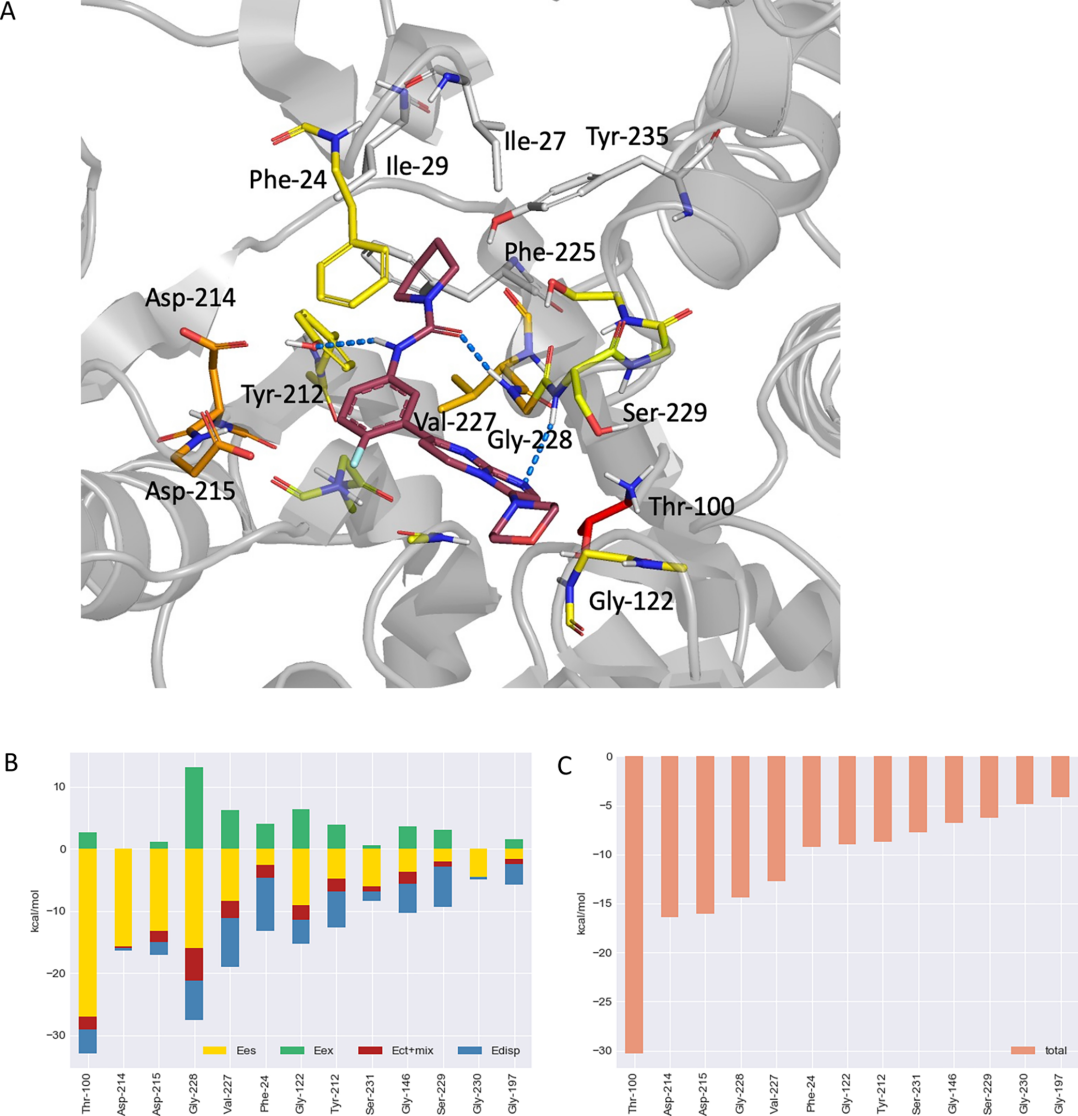
(A) View of compound **1** binding in the *L. donovani* 20S proteasome model β4 and β5 subunits. Shown in stick form are the 12 residues that have a FMO-PIE <−3 kcal/mol; colored red if FMO-PIE <−30 kcal/mol, orange if between −30 and −10 kcal/mol, and yellow if FMO-PIE between −10 and −3 kcal/mol. (B) FMO-PIE decomposition analysis results showing, for each residue within 5 Å, the contribution of the four energy terms: electrostatic (**yellow** bar), exchange-repulsion (**green** bar), charge transfer (**red** bar), and dispersion (**blue** bar). (C) FMO-PIE generated contribution to compound **1** binding energy per fragment residues within 5 Å from the ligand.

A homology model of compound **1** bound to the *L. donovani* 20S proteasome β4 and β5 subunits was generated using the *L. tarentolae* 20S proteasome cryo-EM structure as a template. Compound **1** binds at the interface between the β4 and β5 subunits close to the catalytic Thr100 residue (Figure 6A), which is the first residue of the β5 subunit. The pyrrolidine carboxamide sits in the most buried part of the binding site, a mainly hydrophobic cavity important for selectivity against the human orthologue. The cavity is defined by Ile27, Ile29, and Phe24 from the β4 subunit and by Phe225, Val227, and Thr235 from the β5 subunit. The urea carbonyl oxygen is hydrogen bonded to the Gly228 backbone nitrogen, and the urea nitrogen is hydrogen bonded to the hydroxyl of the Tyr212 side chain. The phenyl ring is placed on top of Gly197 of the terminal part of the β-strand 7 of the β5 subunit with the fluorine substituent facing the Ser195 side chain hydroxyl. The “top edge” (see Figure 2) of the imidazopyrimidine bi-cyclic system is mainly solvent exposed with the six-membered ring sitting on top of Gly146 and the sp^2^ nitrogen of the five-membered ring interacting with the donor NH group of Ser229. The nitrogen on the “bottom edge” of the pyrimidine ring does not make any specific interactions; however, it contributes to the charge delocalization, resulting in a favorable charge interaction with Thr100 (see later). The morpholine group is largely solvent exposed and is directed toward the β hairpin motif critical for the recognition of bortezomib (β strands 3 and 4 of the subunit β5), establishing a hydrogen bond with the Gly122 backbone NH (Figure 6A). (Bortezomib is a clinically used inhibitor of the human proteasome.)

### Computational Analysis of Binding Interactions

The fragment molecular orbital method (FMO-MP2)^27^ was used to calculate the interaction energy between compound **1** and the proteasome and to gain a deeper understanding of the molecular recognition event. Being a quantum-mechanical (QM)-based method, FMO-MP2 is capable of detecting and accounting for nonclassical interactions that are poorly parameterized in molecular mechanics-based force fields (e.g., CH−π, halogen−π, cation−π interactions, and nonclassical hydrogen bonds) resulting in a more accurate binding energy. To overcome the high computational costs associated with the QM calculation, the system is fragmented into smaller parts and QM calculations are performed on each individual fragment pair to derive pairwise interaction energy (PIE) between each of the fragments and the ligand. By combining the PIE of the ligand and the fragments, it is possible to derive the total interaction energy of the ligands with the target. The decomposition analysis of the PIE (PIEDA)^28^ allows the derivation of four different energy terms (electrostatic, charge transfer, dispersion, and exchange-repulsion) that provide a deeper residue-by-residue insight into the nature of the ligand interaction with the target. The electrostatic and charge transfer terms are dominant in H-bonds, polar (favorable and unfavorable) interactions, and salt bridges. The dispersion term is more prominent in hydrophobic and van der Waals interactions. The exchange-repulsion term describes the steric-repulsion between electrons of different atoms accounting for steric clashes.

The FMO-PIEDA analysis shows that, out of the 40 residues in a 5 Å range from the ligand, there are 13 contributing significantly (PIE < −3 kcal/mol) to the overall calculated binding energy (Figure 6B,C). The initial work focused on analyzing the H-bonding and hydrophobic interactions revealed by the cryo-EM structure; the contribution to the binding energy from the residues directly involved in hydrogen bonding with the ligand was confirmed. The Gly228 hydrogen bond to the urea carbonyl is the strongest H-bonding interaction with a calculated electrostatic energy term of −15.9 kcal/mol (PIE −14.3 kcal/mol).

In the cryo-EM structure of the *L. tarentolae* structure, a CH_2_ group next to morpholine oxygen is adjacent to NH of Gly 122, which is a part of a semi-flexible loop. However, in the model of the *L. donovani* enzyme, the morpholine twists slightly, allowing N–H of Gly 122 to establish a strong hydrogen bond with the morpholino oxygen atom (PIE −9.0 kcal/mol, electrostatic term −9.0 kcal/mol). For Tyr212, the PIE energy is −8.7 kcal/mol which is the result of both the hydrogen bonding (electrostatic) with the urea NH and the favorable hydrophobic interaction with the pyrrolidine ring (dispersion). The FMO-PIEDA result also shows that the Ser229 interaction (PIE −6.26 kcal/mol) is mainly due to the favorable interaction between the β carbon atom of the side chain (dispersive interaction) with the heterocyclic scaffold of the ligand rather than the hydrogen bonding of the nitrogen in the 5-membered ring of imidazopyrimidine with the backbone NH. This is consistent with the poor geometry observed for the hydrogen bond present in both the *L. tarentolae* cryo-EM structure and the *L. donovani* model. The favorable contribution of hydrophobic contacts is also significant for Gly197 (−4.2 kcal/mol) and Gly146 (−6.8 kcal/mol) as both residues are in contact with the central phenyl ring. The contact between the pyrrolidine carboxamide and the Phe24 side chain has a contribution of −9.2 kcal/mol.

**Figure 7:**
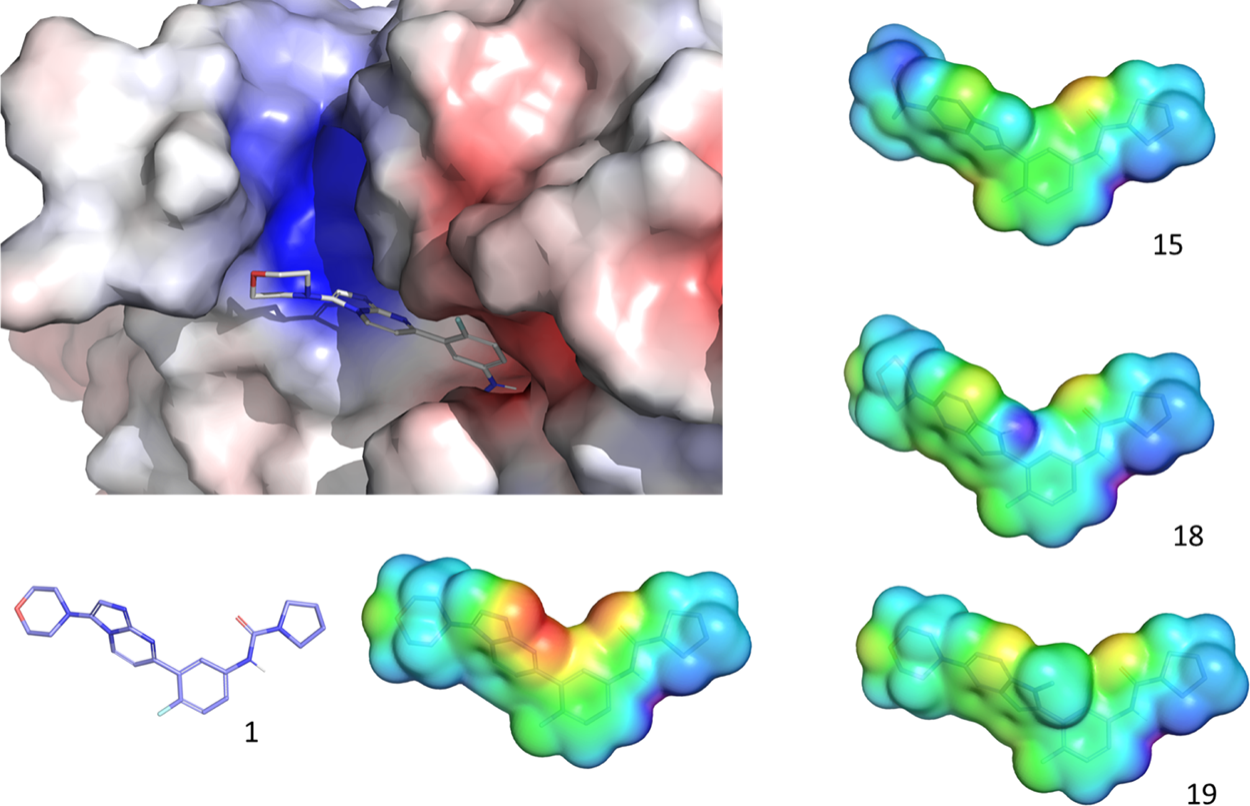
Molecular ESP maps for some of the studied analogues. Protein surfaces were generated using the APBS plugin for PyMol and colored by calculated charges (red −5 kbT/ec, blue +5 kbT/ec). The ligand surfaces were generated using Jaguar in Schrödinger and colored by the electrostatic potential (red −80 kcal/mol, blue +75 kcal/mol).

Importantly, in addition to the interactions that could be identified from a visual inspection of the cryo-EM structure, the FMO-PIE analysis highlighted other residues that establish nonintuitive interactions and are important in the molecular recognition event. Thr100 (PIE −30.3 kcal/mol), Asp214, and Asp215 (PIE −16.4 and −16.0 kcal/mol, respectively) are indicated as the residues with the strongest interaction with the ligand. These are long-range electrostatic interactions between the protein and the unevenly distributed electrons of the ligand. The analysis of the electrostatic surface potential (ESP) of the protein binding site highlighted a positively charged patch consisting of the positively charged terminal Thr100 and the backbone amide of Ser229. A charge–dipole interaction is established between this positively charged patch of the protein with the negatively charged sp^2^ nitrogen atoms of the imidazopyrimidine scaffold in positions 1 and 8 on the bottom edge (Figures 2 and 7). A similar effect is demonstrated by the presence of the fluorine atom on the phenyl ring, which increases potency by approximately 10-fold (see compounds **4** and **6**). As previously discussed,^10^ the ESP of the ligand shows that the presence of the fluorine atom on the phenyl ring causes an accumulation of the positive charge on positions 3 and 4 of the fluorophenyl ring giving rise to another strong dipole–charge interaction with the negatively charged Asp214 and Asp215. This effect of fluorine appears to be more important to binding than its interaction with Ser195. Val227 also provides a significant contribution to binding energy (PIE −12.7 kcal/mol) with the decomposition analysis indicating an equal contribution from the dispersive term (related to the hydrophobic side chain) and electrostatic term (related to the positively charged patch localized on the pyrrolidine ring—see Figure 7). Therefore, FMO-PIE analysis showed that the binding of compound **1** to the proteasome is governed by a complex mixture of specific hydrogen bonds, stacking interactions, optimal electrostatic complementarity, and long-range electrostatic interactions between the protein and the ligand.

### Computational Analysis of Structure Activity Relationships

To analyze the effect of the imidazopyrimidine scaffold replacement in the series related to compound **1**, all the synthesized scaffold hop compounds (Table 5) were modeled in the *L. donovani* 20S proteasome model and studied by FMO-PIEDA and ESP. The compounds tended to be either active (pEC_50_ 5.8–7.3) or inactive (pEC_50_ < 4.3). Derivatives with a fluorine substituent on the phenyl ring ortho- to the bi-cycle were consistently more active than the hydrogen analogues. We and others have noted a correlation of activity between the inhibition of proteasome and activity against axenic *Leishmania* parasites.^8,10^ While there could be subtleties in INMAC activity due to physicochemical properties of compounds, this data allows an interpretation of the SAR.

**Figure 8:**
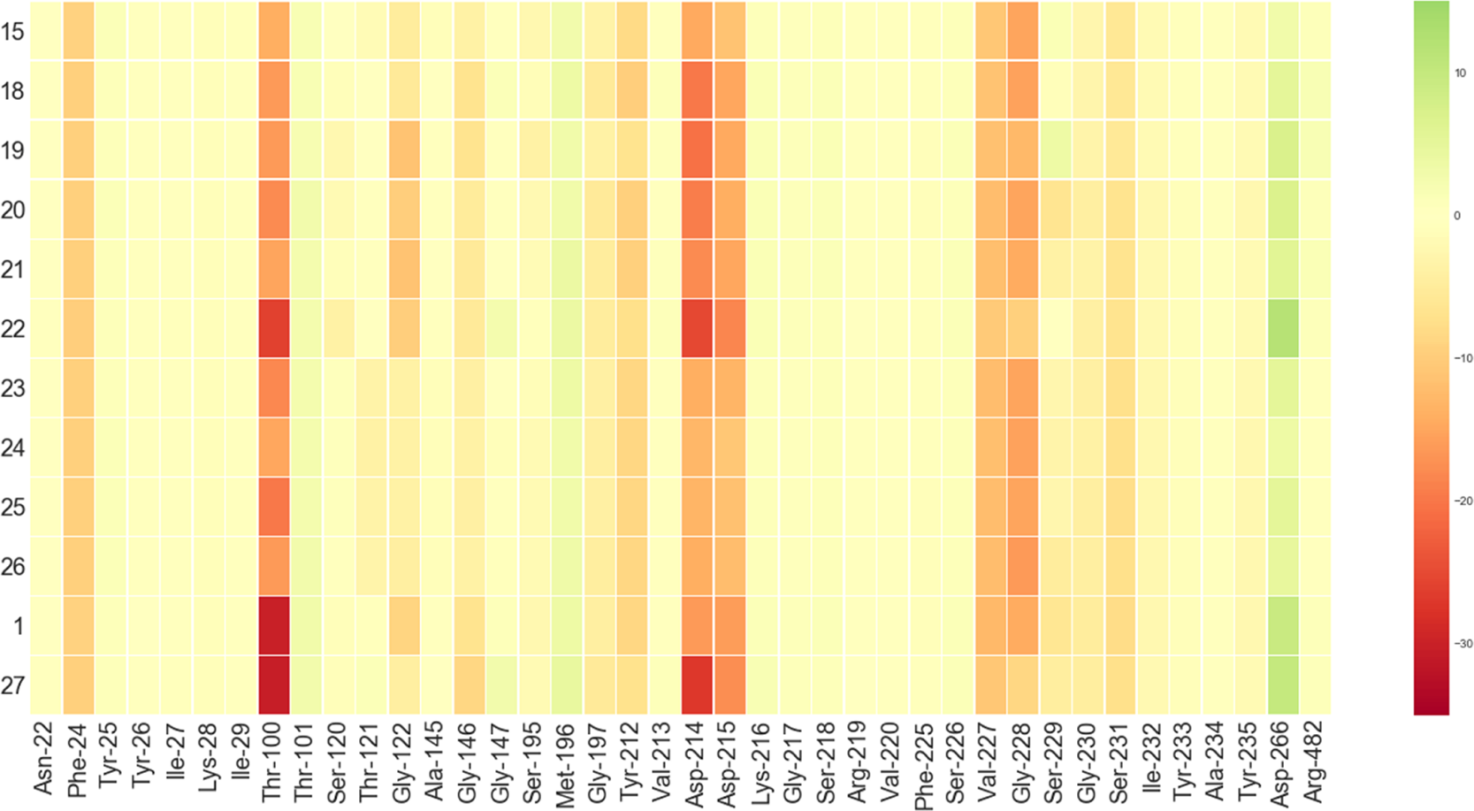
FMO-PIE generated contribution to binding energy in kcal/mol per fragment residues within 5 Å from the ligand calculated for compound **1** analogues. Red indicates favorable, whereas green indicates unfavorable interaction energies.

The modeling data generated confirmed the important role that the electrostatic complementarity between the ligands and the proteasome binding site plays in the optimization of the interaction. The ESP maps (Figure 7 and Supporting Information), clearly show that even if the hydrogen acceptor interacting with the Ser229 backbone nitrogen is conserved, where there is a lack of the negative charge accumulation on the bottom edge (positions 1 and 8) of the imidazopyrimidine scaffold (as observed for compounds **15**, **18**, and **19**), these compounds are inactive (Table 5). This is further supported by the FMO-PIEDA analysis results (Figure 8), indicating that one of the strongest interactions that compound **1** establishes is with Thr100, an interaction primarily electrostatic in nature, which contributes with −30.3 kcal/mol. Compound **27** (Table 5) is also characterized by a strong interaction between Thr100 (FMO-PIE −30.3 kcal/mol) and the negatively charged bottom face (positions 1 and 8) of the imidazotriazine scaffold. The scaffold of compound **27** also seems to enhance the partial positive charge on 3- and 4-positions of the phenyl ring and strengthen the interaction with Asp214 and Asp215 (see the ESP, Supporting Information).

**Figure 9:**
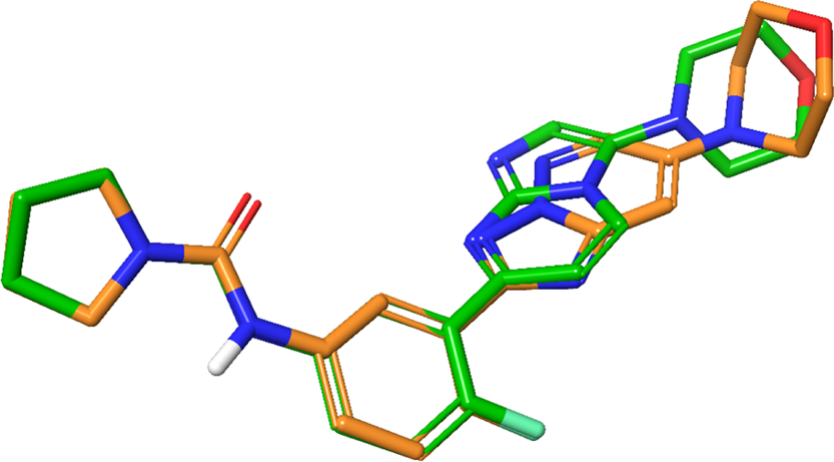
Superimposition of a 6,5-bi-cyclic system (compound **1**, shown in green) with a 5,6-bi-cyclic system (compound **24**, shown in orange).

The “reversed” 5–6 bi-cyclic system compounds **1** and **27** not only are characterized by a particularly favorable accumulation of the negative charge on position 1 of the bi-cyclic scaffold but also by a slightly different placement of the substituent in position 3 in relation to the 6–5 bi-cyclic systems of the other analogues. An inspection of the bound conformations shows that the vector of the substitution in the 3-position is similar between the 5–6 and 6–5 bi-cycles (Figure 9), but in the 5–6 system, the substituent is about half a bond longer than in the 6–5 system. This results in the morpholine ring being pushed closer to Gly122 on the β strands 3 and 4 of the subunit β5 and might explain the lower potency of the compounds with a “reversed” scaffold. Docking studies suggest that in order to avoid a steric clash with Gly122 and the beta-hairpin involved in bortezomib binding, compound **24** appears to adopt a higher energy conformation with the morpholine ring almost perpendicular to the bi-cyclic scaffold. Due to this steric requirement, and together with an unfavorable electrostatic distribution on the bi-cyclic scaffold, resulting in the lack of a partial negative charge interacting with Thr100 (Supporting Information), compounds **16** and **17**, where morpholine is replaced by a phenyl ring, failed to dock and are reported as inactive.

### Synthesis

**Scheme 1:**
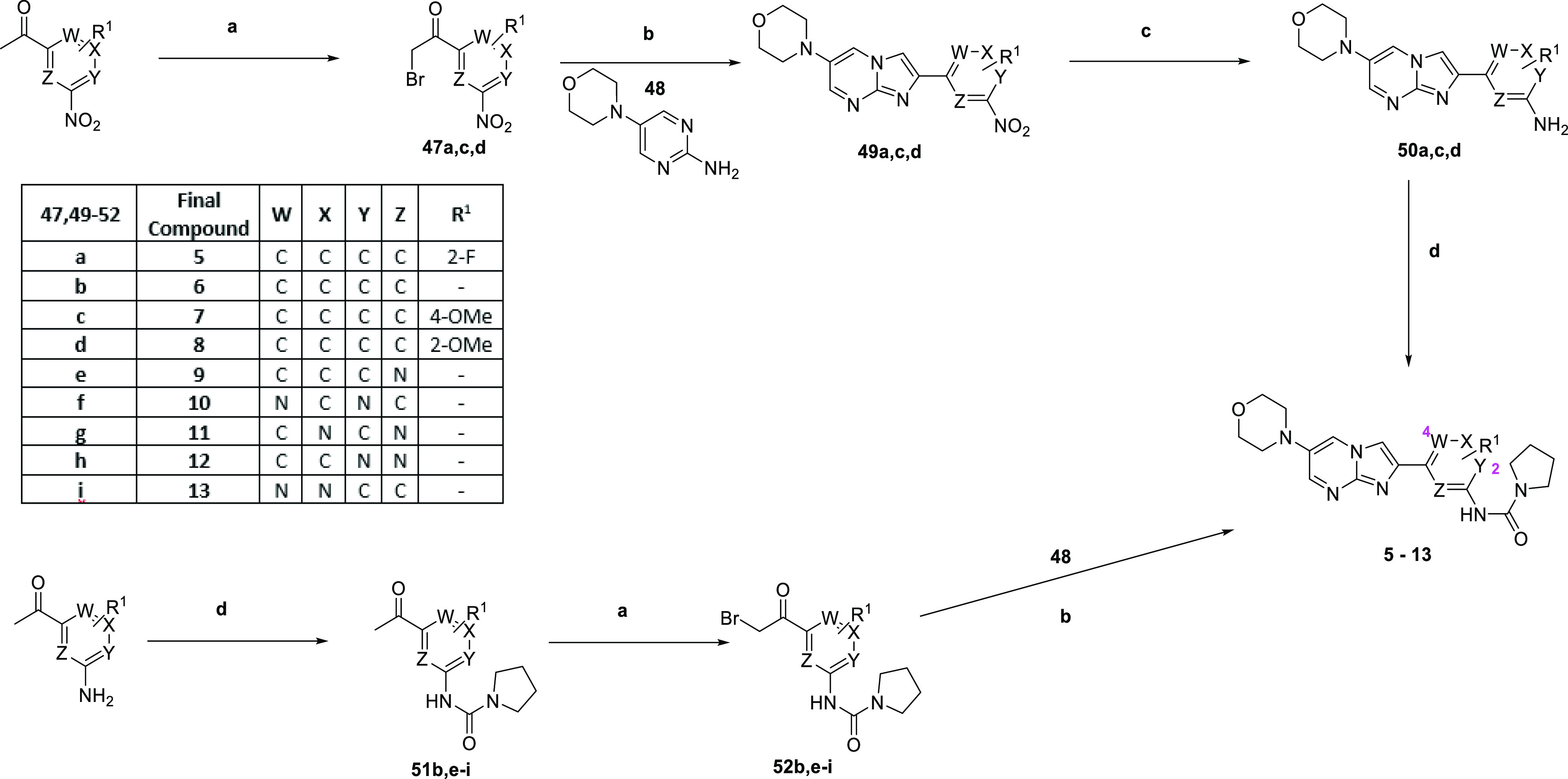
Synthesis of Compounds **5–13** Reagents and Conditions: (a) trimethyl(phenyl)ammonium tribromide, THF, RT, 18 h; (b) MeCN, 60 °C, 4 d, 43% over two steps; (c) SnCl_2_, EtOH, reflux, 2 d, quant.; and (d) pyrrolidine-1-carbonyl chloride, DMAP, pyridine, 40 °C, 2 d, 9–36%.

To explore SAR around the central phenyl ring in the 2-phenylimidazo[1,2-*a*]pyrimidin-6-morpholine sub-series (e.g., compounds **5–13**), the route shown in Scheme 1 is utilized. A suitably substituted 3-nitroacetophenone was brominated to give **47a,c,d** and then cyclized with **48** to give **49a,c,d**. The nitro reduction gave **50a–i** and subsequent urea formation with pyrrolidine-1-carbonyl chloride yielded **5**, **7**, and **8**. Alternatively, a relevant aminoheterocycle was treated with pyrrolidine-1-carbonyl chloride to give ureas **51b,e–i**, which were then brominated to give **52b,e–i**. Again, cyclization with **4**8 gave **6** and **9–13**.

All the different cores required the bespoke synthesis, as detailed in Schemes 2– 9 below (the synthesis of compounds **1, 3, 4**, and **23** was described elsewhere^10^).

**Scheme 2:**
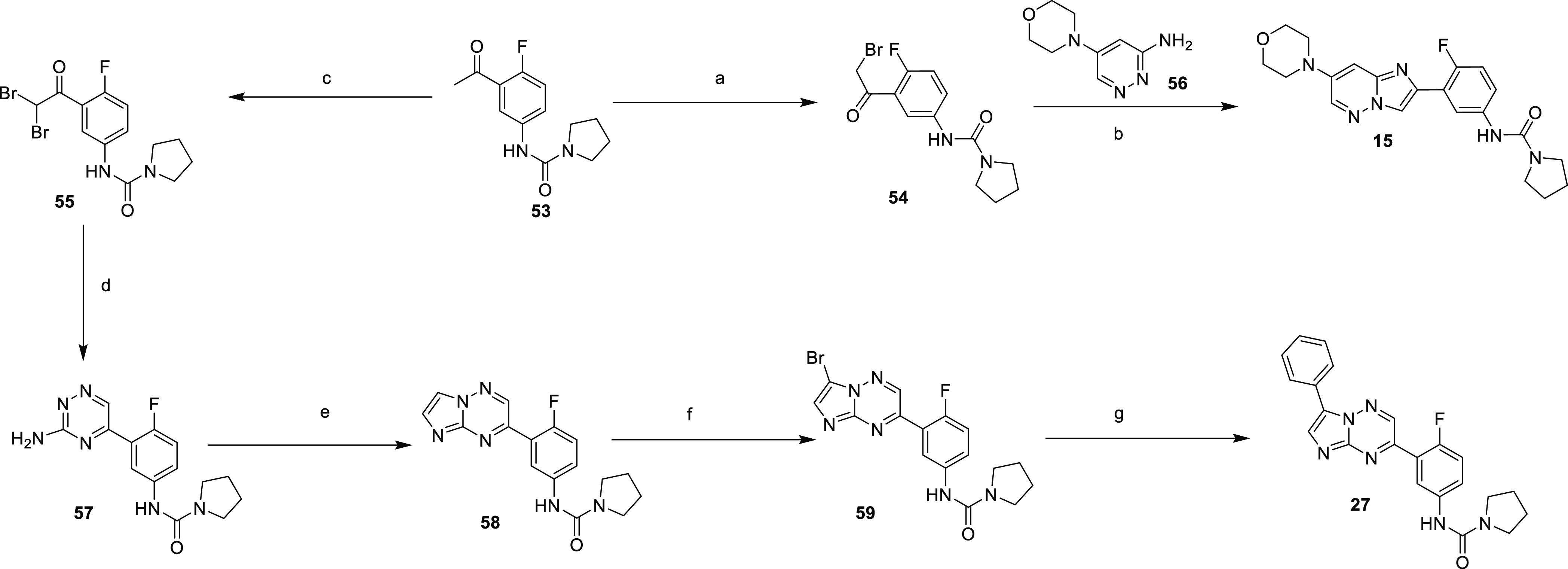
Synthesis of Compounds **15** and **27** Reagents and Conditions: (a) trimethyl(phenyl)ammonium tribromide (1 equiv.), THF, RT, 18 h, 73%; (b) MeCN, 60 °C, 2 d, 43%; (c) trimethyl(phenyl)ammonium tribromide (1 equiv), THF, 60 °C, 18 h, 46%; (d) morpholine and THF, 35 °C, 18 h, then aminoguanidine bicarbonate, acetic acid, MeOH, 60 °C, 18 h, 26%; (e) 2-bromo-1,1-diethoxy-ethane, HBr, water, 90 °C, 30 min, 36%; (f) Br_2_, NaOAc, acetic acid, RT, 1 h, 50%; (g) Pd(PPh_3_)_4_, sodium carbonate, DMF, 80 °C, 18 h, 38%.

**Scheme 3:**
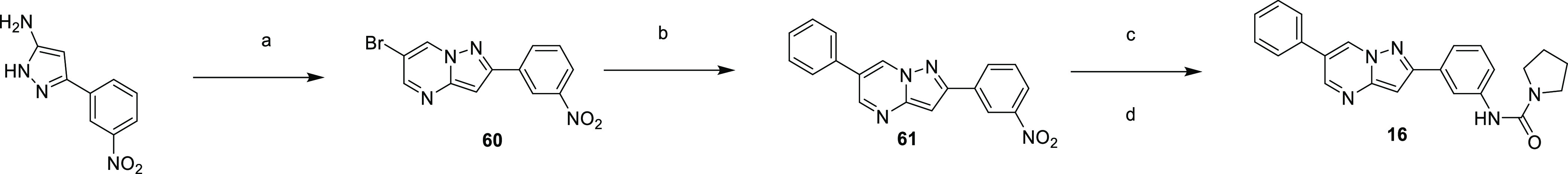
Synthesis of Compound **16** Reagents and Conditions: (a) 2-bromopropanedial, AcOH, EtOH, 75 °C, 30 min, 66%; (b) benzeneboronic acid, Pd(PPh_3_)_4_, KOAc, dioxane, 3 h, 64%; (c) Fe, NH_4_Cl, EtOH, water, 75 °C, 2 h; (d) pyrrolidine-1-carbonyl chloride, DMAP, pyridine, DCM, 70 °C, 48 h, 62% over two steps.

**Scheme 4:**
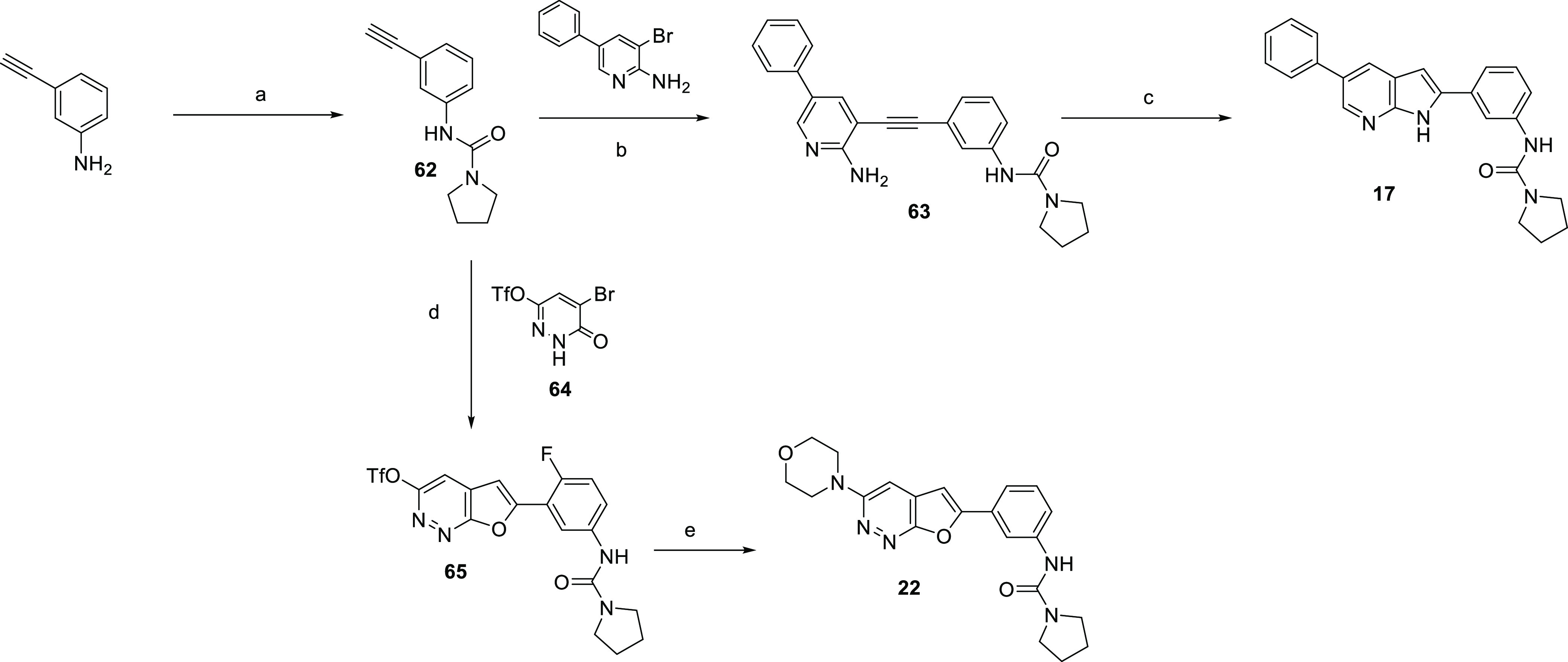
Synthesis of Compounds **17** and **22** Reagents and Conditions: (a) pyrrolidine-1-carbonyl chloride, DMAP, pyridine, DCM, 50 °C, 18 h, 79%; (b) PdCl_2_(PPh_3_)_2_, CuI, NEt_3_, DMF, 80 °C, 18 h, 46%; (c) KO*t*Bu, NMP, 75 °C, 18 h, 57%; (d) PdCl_2_(PPh_3_)_2_, CuI, NEt_3_, MeCN, RT, 6 h; (e) morpholine, 50 °C, 18 h, 12% over two steps.

**Scheme 5:**
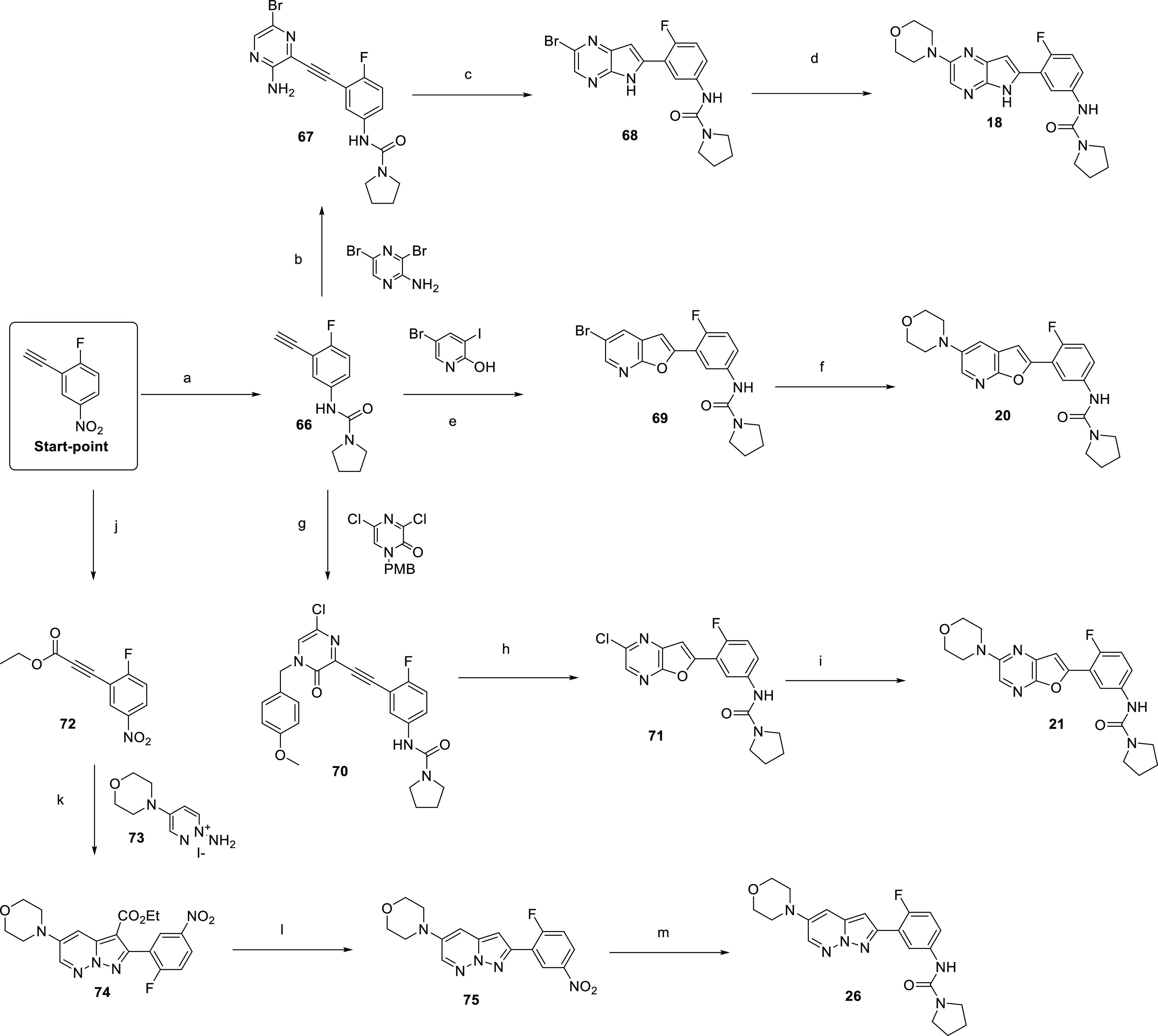
Synthesis of Compounds **18**, **20**, **21**, and **26** Reagents and Conditions: (a) (i) Fe, NH_4_Cl, EtOH, water, 75 °C, 2 h. (ii) pyrrolidine-1-carbonyl chloride, DMAP, pyridine, DCM, 50 °C, 18 h, 78%; (b) PdCl_2_(PPh_3_)_2_, CuI, NEt_3_, DMF, RT, 30 min, 23%; (c) KO*t*Bu, NMP, 75 °C, 2 h, 86%; (d) morpholine, Pd_2_dba_3_, Xphos, NaO*t*Bu, DMF, 110 °C, 2 h, 26%; (e) CuI, PdCl_2_(PPh_3_)_4_, NEt_3_, DMF, 50 °C, 16 h, 69%; (f) morpholine, RuPhos, Pd_2_(dba)_3_, KHMDS, 1,3-dioxane, 90 °C, 15%; (g) CuI, PdCl_2_(PPh_3_)_2_, NEt_3_, DMF, 80 °C, 2 h, 81%; (h) Silver nitrate, TFA, DCM, RT, 0.5 h, 89%; (i) morpholine, Pd_2_dba_3_, Xphos, NaO*t*Bu, DMF, 100 °C, 1 h, 33%; (j) LDA, ethyl chloroformate, THF, −78 °C, 2 h, 91%; (k) DBU, MeCN, RT, 18 h, 26%; (l) HBr, 120 °C, 1 h, 26%; (m) (i) iron, NH_4_Cl, EtOH, water, 80 °C, 4 h. (ii) Pyrrolidine-1-carbonyl chloride, DMAP, pyridine, DCM, 50 °C, 18 h, 24% over three steps.

**Scheme 6:**
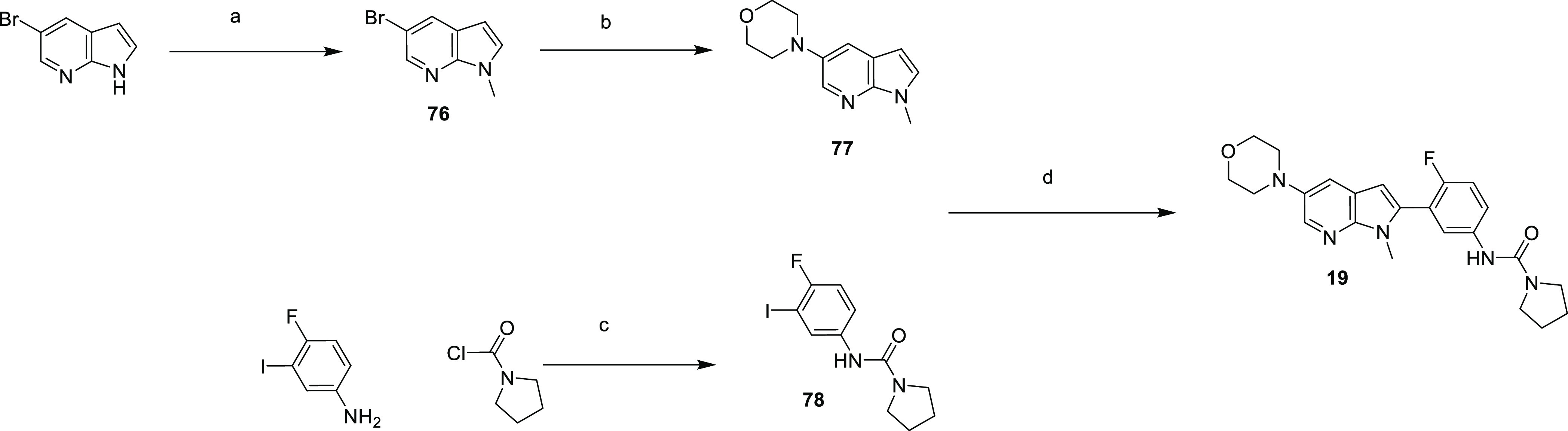
Synthesis of Compound **19** Reagents and conditions: (a) NaH, MeI, THF, 0 °C, 5 h, 70%; (b) morpholine, NaO*t*Bu, Pd_2_(dba)_3_, xantphos, toluene, 80 °C, 18 h, 48%; (c) DMAP, pyridine, DCM, 50 °C, 18 h, 82%; (d) Pd(OAc)_2_, 2-nitrobenzoic acid, Ag_2_O, DMF, 90 °C, 18 h, 2%.

**Scheme 7:**
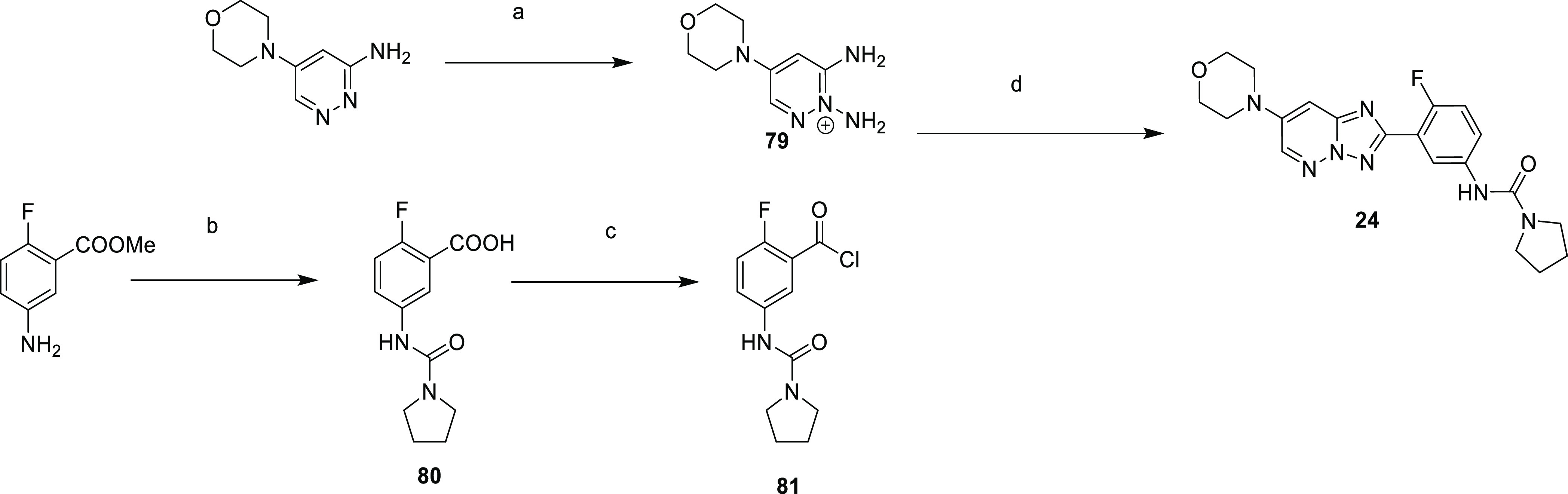
Synthesis of Compound **24** Reagents and conditions: (a) *O*-(mesitylsulfonyl)hydroxylamine, DCM, MeOH, 0 °C, 10 min; (b) (i) pyrrolidine-1-carbonyl chloride, DMAP, pyridine, DCM, 60 °C, 3 h; (ii) NaOH, water, MeOH, RT, 18 h, 64% over two steps; (c) SOCl_2_, DCM, 50 °C, 1 h; (d) DIPEA, MeCN, 80 °C, 24 h, 13% over two steps.

**Scheme 8:**
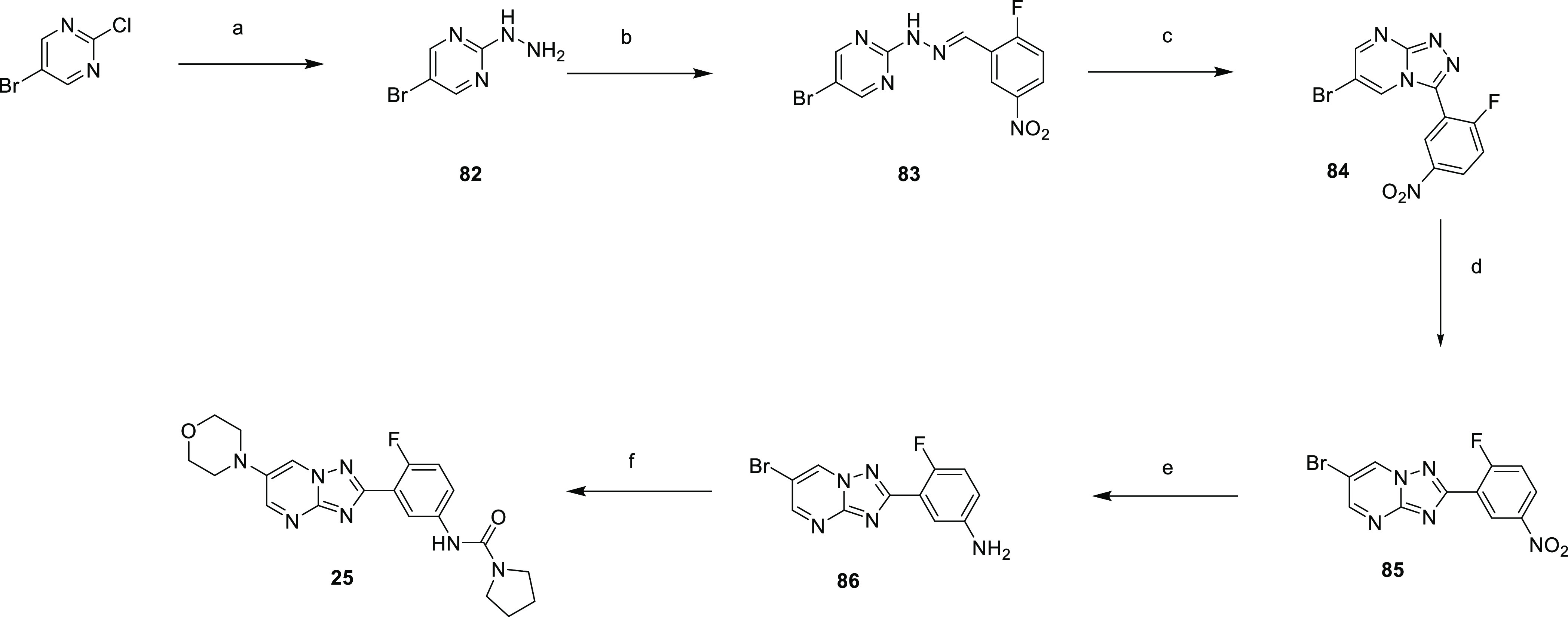
Synthesis of Compound **25** Reagents and conditions: (a) hydrazine hydrate, MeOH, 80 °C, 18 h, 99%; (b) 2-fluoro-5-nitrobenzaldehyde, EtOH, RT, 18 h, 93%; (c) iodobenzene diacetate, DCM, RT, 18 h, 87%; (d) formic acid, reflux, 5 h, 90%; (e) iron, NH_4_Cl, EtOH, THF, water, 75 °C, 18 h, 79%; (f) (i) morpholine, 100 °C, 1 h. (ii) pyrrolidine-1-carbonyl chloride, DMAP, pyridine, 50 °C, 18 h, 29%.

**Scheme 9:**
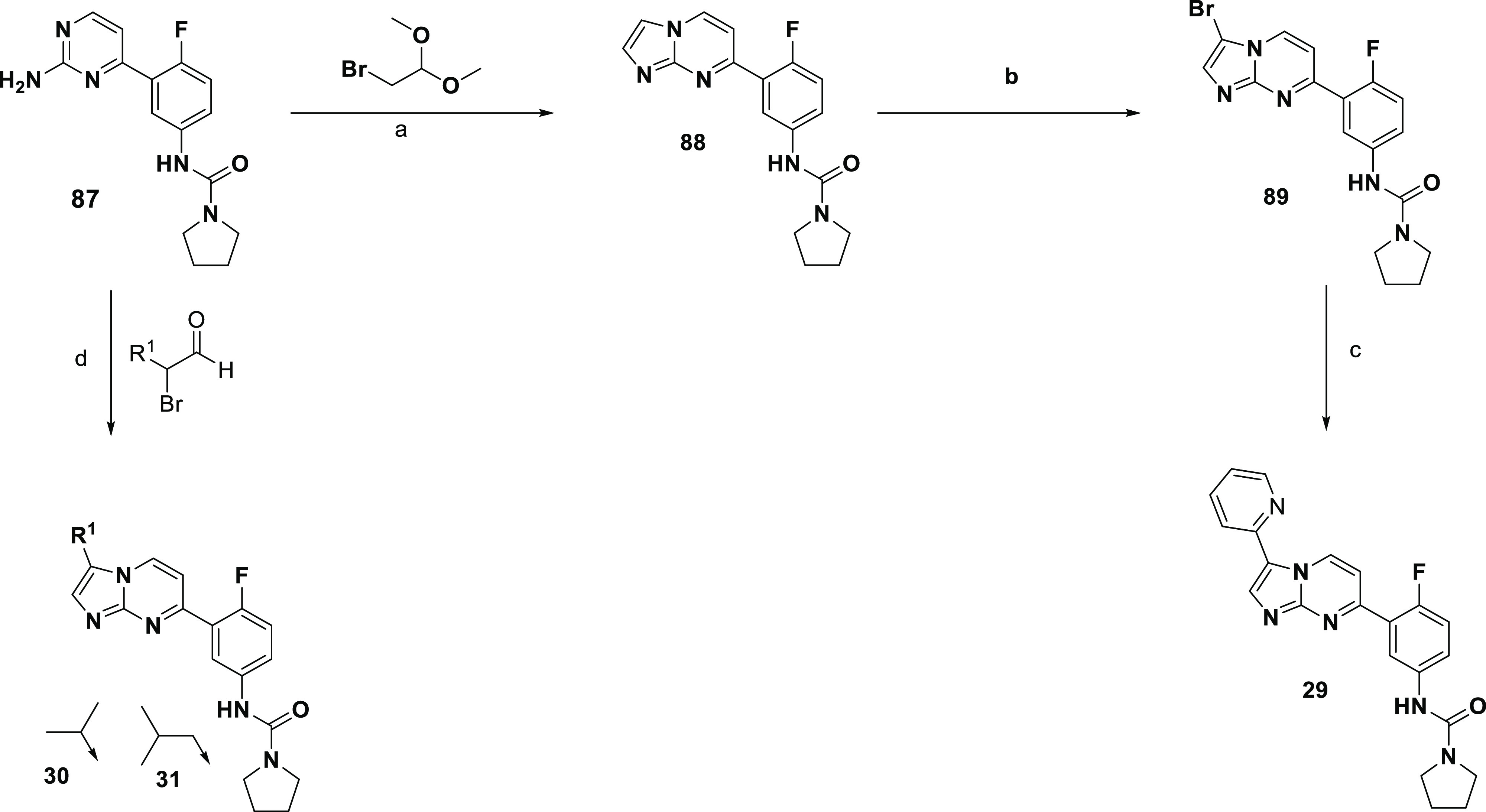
Synthesis of Compound **29–31** Reagents and conditions: (a) HBr, EtOH, 80 °C, 9 h; (b) Br_2_, NaOAc, MeOH, RT, 0.5 h, 34% over two steps; (c) 2-pyridineboronic acid, Pd(dppf)Cl_2_·CH_2_Cl_2_, K_2_CO_3_, 1,4-dioxane, water, 100 °C, 10 h, 10%; (d) EtOH, reflux, 4 h, 25–59%.

Compounds **15** and **27** are prepared, as outlined in Scheme 2. Compound **53** could be brominated with trimethyl(phenyl)ammonium tribromide to yield either monobromo **54** or dibromo **55**. The thermal cyclization of **54** with pyridazine **56** led to **15**, whereas **55** could be cyclized with 1-aminoguanidine to give **57** which was further cyclized with 2-bromo-1,1-diethoxyethane to **58**. Bromination to give **59** was followed by Suzuki coupling with benzeneboronic acid to yield **27**.

Compound **16** is synthesized according to Scheme 3, where commercially available 3-(3-nitrophenyl)-1*H*-pyrazol-5-amine was cyclized with 2-bromopropanedial to give **60**, which with subsequent Suzuki coupling with benzeneboronic acid gave **61**. The nitro group was then reduced, with subsequent urea formation to give **16**.

The synthesis of compounds **17** and **22** started from 3-ethynylaniline, as shown in Scheme 4. The urea formation to give **62** was followed by Sonagashira coupling with 3-bromo-5-phenylpyridin-2-amine to give **63**, which underwent base-catalyzed cyclization to give **17**. Alternatively, cyclization of **62** with 5-bromo-6-oxo-1,6-dihydropyridazin-3-yl triflate **64** gave **65**, with subsequent thermal displacement of the triflate with morpholine yielding **22**.

The synthesis of compounds **18**, **20**, **21**, and **26** all started from 2-ethynyl-1-fluoro-4-nitrobenzene, as outlined in Scheme 5. Initially, **66** was synthesized from 2-ethynyl-1-fluoro-4-nitrobenzene by nitroreduction and subsequent urea formation. Compound **18** was then prepared *via* Sonagashira coupling of **66** with 3,5-dibromopyrazin-2-amine to give **67**, followed by a base-catalyzed cyclization to give 6,5-bi-cycle **68**. Buchwald–Hartwig cross-coupling with morpholine then led to **18**. Alternatively, acetylene **66** could be cyclized with 5-bromo-3-iodopyridin-2-one to give **69**, which was converted to **20***via* Buchwald–Hartwig cross-coupling. To synthesize **21**, Sonagashira coupling of **66** with 1-PMB protected 3,5-dichloropyrazin-2(1*H*)-one yielded **70**, which could be cyclized to **71** in the presence of silver nitrate and TFA. Again, Buchwald–Hartwig coupling facilitated the conversion of **71** to **21**. Finally, to synthesize **26**, 2-ethynyl-1-fluoro-4-nitrobenzene was treated with LDA then ethyl chloroformate to give **72**, with base-catalyzed cyclization with 1-amino-4-morpholinopyridazin-1-ium iodide **73** leading to **74**. Subsequent decarboxylation to give **75** was followed by nitro reduction and urea formation, to yield **26**.

The preparation of **19** is reported in Scheme 6. First, 5-bromo-1*H*-pyrrolo[2,3-*b*]pyridine was methylated on the pyrrole nitrogen to give **76**, with Buchwald–Hartwig coupling leading to **77**. Alongside this, 4-fluoro-3-iodoaniline was treated with pyrrolidine-1-carbonyl chloride to give **78**, which underwent a palladium-catalyzed direct C-2 arylation with **77** to give **19**.

Compound **24** (Scheme 7) was prepared starting from 3-amino-5-morpholinopyridazine which was aminated on the 2-position to give diamino compound **79**. Alongside this, ethyl 5-amino-2-fluorobenzoate was treated with pyrrolidine-1-carbonyl chloride, with subsequent ester hydrolysis giving **80**, which was converted to acid chloride **81** by treatment with sulfonyl chloride. Compounds **79** and **81** were then cyclized to give **24**.

Compound **25** was synthesized according to Scheme 8, whereby 2-chloro-5-bromopyrimidine was treated with hydrazine to give **82**, which was condensed with 2-fluoro-5-nitrobenzaldehyde to give **83** and then cyclized to **84**. Thermal rearrangement led to **85**, with nitroreduction giving **86**. This was cross-coupled with morpholine and then treated with pyrrolidine-1-carbonyl chloride to give **25**.

To explore the SAR around the 3-position of the 6-phenylimidazo[1,2-*a*]pyrimidine scaffold, two approaches were taken; either an initial cyclization, followed by functionalization of the 3-position of the bi-cycle or cyclization of a precursor with the 3-substituent in place. The synthesis of **28** was previously described,^29^ and a similar route was used to synthesize the pyridyl analogue **29**, as shown in Scheme 9, where the previously reported 2-aminopyrimidine **87** was cyclized with 2-bromo-1,1-dimethoxyethane to give **88**, which could be brominated to **89**.^10^ The Suzuki coupling of **89** with 2-pyridineboronic acid then led to **29**. Alternatively, to access alkyl substitutions, **87** could be cyclized directly with a suitable α-bromoaldehyde to give **30** and **31**.

**Scheme 10:**
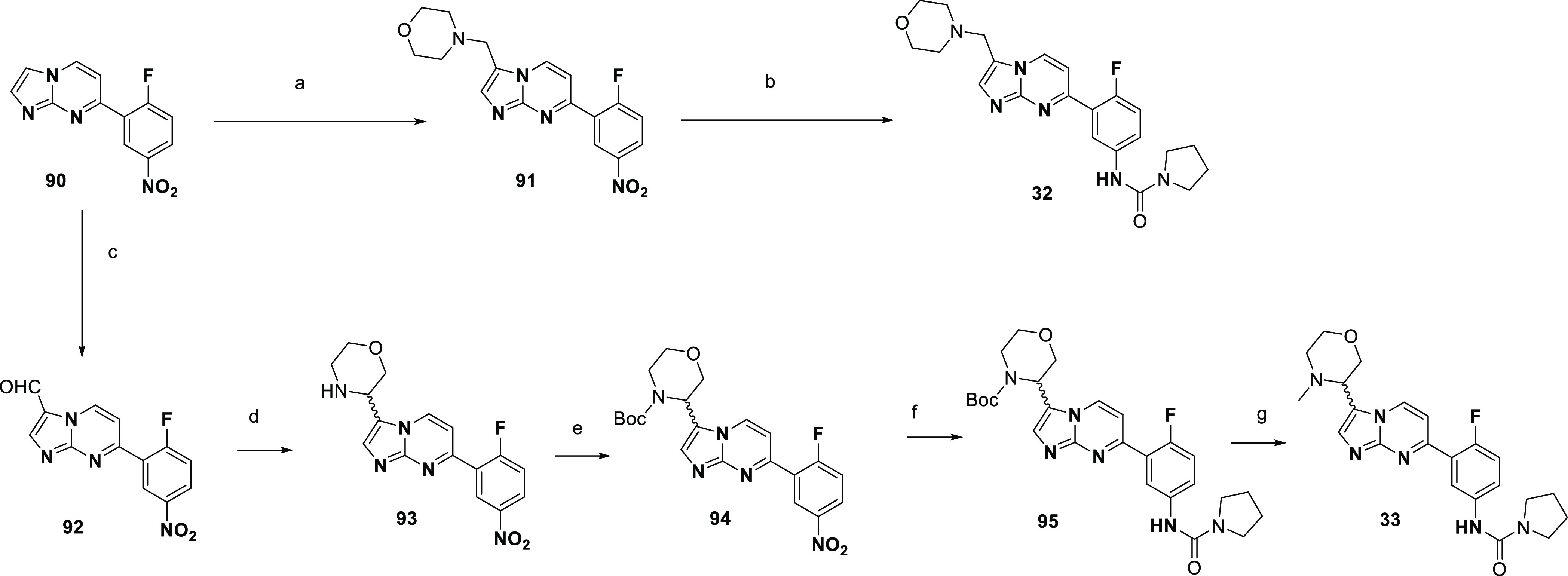
Synthesis of Compounds **32** and **33** Reagents and conditions: (a) paraformaldehyde, morpholine, acetic acid, 50 °C, 18 h; (b) (i) iron, NH_4_Cl, EtOH, water, 80 °C, 18 h; (ii) pyrrolidine-1-carbonyl chloride, DMAP, pyridine, DCM, 50 °C, 18 h, 7% over three steps; (c) POCl_3_, DMF, 80 °C, 20 h; (d) 2-[(tributylstannyl)methoxy]ethanamine (SnAP-M), DCM, then 2,6-lutidine, hexafluoro-2-propanol, Cu(OTf)_2_, 50 °C, 3 h, 50% over two steps; (e) Boc_2_O, MeOH, 50 °C, 3 h, 62%; (f) (i) iron, NH_4_Cl, EtOH, water, 75 °C, 3 h (ii) pyrrolidine-1-carbonyl chloride, DMAP, pyridine, DCM, 50 °C, 20 h, 36%; (g) (i) TFA DCM, RT, 1 h; (ii) paraformaldehyde, acetic acid, THF, RT, 4 h, then sodium triacetoxyborohydride, RT, 24 h, 28%.

Alternatively, to synthesize C-linked morpholine analogues **32** and **33**, the routes shown in Scheme 10 were utilized. Intermediate **90** could undergo a Mannich reaction to give **91**, with nitro reduction and subsequent urea formation giving **32**. A Vilsmeier–Haack reaction on **90** led to formylated **92**. Its treatment with a SnAP reagent^30^ led to the carbon-linked morpholine substituent **93**, which could be Boc-protected to give **94**, the nitro group reduced, and converted to pyrrolidinyl urea **95**, with Boc deprotection and subsequent methylation giving **33**.

**Scheme 11:**
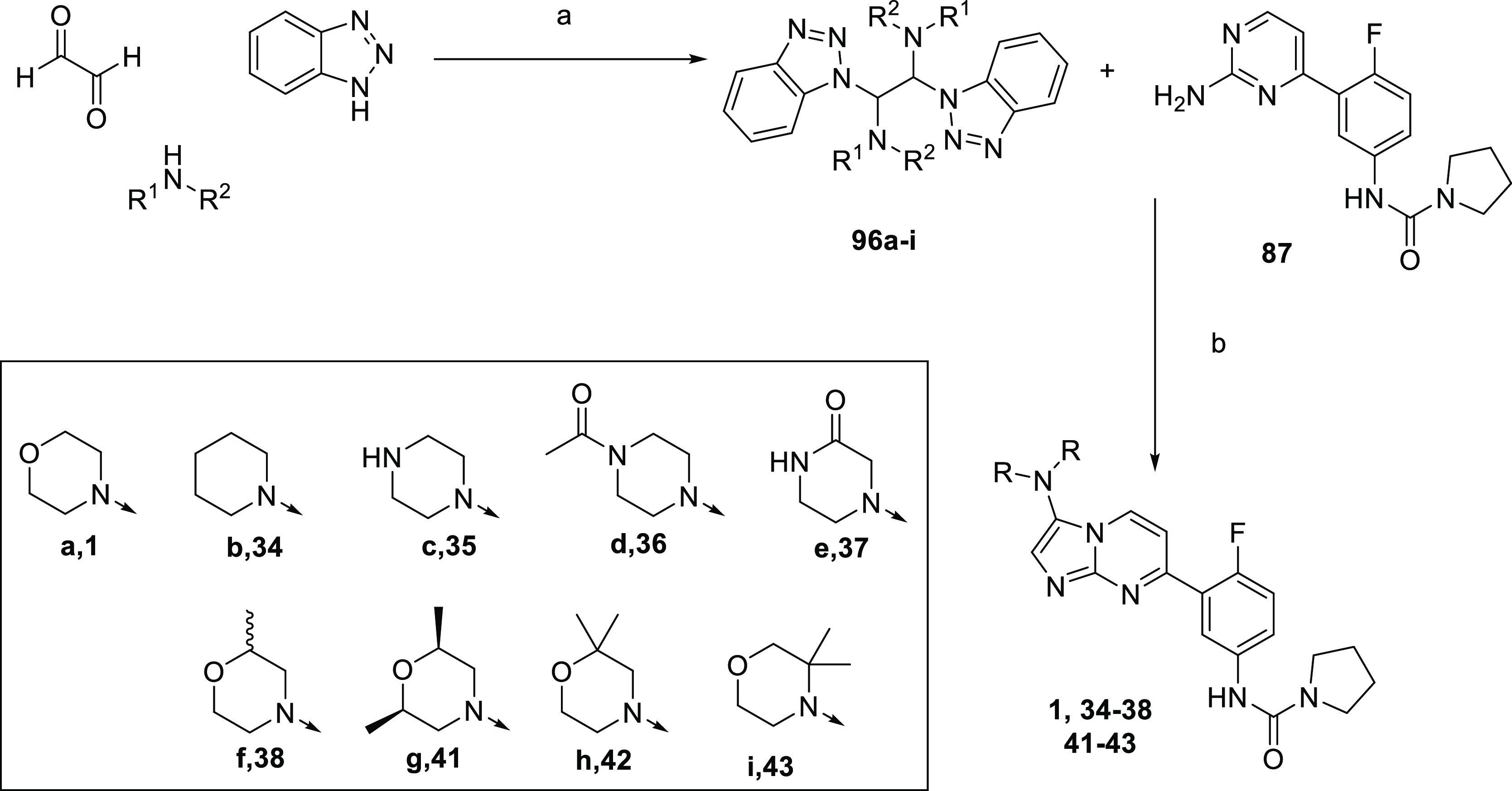
Synthesis of Compounds **1**, **34–38**, and **41–43** Reagents and conditions: (a) EtOH, RT, 12 h, 83%; (b) ZnBr_2_, DCM, reflux, 12 h, 5–46%.

**Scheme 12:**
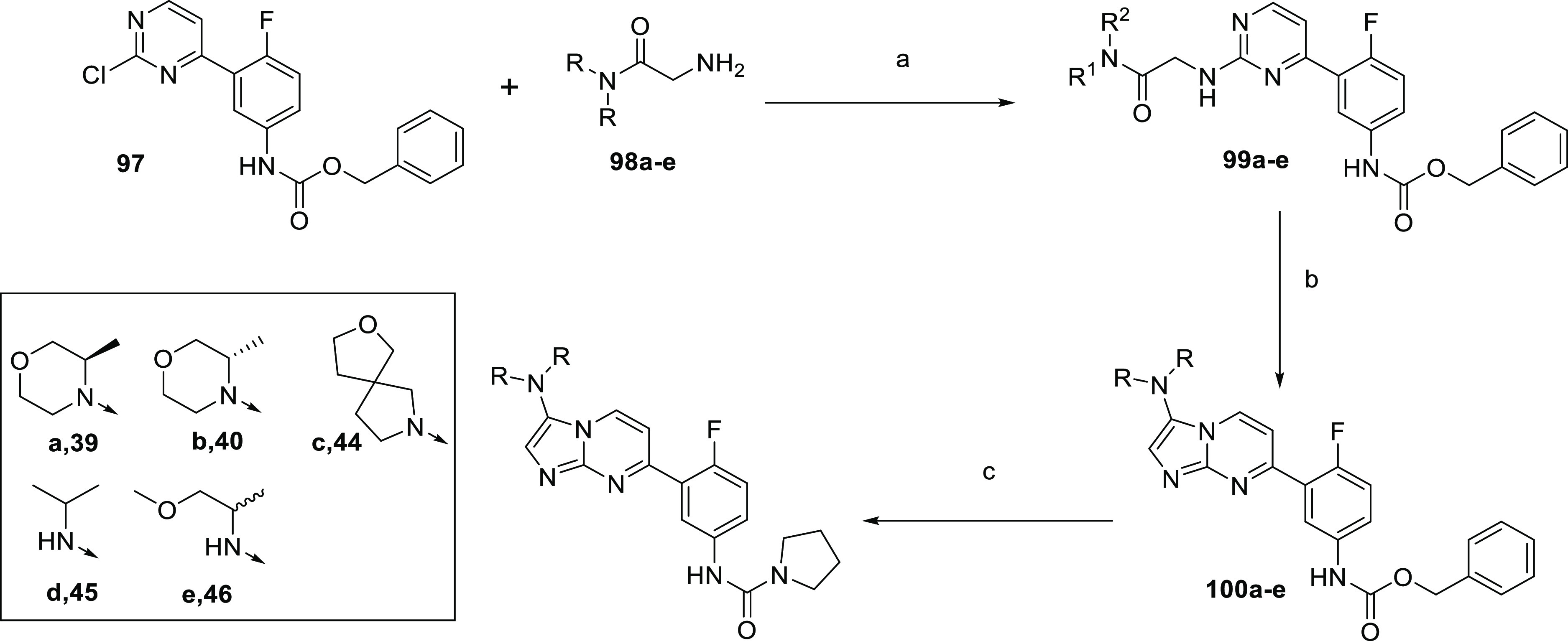
Synthesis of Compounds **39**, **40**, and **44–46** Reagents and conditions: (a) DIPEA, 1,4-dioxane, 120 °C, 18 h, 56%; (b) POCl_3_, 80 °C, 1 h, 67%; (c) (i) Pd/C, H_2_, MeOH, RT, 18 h; (ii) CDI, DIPEA, DCM, 18 h, then pyrrolidine, RT, 4.5 h, 22–46%.

The 3-amino analogues were synthesized according to Schemes 11 and 12. Where applicable, the previously published route for compound **1** was utilized (Scheme 11), whereby the relevant amine was condensed with glyoxal and benzotriazole to give 1,2-bis electrophiles **96a-i**, which were subsequently cyclized with **87** under Lewis acid-promoted conditions to give compounds **1**, **34–38**, and **41–43**. In cases where this was unsuccessful, due to the relevant **96** not forming, the alternative route^31^ in Scheme 12 was utilized, whereby 2-chloropyrimidine analogue **97** was treated with relevant glycinamide **98a–e** to give **99a–e** which was cyclized to **100a–e**. Cbz-Deprotection, followed by generation of the urea thus led to compounds **39**, **40**, and **44**−**46**.

## Conclusions

2-Phenylimidazo[1,2-*a*]pyrimidine **3** was a suitable starting point for a phenotypic lead-optimization program for VL. Changes to the central phenyl ring demonstrated that it was possible to significantly improve FaSSIF solubility but failed to yield compounds with a suitable balance of potency, metabolic stability, and FaSSIF solubility; we, therefore, embarked on a scaffold-hopping exercise. A round of design and synthesis led to a set of 14 compounds with different cores, giving us an understanding of the requirements for potency, where HBA’s at positions 1 and 8 of the bi-cycle were critical for activity. While the scaffolds, which fulfilled these requirements, notably **20–26** and the “reversed” scaffolds **1** and **27**, all met our target potency in the INMAC assay, the solubilities, in both aqueous and FaSSIF media, were highly variable in ways that were difficult to predict.

From this, compounds **1** and **23** were identified as the most promising for progression, although the development of **23** was halted due to a genotoxicity issue. Attempts were made to optimize the morpholine substituent in compound **1** with a variety of different analogues and replacements. While some of these showed greater potency, this came at the cost of solubility and/or microsomal stability. Compound **1** was selected for further profiling, showing that neither the parent, nor the aniline potentially released by the hydrolysis of the urea were positive in the Ames assay. After safety profiling, **1** was selected as a preclinical candidate for VL, as previously reported.^10^

Modeling studies were carried out using the cryoEM *L. tarentolae* proteasome structure that we had previously reported. This was used to generate a model of the *L. donovani* proteasome. The structure was not available until after the chemistry program had finished. By performing a pair interaction energy decomposition analysis (PIEDA) using the fragment molecular orbital method (FMO), we were able to predict which are the important interactions between the protein and ligand on a residue by residue basis. Important interactions were with Thr100, Gly122, Asp214, Asp215, Val227, and Gly228. The presence of a negative charge on the bottom edge of the bi-cycle (positions 1 and 8, Figure 2) was important for the interaction with Thr100 and positive charge on positions 3 and 4 for interaction with Asp214 and Asp215. The relative importance of the protein–ligand interactions would not have been identified unless such a sophisticated analysis had been carried out. We also analyzed the ESP of the ligands. When combining this with the FMO analysis, we were able to rationalize the SAR that we have seen. We suggest that this approach could be used going forward in predicting whether modifications to compounds are likely to improve binding.

This project demonstrates the utility of Cryo-EM co-structures for rationalizing protein–ligand interactions. In this case, we were able to obtain structures of sufficient resolution to be able to understand in a detailed manner the protein–ligand interactions. As well as being able to rationalize the SAR, as we have previously reported, we were able to use the structural information to rationalize the selectivity of our compounds for the parasite proteasome compared to the human proteasome, as reported previously.^10^

The proteasome represents an exciting new drug target for VL and our lead compound, **1**, is being progressed toward human studies.

## Experimental Section

### Chemistry

Chemicals and solvents were purchased from the Aldrich Chemical Company, Fluka, ABCR, VWR, Acros, Fluorochem, and Alfa Aesar and were used as received. Air- and moisture-sensitive reactions were carried out under an inert atmosphere of argon in oven-dried glassware. Analytical thin-layer chromatography (TLC) was performed on precoated TLC plates (layer 0.20 mm silica gel 60 with fluorescent indicator UV254, from Merck). Developed plates were air-dried and analyzed under a UV lamp (UV254/365 nm). Flash column chromatography was performed using prepacked silica gel cartridges (230–400 mesh, 40–63 mm, from SiliCycle) using a Teledyne ISCO CombiFlash Companion, or CombiFlash Retrieve. ^1^H NMR and ^13^C NMR spectra were recorded on a Bruker AVANCE DPX 500 spectrometer (^1^H at 500.1 MHz, ^13^C at 125.8 MHz). Chemical shifts (δ) are expressed in ppm recorded using the residual solvent as the internal reference in all cases. Signal splitting patterns are described as singlet (s), doublet (d), triplet (t), quartet (q), multiplet (m), broad (b), or a combination thereof. Coupling constants (J) are quoted to the nearest 0.1 Hz. LC–MS analyses were performed with either an Agilent HPLC 1100 series connected to a Bruker Daltonics MicrOTOF or an Agilent Technologies 1200 series HPLC connected to an Agilent Technologies 6130 quadrupole LC/MS, where both instruments were connected to an Agilent diode array detector. The mobile phase was water/acetonitrile + 0.1% HCOOH or water/acetonitrile + 0.1% NH_3_; linear gradient 80:20 to 5:95 over 3.5 min, and then held for 1.5 min; flow rate 0.5 mL min^–1^. All intermediates had a measured purity ≥90% and all assay compounds had a measured purity of ≥95% as determined using this analytical LC–MS system (TIC and UV). High-resolution electrospray measurements were performed on a Bruker Daltonics MicrOTOF mass spectrometer. Microwave-assisted chemistry was performed using a Biotage initiator microwave synthesizer. The synthesis of all intermediates, and spectral data for final compounds, is included in the Supporting Information.

### *N*-(2-Fluoro-5-(6-morpholinoimidazo[1,2-*a*]pyrimidin-2-yl)phenyl)pyrrolidine-1-carboxamide (**5**)

To a suspension of **50a** (0.32 g, 1.02 mmol) in pyridine (19 mL) was added 4-dimethylaminopyridine (DMAP) (0.0062 g, 0.051 mmol) and pyrrolidine-1-carbonyl chloride (0.169 mL, 1.532 mmol), and the resulting suspension stirred at RT for 40 h then at 40 °C for 24 h. Further pyrrolidine-1-carbonyl chloride (0.169 mL, 1.532 mmol) was added, and the resulting suspension stirred at 40 °C for 2 days. Further pyrrolidine-1-carbonyl chloride (0.113 mL, 1.021 mmol) was then added and the reaction mixture stirred at 40 °C for a further 24 h. The temperature was then increased to 55 °C and the mixture was stirred for another 48 h. The reaction mixture was then heated at 65 °C before adding more pyrrolidine-1-carbonyl chloride (0.205 g, 1.532 mmol). The resulting mixture was heated at 55 °C for 2 days. Solvents were evaporated, and the residue purified by flash chromatography (0–5% MeOH/DCM). Brown oil was obtained which was further purified by prep. HPLC to yield **5** as a yellow solid (0.05 g, 0.12 mmol, 12% yield). ^1^H NMR (DMSO-*d*_6_): δ 8.54 (d, *J* = 2.9 Hz, 1H), 8.30 (d, *J* = 3.0 Hz, 1H), 8.06 (d, *J* = 7.2 Hz, 2H), 7.82 (s, 1H), 7.58 (ddd, *J* = 8.5, 4.6, 2.3 Hz, 1H), 7.17 (dd, *J* = 10.6, 8.5 Hz, 1H), 3.77–3.65 (m, 4H), 3.36–3.28 (m, 4H), 3.05–2.98 (m, 4H), 1.86–1.69 (m, 4H); ^13^C NMR (DMSO-*d*_6_): δ 156.3, 154.4, 154.2, 147.0, 145.8, 136.7, 130.2, 128.4 (d, *J*_CF_ = 12.1 Hz), 123.3, 122.1 (d, *J*_CF_ = 7.5 Hz), 119.1, 116.2 (d, *J*_CF_ = 20.1 Hz), 107.8, 66.3, 50.0, 46.2, 25.5; HRMS (ES^+^): *m*/*z* [M + H]^+^ calcd for C_21_H_24_FN_6_O_2_, 411.1939; found, 411.1939.

### *N*-(3-(6-Morpholinoimidazo[1,2-*a*]pyrimidin-2-yl)phenyl)pyrrolidine-1-carboxamide (**6**)

A mixture of **49** (0.347 g, 1.92 mmol) and **52b** (0.6 g, 1.92 mmol) in dimethylformamide (DMF) (5 mL) was stirred at 90 °C for 18 h. After cooling, the solvent was evaporated and the crude material was purified by flash chromatography (0–5% MeOH/DCM) to give **6** (0.035 g, 0.085 mmol, 5%). ^1^H NMR (DMSO-*d*_6_): δ 8.64–8.62 (m, 1H), 8.42–8.40 (m, 1H), 8.25 (s, 1H), 8.14–8.13 (m, 1H), 8.11 (s, 1H), 7.56 (d, *J* = 8.0 Hz, 1H), 7.49 (d, *J* = 7.6 Hz, 1H), 7.29 (dd, *J* = 7.9 and 7.3 Hz, 1H), 3.81–3.78 (m, 4H), 3.42–3.38 (m, 4H), 3.12–3.09 (m, 4H), 1.89–1.85 (m, 4H); ^13^C NMR (DMSO-*d*_6_): δ 154.4, 146.8, 145.9, 145.6, 141.6, 136.6, 134.2, 129.1, 119.4, 119.2, 119.1, 117.0, 107.8, 66.4, 50.2, 46.2, 25.6; HRMS (ES^+^): *m*/*z* [M + H]^+^ calcd for C_21_H_25_N_6_O_2_, 393.2039; found, 393.2054.

### *N*-(4-Methoxy-3-(6-morpholinoimidazo[1,2-*a*]pyrimidin-2-yl)phenyl)pyrrolidine-1-carboxamide (**7**)

To a suspension of **50c** (0.158 g, 0.486 mmol) in pyridine (9.2 mL), DMAP (0.003 g, 0.024 mmol) and pyrrolidine-1-carbonyl chloride (0.080 mL, 0.728 mmol) were added, and the resulting suspension was stirred at RT for 40 h. Solvents were evaporated and the crude product was purified by flash chromatography (0–10% MeOH/DCM). Brown oil was obtained which was triturated with acetone and further purified by preparative HPLC to yield **7** (0.074 g, 0.17 mmol, 36% yield). ^1^H NMR (DMSO-*d*_6_): δ 8.53 (d, *J* = 2.9 Hz, 1H), 8.38 (d, *J* = 2.9 Hz, 1H), 8.23 (d, *J* = 2.8 Hz, 1H), 8.14 (s, 1H), 8.05 (s, 1H), 7.49 (dd, *J* = 8.9, 2.8 Hz, 1H), 6.93 (d, *J* = 9.0 Hz, 1H), 3.83 (s, 3H), 3.71 (dd, *J* = 5.9, 3.5 Hz, 4H), 3.34–3.26 (m, 4H), 3.04–2.97 (m, 4H), 1.81–1.74 (m, 4H); ^13^C NMR (DMSO-*d*_6_): δ 154.7, 152.2, 147.0, 144.9, 141.4, 136.3, 134.4, 121.9, 120.8, 120.5, 119.2, 111.8, 111.7, 66.3, 56.2, 50.1, 46.1, 25.6; HRMS (ES^+^): *m*/*z* [M + H]^+^ calcd for C_22_H_27_N_6_O_3_, 423.2139; found, 423.2138.

### *N*-(2-Methoxy-5-(6-morpholinoimidazo[1,2-*a*]pyrimidin-2-yl)phenyl)pyrrolidine-1-carboxamide (**8**)

To a suspension of **50d** (0.094 g, 0.289 mmol) in pyridine (5.45 mL) were added DMAP (1.8 mg, 0.014 mmol) and pyrrolidine-1-carbonyl chloride (0.048 mL, 0.433 mmol), and the resulting suspension was stirred at RT for 40 h. More pyrrolidine-1-carbonyl chloride (0.048 mL, 0.433 mmol) was added, and the resulting solution was stirred at RT over 3 days. Solvents were evaporated and the crude material was purified by flash chromatography (0–10% MeOH/DCM). The resulting yellow solid was dissolved in DCM/MeOH (5 mL) and washed with water (15 mL). The aqueous layer was further extracted with DCM (2 × 5 mL). The organic phases were combined, dried over anhydrous Na_2_SO_4_, filtered, and concentrated to give a brown pale solid, which was dried under vacuum to yield **8** (0.011 g, 0.03 mmol, 9%). ^1^H NMR (DMSO-*d*_6_): δ 8.50 (d, *J* = 2.9 Hz, 1H), 8.39 (d, *J* = 2.1 Hz, 1H), 8.29 (d, *J* = 2.9 Hz, 1H), 7.96 (s, 1H), 7.49 (dd, *J* = 8.4, 2.2 Hz, 1H), 7.12 (s, 1H), 6.99 (d, *J* = 8.6 Hz, 1H), 3.80 (s, 3H), 3.76–3.65 (m, 4H), 3.35–3.26 (m, 4H), 3.06–2.97 (m, 4H), 1.81 (t, *J* = 6.5 Hz, 4H); ^13^C NMR (DMSO-*d*_6_): δ 153.9, 149.3, 146.3, 145.9, 145.8, 136.5, 129.5, 126.6, 120.2, 119.0, 118.2, 111.3, 107.0, 66.3, 56.5, 50.1, 45.9, 25.6; HRMS (ES^+^): *m*/*z* [M + H]^+^ calcd for C_22_H_27_N_6_O_3_, 423.2145; found, 423.2139.

### *N*-(6-(6-Morpholinoimidazo[1,2-*a*]pyrimidin-2-yl)pyridin-2-yl)pyrrolidine-1-carboxamide (**9**)

**9** was synthesized by an analogous method to **6** from **49** (0.21 g, 1.17 mmol) and **50e** (0.364 g, 1.17 mmol) with purification done by flash chromatography (2–10% MeOH/DCM) to give **9** (0.1 g, 0.25 mmol, 22%). ^1^H NMR (DMSO-*d*_6_): δ 8.69–8.67 (m, 1H), 8.50–8.48 (m, 1H), 8.45 (s, 1H), 8.21 (s, 1H), 7.83–7.69 (m, 3H), 3.82–3.78 (m, 4H), 3.48–3.42 (m, 4H), 3.14–3.10 (m, 4H), 1.90 1.84 (m, 4H); ^13^C NMR (DMSO-*d*_6_): δ 153.8, 153.5, 151.2, 147.4, 145.7, 145.3, 138.8, 136.7, 119.2, 114.4, 112.9, 110.1, 66.3, 49.9, 46.2, 25.4; HRMS (ES^+^): *m*/*z* [M + H]^+^ calcd for C_20_H_24_N_7_O_2_, 394.1986; found, 394.1984.

### *N*-(6-(6-Morpholinoimidazo[1,2-*a*]pyrimidin-2-yl)pyrimidin-4-yl)pyrrolidine-1-carboxamide (**10**)

**10** was synthesized by an analogous method to **6** from **49** (0.306 g, 1.7 mmol) and **52f** (0.682 g, 1.7 mmol) with purification by flash chromatography (0.5–2% MeOH/DCM) to give **10** (0.322 g, 0.82 mmol, 48%). ^1^H NMR (DMSO-*d*_6_): δ 9.32 (s, 1H), 8.76–8.74 (m, 2H), 8.58 (s, 1H), 8.47–8.45 (m, 1H), 8.37 (s, 1H), 3.82–3.78 (m, 4H), 3.51–3.42 (m, 4H), 3.14–3.10 (m, 4H), 1.89–1.82 (m, 4H); ^13^C NMR (DMSO-*d*_6_): δ 160.6, 159.7, 158.4, 153.2, 148.6, 145.9, 143.7, 137.0, 66.3, 50.0, 46.4, 31.0; HRMS (ES^+^): *m*/*z* [M + H]^+^ calcd for C_19_H_23_N_8_O_2_, 395.1938; found, 395.1939.

### *N*-(6-(6-Morpholinoimidazo[1,2-*a*]pyrimidin-2-yl)pyrazin-2-yl)pyrrolidine-1-carboxamide (**11**)

**11** was synthesized by an analogous method to **6** from **49** (0.208 g, 1.16 mmol) and **52g** (0.452 g, 1.45 mmol), with purification by prep. HPLC to give **11** (0.071 g, 0.18 mmol, 12%). ^1^H NMR (DMSO-*d*_6_): δ 9.06–9.04 (m, 2H), 8.86 (s, 1H), 8.75–8.73 (m, 1H), 8.53–8.51 (m, 1H), 8.26 (s, 1H), 3.82–3.79 (m, 4H), 3.50–3.43 (m, 4H), 3.15–3.11 (m, 4H), 1.92–1.84 (m, 4H); ^13^C NMR (DMSO-*d*_6_): δ 153.5, 149.9, 148.2, 145.9, 145.7, 142.7, 136.9, 135.8, 134.6, 119.2, 111.1, 66.2, 49.8, 46.4, 30.5; HRMS (ES^+^): *m*/*z* [M + H]^+^ calcd for C_19_H_23_N_8_O_2_, 395.1938; found, 395.1946.

### *N*-(4-(6-Morpholinoimidazo[1,2-*a*]pyrimidin-2-yl)pyrimidin-2-yl)pyrrolidine-1-carboxamide (**12**)

**12** was synthesized by an analogous method to **6** from **49** (0.206 g, 1.14 mmol) and crude **52h** (1 equiv), with purification by flash chromatography (0.5–3% MeOH/DCM) giving **12** (0.293 g, 0.73 mmol). ^1^H NMR (DMSO-*d*_6_): δ 9.20 (s, 1H), 8.77–8.74 (m, 1H), 8.60 (d, *J* = 5.0 Hz, 1H), 8.53–8.50 (m, 1H), 8.33 (s, 1H), 7.60 (d, *J* = 5.0 Hz, 1H), 3.82–3.78 (m, 4H), 3.47–3.40 (m, 1H), 3.15–3.10 (m, 4H), 1.89–1.84 (m, 4H); ^13^C NMR (DMSO-*d*_6_): δ 160.6, 159.8, 159.4, 153.1, 148.6, 145.9, 143.1, 136.9, 119.1, 112.3, 110.3, 66.2, 49.7, 46.5, 25.6; HRMS (ES^+^): *m*/*z* [M + H]^+^ calcd for C_19_H_23_N_8_O_2_, 395.1938; found, 395.1954.

### *N*-(6-(6-Morpholinoimidazo[1,2-*a*]pyrimidin-2-yl)pyridazin-4-yl)pyrrolidine-1-carboxamide (**13**)

**13** was synthesized by an analogous method to **6** from **49** (0.208 g, 1.15 mmol) and **52i** (0.355 g, 1.43 mmol), with purification by flash chromatography (0–5% MeOH/DCM) giving **13** (0.013 g, 0.03 mmol, 2%). ^1^H NMR (DMSO-*d*_6_): δ 9.40–9.38 (m, 1H), 8.96 (s, 1H), 8.73–8.71 (m, 1H), 8.56–8.54 (m, 1H), 8.51–8.49 (m, 1H), 8.43 (s, 1H), 3.82–3.79 (m, 4H), 3.47–3.41 (m, 4H), 3.14–3.11 (m, 4H), 1.92–1.87 (m, 4H); ^13^C NMR (DMSO-*d*_6_): δ 155.2, 153.3, 147.9, 145.8, 142.8, 142.6, 141.0, 136.9, 119.2, 110.5, 109.9, 66.8, 49.9, 46.4, 25.3; HRMS (ES^+^): *m*/*z* [M + H]^+^ calcd for C_19_H_23_N_8_O_2_, 395.1944; found, 395.1961.

### *N*-[4-Fluoro-3-(7-morpholinoimidazo[1,2-*b*]pyridazin-2-yl)phenyl]pyrrolidine-1-carboxamide (**15**)

A solution of 5-morpholinopyridazin-3-amine^32^ (**56**, 0.12 g, 0.67 mmol) and **54** (0.241 g, 0.73 mmol) in MeCN (10 mL) was heated to 60 °C for 2 days. After cooling, the resulting precipitate was collected by filtration, loaded onto a column, and purified by flash chromatography (10% MeOH/EtOAc). Clean fractions were combined and concentrated, and the resulting yellow solid was triturated and collected by filtration to give **15** (0.121 g, 0.29 mmol, 43%). ^1^H NMR (DMSO-*d*_6_): δ 8.66 (d, *J* = 1.8 Hz, 1H), 8.36 (d, *J* = 4.4 Hz, 1H), 8.30 (s, 1H), 8.21 (d, *J* = 3.7 Hz, 1H), 7.56–7.54 (m, 1H), 7.20–7.14 (m, 2H), 3.80 (s, 4H), 3.39 (s, 4H), 3.30 (s, 4H), 1.87 (s, 4H); ^13^C NMR (DMSO-*d*_6_): δ 156.0, 154.5, 154.0, 143.0, 140.3, 138.9, 137.7 (d, *J*_CF_ = 19.9 Hz), 121.2 (d, *J*_CF_ = 13.5 Hz), 120.5 (d, *J*_CF_ = 7.9 Hz), 119.4, 115.7 (d, *J*_CF_ = 10.1 Hz), 113.8 (d, *J*_CF_ = 13.1 Hz), 102.4, 66.1, 48.1, 46.1, 25.5; [M + H]^+^ calcd for C_21_H_24_FN_6_O_2_, 411.1945; found 411.1948.

### *N*-(3-(6-Phenylpyrazolo[1,5-*a*]pyrimidin-2-yl)phenyl)pyrrolidine-1-carboxamide (**16**)

To a suspension of crude **61** (0.350 g, 1.11 mmol) in EtOH (20 mL) was added a solution of ammonium chloride (0.237 g, 4.43 mmol) in water (3 mL). The stirred mixture was heated to 75 °C and then treated in a single portion with finely divided iron (0.495 g, 8.85 mmol). The reaction mixture was stirred at this temperature for 2 h, cooled to RT, and filtered through a pad of celite, further eluting with MeOH. The resulting solution was concentrated under reduced pressure, and the residue partitioned between DCM and water. The aqueous layer was further extracted with DCM, and the combined organics were washed with brine, dried over MgSO_4_, and concentrated to give crude 3-(6-phenylpyrazolo[1,5-*a*]pyrimidin-2-yl)aniline (0.272 g, 0.95 mmol), which was used without purification. The crude material (0.250 g, 0.87 mmol) was taken up in pyridine (1 mL)/DCM (10 mL), treated dropwise with pyrrolidine-1-carbonyl chloride (0.175 g, 1.31 mmol), and stirred at 70 °C for 48 h. After cooling, the reaction was diluted with DCM (20 mL), washed with sat. NaHCO_3_, dried (MgSO_4_), and the solvent was evaporated. The resulting solid was triturated with EtOAc, collected by filtration, and chromatographed (100% EtOAc) to give **16** (0.234 g, 0.55 mmol, 62%). ^1^H NMR (DMSO-*d*_6_): δ 9.47 (s, 1H), 8.97–8.92 (m, 1H), 8.29 (d, *J* = 8.3 Hz, 2H), 7.88 (d, *J* = 7.6 Hz, 2H), 7.64–7.60 (m, 2H), 7.57–7.53 (m, 2H), 7.49–7.44 (m, 1H), 7.39–7.34 (m, 1H), 7.17 (s, 1H), 3.44–3.40 (m, 4H), 1.92–1.86 (m, 4H). ^13^C NMR (DMSO-*d*_6_): 156.4, 154.4, 149.9, 148.7, 141.7, 134.2, 132.9, 132.7, 129.7, 129.3, 128.7, 127.3, 121.9, 120.6, 120.2, 117.5, 93.3, 46.2, 25.5; *m*/*z* [M + H]^+^ calcd for C_23_H_22_N_5_O, 384.1836; found, 384.1824.

### *N*-(3-(5-Phenyl-1*H*-pyrrolo[2,3-*b*]pyridin-2-yl)phenyl)pyrrolidine-1-carboxamide (**17**)

To **63** (0.075 g, 0.20 mmol) in *N*-methyl-2-pyrrolidone (NMP) (1 mL) was added potassium *tert*-butoxide (0.066 g, 0.59 mmol) and stirred at 75 °C overnight. After cooling to RT, the RM was partitioned between satd. NH_4_Cl and DCM. The aqueous layer was further extracted with DCM, and the combined organics were washed with brine, dried over MgSO_4_, and the solvent was evaporated. The crude material was chromatographed (0–1% MeOH/EtOAc) to give **17** (0.045 g, 0.11 mmol, 57%). ^1^H NMR (CDCl_3_): δ 9.77 (br s, 1H), 8.57 (d, *J* = 2.1 Hz, 1H), 8.10 (d, *J* = 1.6 Hz, 1H), 8.01–7.99 (m, 1H), 7.69–7.66 (m, 2H), 7.53–7.49 (m, 2H), 7.45–7.39 (m, 4H), 6.86 (d, *J* = 2.1 Hz, 1H), 6.28 (s, 1H), 3.52–3.48 (m, 4H), 2.02–1.99 (m, 4H); ^13^C NMR (DMSO-*d*_6_): 154.4, 149.7, 142.2, 141.6, 140.2, 139.5, 132.1, 129.4, 129.3, 129.1, 127.3, 126.2, 121.5, 120.1, 119.3, 117.4, 97.5, 46.2, 25.5; *m*/*z* [M + H]^+^ calcd for C_24_H_23_N_4_O, 383.1872; found, 383.1882.

### *N*-(4-Fluoro-3-(2-morpholino-5*H*-pyrrolo[2,3-*b*]pyrazin-6-yl)phenyl)pyrrolidine-1-carboxamide (**18**)

To **68** (0.225 g, 0.56 mmol), morpholine (0.242 g, 2.78 mmol), tris(dibenzylideneacetone)dipalladium(0) (Pd_2_dba_3_) (0.0255 g, 0.028 mmol), and 2-dicyclohexylphosphino-2′,4′,6′-triisopropylbiphenyl (Xphos) (0.265 g, 0.56 mmol) in DMF (5 mL) was added sodium *tert*-butoxide (0.0053 g, 0.056 mmol), sealed, evacuated, flushed with N_2_, and stirred at 110 °C for 2 h. After cooling, RM was partitioned between water/EtOAc, and the organics was evaporated and chromatographed (0–6% MeOH/EtOAc). Fractions containing the product were combined, evaporated, and the resulting solid washed with MeOH and dried under vacuum to give **18** (0.063 g, 0.15 mmol, 26%). ^1^H NMR (DMSO-*d*_6_): δ 11.95 (s, 1H), 8.26 (s, 1H), 8.07 (s, 1H), 7.97 (dd, *J* = 2.7, 6.9 Hz, 1H), 7.50 (ddd, *J* = 2.7, 4.3 and 8.9 Hz, 1H), 7.24 (dd, *J* = 8.9, 10.8 Hz, 1H), 6.65 (dd, *J* = 2.2, 2.2 Hz, 1H), 3.77 (t, *J* = 4.8 Hz, 4H), 3.47 (t, *J* = 4.8 Hz, 4H), 3.41–3.37 (m, 4H), 1.88 (t, *J* = 6.6 Hz, 4H); ^13^C NMR (DMSO-*d*_6_): 155.9, 154.5, 153.9, 153.1, 137.7 (d, *J*_CF_ = 30.2 Hz), 136.2 (d, *J*_CF_ = 44.9 Hz), 126.6, 122.0 (d, *J*_CF_ = 8.1 Hz), 120.6, 119.6 (d, *J*_CF_ = 13.1 Hz), 116.4 (d, *J*_CF_ = 23.0 Hz), 100.1 (d, *J*_CF_ = 7.4 Hz), 66.5, 46.8, 46.1, 25.5; *m*/*z* [M + H]^+^ calcd for C_21_H_24_N_6_O_2_F, 411.1945; found, 411.1957.

### *N*-[4-Fluoro-3-(1-methyl-5-morpholino-pyrrolo[2,3-*b*]pyridin-2-yl)phenyl]pyrrolidine-1-carboxamide (**19**)

In a sealed vial, **78** (0.4614 g, 1.38 mmol), Pd(OAc)_2_ (0.0077 g, 0.03 mmol), 2-nitrobenzoic acid (0.1731 g, 1.04 mmol), and Ag_2_O (0.120 g, 0.52 mmol) were flushed with nitrogen. A solution of **77** (0.150 g, 0.69 mmol) in DMF (1 mL) was added, and the reaction mixture stirred at 90 °C overnight. The RM was allowed to cool to RT, filtered through a pad of celite, and eluted with DCM and then MeOH to give a brown solution. The solvent was evaporated, and the crude material was purified by flash chromatography (0–100% EtOAc/heptane then 0–10% MeOH/EtOAc). Fractions containing the product were combined and purified by prep. HPLC (20–95% MeCN) to afford **19** (0.005 g, 0.01 mmol, 2%). ^1^H NMR (CDCl_3_): δ 8.22 (d, *J* = 2.6 Hz, 1H), 7.53 (dd, *J* = 2.7, 6.3 Hz, 1H), 7.51–7.47 (m, 2H), 7.16–7.13 (m, 1H), 6.48 (s, 1H), 6.24 (br s, 1H), 3.96–3.93 (m, 4H), 3.79 (d, *J* = 1.5 Hz, 3H), 3.50–3.48 (m, 4H), 3.17–3.15 (m, 4H), 2.01–1.99 (m, 4H). ^13^C NMR (DMSO-*d*_6_): δ 155.7, 154.4, 153.8, 143.9 (d, *J*_CF_ = 175 Hz), 137.7 (d, *J*_CF_ = 2.5 Hz), 136.3, 136.1, 122.7, 122.0 (d, *J*_CF_ = 7.7 Hz), 119.9 (d, *J*_CF_ = 15.9 Hz), 116.1 (d, *J*_CF_ = 22.9 Hz), 115.3, 100.5, 66.7, 51.3, 46.2, 29.7, 25.5; HRMS (*m*/*z*): [M + H]^+^ calcd for C_23_H_27_FN_5_O_2_, 424.2143; found 424.2167.

### *N*-(4-Fluoro-3-(5-morpholinofuro[2,3-*b*]pyridin-2-yl)phenyl)pyrrolidine-1-carboxamide (**20**)

To a degassed suspension of **69** (0.462 g, 1.14 mmol) in 1,4-dioxane (11 mL) were added morpholine (300 μl, 3.43 mmol) and potassium bis(trimethylsilyl)amide (KHMDS) (4.6 ml, 2.29 mmol, 0.5 M in toluene). The resulting mixture was purged with nitrogen and then 2-dicyclohexylphosphino-2′,6′-diisopropoxybiphenyl (RuPhos) (0.107 g, 0.23 mmol) and (Pd2dba3) (0.105 g, 0.11 mmol) were added. The RM was stirred at 90 °C for 16 h, cooled, filtered through celite, and washed with DCM. The solvent was evaporated, and the crude material was purified by flash chromatography (0–40% EtOH/EtOAc (1:3)/cyclohexane) to give the crude product, which was further purified by flash chromatography (0–2% MeOH/DCM) to give **20** (0.070 g, 0.17 mmol, 15%). ^1^H NMR (CDCl_3_): δ 8.08–8.04 (m, 1H), 7.87–7.80 (m, 1H), 7.75–7.71 (m, 1H), 7.46 (s, 1H), 7.16–7.09 (m, 2H), 6.31 (s, 1H), 3.95–3.88 (m, 4H), 3.54–3.45 (m, 4H), 3.23–3.14 (m, 4H), 2.03–1.96 (m, 4H); *m*/*z* 410.6 [M + H]^+^; ^13^C NMR (DMSO-*d*_6_): δ 156.4, 155.5, 154.4, 153.6, 150.0 (d, *J*_CF_ = 3.8 Hz), 145.9, 138.1, 135.5, 121.9 (d, *J*_CF_ = 7.9 Hz), 121.2, 117.7, 117.1, 116.6 (d, *J*_CF_ = 22.3 Hz), 105.9 (d, *J*_CF_ = 12.7 Hz), 66.6, 50.3, 46.2, 25.5; *m*/*z* [M + H]^+^ calcd for C_22_H_24_N_4_O_3_F, 411.1832; found, 411.1840.

### *N*-[4-Fluoro-3-(2-morpholinofuro[2,3-*b*]pyrazin-6-yl)phenyl]pyrrolidine-1-carboxamide (**21**)

Pd_2_dba_3_ (0.0013 g, 0.0014 mmol), sodium *tert*-butoxide (0.027 g, 0.28 mmol), and Xphos (0.002 g, 0.0042 mmol) in a sealed vial were purged with nitrogen, and a suspension of **71** (0.05 g, 0.14 mmol) in DMF (1 mL) was added, followed by morpholine (0.06 g, 0.69 mmol). The RM was stirred at 100 °C for 1 h, cooled to RT and partitioned between water and EtOAc. The aqueous layer was further extracted with EtOAc, and the combined organics washed with brine, dried over MgSO_4_, and concentrated. The crude mixture was purified by flash chromatography (0–5% EtOAc/heptane) and fractions containing the product were combined and the solvent was removed. The residue was further purified by prep. HPLC (20–95% MeCN, 0.1% NH_4_OH) to give **21** (0.02 g, 0.046 mmol, 33%). ^1^H NMR (CDCl_3_): δ 7.84–7.81 (m, 3H), 7.26 (d, *J* = 3.2 Hz, 1H), 7.16 (dd, *J* = 9.0, 10.7 Hz, 1H), 6.29 (s, 1H), 3.93–3.90 (m, 4H), 3.61–3.58 (m, 4H), 3.52 (dd, *J* = 6.6, 6.6 Hz, 4H), 2.05–2.02 (m, 4H); ^13^C NMR (DMSO-*d*_6_): δ 155.6, 155.2, 154.3, 153.6, 153.0 (d, *J*_C–F_ = 3.8 Hz), 149.8, 138.2, 137.8, 126.2, 122.4 (d, *J*_C–F_ = 7.8 Hz), 117.2, 116.7 (d, *J*_C–F_ = 20.1 Hz), 106.0 (d, *J*_C–F_ = 15.0 Hz), 66.3, 46.2, 46.1, 25.5; *m*/*z* [M + H]^+^ calcd for C_21_H_23_N_5_O_3_F, 412.1785; found, 412.1797.

### *N*-(4-Fluoro-3-(3-morpholinofuro[2,3-c]pyridazin-6-yl)phenyl)pyrrolidine-1-carboxamide (**22**)

To a stirred solution of 4-bromo-1,2-dihydropyridazine-3,6-dione (3 g, 15.7 mmol) in pyridine (27 mL) under Ar at 5 °C was added triflic anhydride (2.64 mmol, 15.7 mmol) and stirred for 2h. The solvent was evaporated and RM was diluted with EtOAc, washed with sat. aqueous NaHCO_3_ and brine, and the combined organic layers was dried over Na_2_SO_4_, filtered, and concentrated to give **64** (3.7 g, 11.49 mmol, 73%), which was used without purification.

A suspension of **64** (0.590 g, 1.82 mmol), PdCl_2_(PPh_3_)_2_ (0.038 g, 0.05 mmol), copper (I) iodide (0.034 g, 0.18 mmol), and 62 (0.506 g, 2.37 mmol) in a mixture of MeCN/NEt_3_ (3 mL, 10:1) was stirred at RT for 6 h. DCM was added and the mixture was washed with water. The organic phase was dried and evaporated to obtain crude **65** which was used in the next step without further purification (0.892 g, 1.9 mmol). **65** was dissolved in dry morpholine (30 mL) and stirred at 50 °C overnight. After cooling, EtOAc was added followed by water. The organic phase was separated, the solvent was evaporated, and the crude material purified by flash chromatography (EtOAc/cyclohexane 0–100%). The resulting product was further purified by a SCX column, eluting with 2 M ammonia in MeOH. A second purification by SCX column, eluting with MeOH and then with 2 M ammonia in MeOH yielded **22** (0.093 g, 0.24 mmol, 12%). ^1^H NMR (DMSO-*d*_6_): δ 8.40 (s, 1H), 8.24–8.22 (m, 1H), 7.76–7.73 (m, 1H), 7.63–7.61 (m, 1H), 7.52 (s, 1H), 7.44–7.40 (m, 1H), 7.31 (s, 1H), 3.79–3.76 (m, 4H), 3.54–3.50 (m, 4H), 3.42–3.39 (m, 4H), 1.90–1.87 (m, 4H); ^13^C NMR (DMSO-*d*_6_): δ 162.1, 160.4, 159.7, 154.2, 142.0, 129.8, 128.5, 127.9, 121.8, 119.7, 116.6, 106.0, 100.2, 66.4, 46.9, 46.2, 25.5; *m*/*z* [M + H]^+^ calcd for C_21_H_24_N_5_O_3_, 394.1879; found, 394.1895.

### *N*-(4-Fluoro-3-(2-morpholinoimidazo[1,2-b][1,2,4]triazin-6-yl)phenyl)pyrrolidine-1-carboxamide (**23**)

Previously described in ref (10).

### *N*-(4-Fluoro-3-(7-morpholino-[1,2,4]triazolo[1,5-*b*]pyridazin-2-yl)phenyl)pyrrolidine-1-carboxamide (**24**)

To a mixture of **80** (0.2 g, 0.80 mmol) in DCM (5 mL) was added thionyl chloride (0.174 mL, 2.379 mmol) and stirred at 50 °C for 1 h. The solvent was evaporated, further DCM was added, and evaporated (2 × 10 mL) to give crude **81,** which was used without purification. To **79** and *N*,*N*-diisopropylethylamine (DIPEA) (0.174 mL, 0.994 mmol) in MeCN (3 mL) was added crude **81** (0.13 g, 0.66 mmol), and the mixture stirred at 80 °C for 24 h. After cooling, the mixture was filtered to remove the solid, and the filtrate concentrated and purified by flash chromatography (0–40% EtOH/EtOAc(1:3)/cyclohexane) to give **24** (0.035 g, 0.17 mmol, 13%). ^1^H NMR (DMSO-*d*_6_): δ 8.83–8.71 (m, 1H), 8.42–8.30 (m, 2H), 7.79–7.62 (m, 1H), 7.46–7.35 (m, 1H), 7.30–7.16 (m, 1H), 3.86–3.72 (m, 4H), 3.48–3.30 (m, 8H), 1.93–1.80 (m, 4H); ^13^C NMR (DMSO-*d*_6_): δ 159.0 (d, *J*_CF_ = 5.3 Hz), 156.4, 154.5, 146.7, 145.7, 137.7, 137.6 (d, *J*_CF_ = 2.7 Hz), 123.0 (d, *J*_CF_ = 7.7 Hz), 121.4, 118.5 (d, *J*_CF_ = 12.0 Hz), 116.7 (d, *J*_CF_ = 22.5 Hz), 110.9, 66.0, 47.4, 46.1, 25.5; *m*/*z* 412.1 [M + H]^+^. *m*/*z* [M + H]^+^ calcd for C_20_H_23_N_7_O_2_F, 412.1897; found, 412.1901.

### *N*-(4-Fluoro-3-(6-morpholino-[1,2,4]triazolo[1,5-*a*]pyrimidin-2-yl)phenyl)pyrrolidine-1-carboxamide (**25**)

A mixture of **85** (9 g, 29.2 mmol) and morpholine (115 mL, 1314 mmol) was stirred at 100 °C for 1 h. The mixture was concentrated to dryness and the crude solid washed with MeOH (2 × 50 mL), collected, and dried to give crude 4-fluoro-3-(6-morpholino-[1,2,4]triazolo[1,5-*a*]pyrimidin-2-yl)aniline (**86**, 6.4 g, 20.4 mmol, 70% crude yield), which was used without further purification. ^1^H NMR (DMSO-*d*_6_): δ 8.98 (d, *J* = 3.0 Hz, 1H), 8.90 (d, *J* = 2.8 Hz, 1H), 7.37 (dd, *J* = 6.2 and 2.9 Hz, 1H), 7.02 (dd, *J* = 10.9 and 8.8 Hz, 1H), 6.68 (dt, *J* = 8.5 and 3.6 Hz, 1H), 5.18 (s, 2H), 3.84–3.73 (m, 4H), 3.27–3.15 (m, 4H). To crude **86** (1.5 g, 4.77 mmol) and DMAP (0.029 g, 0.239 mmol) in pyridine (40 mL) was added pyrrolidine-1-carbonyl chloride (0.790 mL, 7.16 mmol), and the solution was stirred at 50 °C overnight. The resulting suspension was concentrated to dryness, and the crude residue was purified by flash chromatography (0–100% EtOH/EtOAc (1:3)/cyclohexane). The fractions containing the product were evaporated, EtOAc (100 mL) was added, and washed with water (250 mL)/HCl (2 M, 20 mL). The phases were separated and the organic layer was dried over Na_2_SO_4_, filtered, and concentrated to give **25** (0.57 g, 1.4 mmol, 29%). ^1^H NMR (DMSO-*d*_6_): δ 9.01 (d, *J* = 2.8 Hz, 1H), 8.92 (d, *J* = 2.8 Hz, 1H), 8.43–8.31 (m, 2H), 7.77–7.67 (m, 1H), 7.29–7.20 (m, 1H), 3.87–3.74 (m, 4H), 3.44–3.35 (m, 4H), 3.26–3.18 (m, 4H), 1.91–1.82 (m, 4H); ^13^C NMR (DMSO-*d*_6_): δ 161.1 (d, *J*_CF_ = 5.3 Hz), 156.3, 154.4, 154.3, 151.4, 150.8, 138.6, (d, *J*_CF_ = 2.8 Hz), 123.0 (d, *J*_CF_ = 18.7 Hz), 121.3 (d, *J*_CF_ = 4.8 Hz), 118.4 (d, *J*_CF_ = 12.0 Hz), 116.7 (d, *J*_CF_ = 22.4 Hz), 66.2, 49.5, 46.2, 25.5; *m*/*z* [M + H]^+^ calcd for C_20_H_23_N_7_O_3_F, 412.1897; found, 412.1903.

### *N*-(4-Fluoro-3-(5-morpholinopyrazolo[1,5-b]pyridazin-2-yl)phenyl)pyrrolidine-1-carboxamide (**26**)

**74** (0.035 g, 0.08 mmol) in HBr (2 mL, 48% aqueous) was heated in a microwave (120 °C, 1 h). The solvent was evaporated, water was added, and the solution was neutralized by the addition of sat. NaHCO_3_. The resulting solid was collected, washed with water, and dried to give crude **75** which was used directly in the next step (0.028 g, 0.08 mmol, 97%, *m*/*z* [M + H]^+^ 344.1). To crude **75** in EtOH (4 mL) was added iron (0.0325 g, 0.58 mmol) and ammonium chloride (0.0156 g, 0.29 mmol, in water (1 mL)) and stirred at 80 °C for 4 h. RM was filtered through celite, the solvent was evaporated and partitioned between water/EtOAc, and the organics was evaporated to dryness. This crude material was taken up in DCM (4 mL)/Pyridine (1 mL), and DMAP (0.001 g, 0.007 mmol) and pyrrolidine-1-carbonyl chloride (0.0146 g, 0.11 mmol) added. After stirring overnight at 50 °C, further pyrrolidine-1-carbonyl chloride (0.0146 g, 0.11 mmol) was added and stirred for a further night at 50 °C. After cooling, further DCM (10 ml) was added and washed with water, 1 M HCl, brine and the solvent evaporated. Crude material was purified by prep. HPLC to give **26** (0.008 g, 0.018 mmol, 24% over three steps). ^1^H NMR (DMSO-*d*_6_): δ 8.56 (d, *J* = 3.0 Hz, 1H), 8.31 (s, 1H), 8.24 (dd, *J* = 2.9, 6.8 Hz, 1H), 7.66 (ddd, *J* = 2.8, 4.4, 9.0 Hz, 1H), 7.33 (d, *J* = 3.1 Hz, 1H), 7.23–7.17 (m, 1H), 6.76 (d, *J* = 4.0 Hz, 1H), 3.84–3.77 (m, 4H), 3.41–3.36 (m, 4H), 3.27 (t, *J* = 4.8 Hz, 4H), 1.89–1.84 (m, 4H); ^13^C NMR (DMSO-*d*_6_): δ 156.7, 154.4, 154.3, 146.0, 141.0, 137.9, 137.7, 136.1, 121.3 (d, *J*_CF_ = 7.7 Hz), 120.2 (d, *J*_CF_ = 12.3 Hz), 119.5, 116.3 (d, *J*_CF_ = 22.9 Hz), 104.8, 95.0 (d, *J*_CF_ = 11.2 Hz), 66.1, 48.1, 46.2, 25.5; *m*/*z* [M + H]^+^ calcd for C_21_H_24_N_6_O_2_F, 411.1945; found, 411.1955.

### *N*-(3-(7-Phenylimidazo[1,2-*b*][1,2,4]triazin-3-yl)phenyl)pyrrolidine-1-carboxamide (**27**)

A mixture of **59** (0.030 g, 0.074 mmol), 4,4,5,5-tetramethyl-2-phenyl-1,3,2-dioxaborolane (0.030 g, 0.15 mmol), sodium carbonate (0.023 g, 0.22 mmol), and tetrakis(triphenylphosphine)palladium(0) (0.0011 g, 0.0015 mmol) in DMF (1.5 mL)/water (0.5000 mL) was heated to 80 °C in a sealed tube overnight. After cooling, water (10 mL) and EtOAc (10 mL) were added, the layers were separated, and the organic layer was evaporated. The crude product was purified by prep. HPLC to yield **27** (0.012 g, 0.028 mmol, 38%). ^1^H NMR (DMSO-*d*_6_): δ 9.16 (s, 1H), 8.61 (s, 1H), 8.49 (s, 1H), 8.35–8.31 (m, 1H), 8.20 (d, *J* = 7.5 Hz, 2H), 7.89–7.84 (m, 1H), 7.61–7.55 (m, 2H), 7.48–7.43 (m, 1H), 7.37–7.32 (m, 1H), 3.42–3.38 (m, 4H), 1.91–1.84 (m, 4H); *m*/*z* 403.2 [M + H]^+^.

### *N*-(4-Fluoro-3-(3-phenylimidazo[1,2-*a*]pyrimidin-7-yl)phenyl)pyrrolidine-1-carboxamide (**28**)

As described in ref (29).

### *N*-(4-Fluoro-3-(3-(pyridin-2-yl)imidazo[1,2-*a*]pyrimidin-7-yl)phenyl)pyrrolidine-1-carboxamide (**29**)

A mixture of **89** (0.19 g, 0.47 mmol), 2-pyridineboronic acid (0.195 g, 0.94 mmol), Pd(dppf)Cl_2_·CH_2_Cl_2_ (0.077 g, 0.094 mmol), and potassium carbonate (0.195 g, 1.41 mmol) in 1,4-dioxane (30 mL)/water (2 mL) was stirred under nitrogen at 100 °C for 10 h. The mixture was cooled to RT, evaporated, diluted with water (20 mL), and extracted with EtOAc (5 × 30 mL). The combined organic layers were dried over Na_2_SO_4_, filtered, and the solvent was evaporated. The crude material was purified by flash chromatography (1–5% MeOH/DCM) to give **29** as a yellow solid (0.02 g, 0.048 mmol, 10%). ^1^H NMR (MeOD): δ 10.42 (d, *J* = 7.5 Hz, 1H), 8.91 (br s, 1H), 8.70 (d, *J* = 4.6 Hz, 1H), 8.32–8.27 (m, 1H), 7.89–7.76 (m, 3H), 7.43–7.38 (m, 1H), 7.33 (dd, *J* = 5.4, 6.7 Hz, 1H), 7.11 (dd, *J* = 11.2, 9.3 Hz, 1H), 3.30–3.26 (m, 4H), 1.94–1.89 (m, 4H). *m*/*z* 403.2 [M + H]^+^.

### *N*-(4-Fluoro-3-(3-isopropylimidazo[1,2-*a*]pyrimidin-7-yl)phenyl)pyrrolidine-1-carboxamide (**30**)

A mixture of **87** (0.15 g, 0.5 mmol) and 2-bromo-3-methylbutanal (0.123 g, 0.75 mmol) in EtOH (2 mL) was heated to reflux for 4 h. The reaction mixture was cooled to RT, and the solvent was evaporated. The crude material was purified by prep. HPLC to obtain **30**, which was then diluted in DCM (5 mL) and treated dropwise with 2 N HCl in Et_2_O (0.191 mL, 0.381 mmol). The resulting solution was stirred at RT for 2 h, the solvent was evaporated, and the resulting solid was dried at 50 °C to give **30·HCl** (0.12 g, 0.297 mmol, 59%). ^1^H NMR (DMSO-*d*_6_): δ 9.39 (d, *J* = 7.2 Hz, 1H), 8.51 (s, 1H), 8.42 (dd, *J* = 6.8 and 2.8 Hz, 1H), 8.19 (s, 1H), 7.97 (dd, *J* = 7.2 and 1.6 Hz, 1H), 7.75 (ddd, *J* = 8.8, 7.2 and 2.8 Hz, 1H), 7.37 (dd, *J* = 11.2 and 8.8 Hz, 1H), 3.51–3.41 (m, 1H), 3.39 (t, *J* = 6.8 Hz, 4H), 1.87 (t, *J* = 6.8 Hz, 4H), 1.37 (d, *J* = 7.2 Hz, 6H). ^13^C NMR (DMSO-*d*_6_): δ 158.8, 155.2, 154.3, 144.0, 138.4, 136.5, 131.9, 125.3 (*J*_CF_ = 7.3 Hz), 123.3 (*J*_CF_ = 10.9 Hz), 121.2, 117.2 (*J*_CF_ = 24.4 Hz), 113.4 (*J*_CF_ = 7.8 Hz), 101.4, 46.2, 25.9, 23.4, 21.0; *m*/*z* 368.1 [M + H]^+^. HRMS (ES^+^): *m*/*z* [M + H]^+^ calcd for C_20_H_23_FN_5_O, 368.1887; found, 368.1899.

### *N*-(4-Fluoro-3-(3-isobutylimidazo[1,2-*a*]pyrimidin-7-yl)phenyl)pyrrolidine-1-carboxamide (**31**)

A mixture of **87** (0.15 g, 0.5 mmol) and 2-bromo-4-methylpentanal (0.18 g, 1 mmol) in EtOH was heated to reflux for 24 h. The reaction mixture was cooled to RT and partitioned between 10% aq NaHCO_3_ and EtOAc. The organic layer was dried over anhydrous Na_2_SO_4_, filtered, and the solvent evaporated. The crude material was purified by prep. HPLC to afford **31** (0.047 g, 0.123 mmol, 25%). ^1^H NMR (DMSO-*d*_6_): δ 9.36 (d, *J* = 7.2 Hz, 1H), 8.48 (s, 1H), 8.42 (dd, *J* = 6.8 and 2.4 Hz, 1H), 8.15 (s, 1H), 7.93 (d, *J* = 6.4 Hz, 1H), 7.73 (ddd, *J* = 8.8, 7.6 and 2.8 Hz, 1H), 7.37 (dd, *J* = 11.2 and 9.2 Hz, 1H), 3.38 (t, *J* = 6.8 Hz, 4H), 2.88 (d, *J* = 6.8 Hz, 2H), 2.11–2.01 (m, 1H), 1.87 (t, *J* = 6.4 Hz, 4H), 0.99 (d, *J* = 6.8 Hz, 6H); ^13^C NMR (DMSO-*d*_6_): δ 158.4 (d, *J*_CF_ = 33.6 Hz), 157.0, 155.0, 154.3, 138.4, 136.1, 125.1, 125.0, 123.6, 121.2, 117.2 (d, *J*_CF_ = 24.4 Hz), 115.7, 113.1, 46.2, 31.5, 26.9, 25.5, 22.5; HRMS (ES^+^): *m*/*z* [M + H]^+^ calcd for C_21_H_25_FN_5_O_2_, 382.2038; found, 382.2040.

### *N*-(4-Fluoro-3-(3-(morpholinomethyl)imidazo[1,2-*a*]pyrimidin-7-yl)phenyl)pyrrolidine-1-carboxamide (**32**)

A mixture of **90** (0.774 g, 3 mmol), paraformaldehyde (0.087 g, 3 mmol), and morpholine (0.261 g, 3 mmol) in glacial acetic acid (10 mL) was stirred at 50 °C overnight. After cooling, the RM was basified with 2 N NaOH to pH approx. 8 and extracted with DCM (3 × 30 mL). The combined organics were washed with brine (2 × 50 mL), dried over Na_2_SO_4_, filtered, and the solvent was evaporated to give crude **91**. This was taken up in EtOH (15 mL) to which iron (0.753 g, 13.4 mmol), and ammonium chloride (0.758 g, 6.7 mmol, in 4 mL water) were added, and the RM was heated to 80 °C for 1 h, cooled to RT, filtered through celite, and the solvent was evaporated. Water was added and extracted with EtOAc (3 × 50 mL), and the combined organics were dried over anh. Na_2_SO_4_, filtered, and concentrated to give a crude residue. This was taken up in pyridine (2 mL)/DCM (10 mL), DMAP (0.0056 g, 0.046 mmol), and pyrrolidine-1-carbonyl chloride (0.122 g, 0.92 mmol), and the RM was heated at 50 °C overnight. The solvent was evaporated and the crude material was purified by prep. HPLC to give **32** (0.083 g, 0.196 mmol, 7% over three steps). ^1^H NMR (CDCl_3_): δ 8.80 (d, *J* = 7.2 Hz, 1H), 8.15–7.74 (m, 3H), 7.59 (d, *J* = 6.8 Hz, 1H), 7.12 (dd, *J* = 10.8 and 9.2 Hz, 1H), 6.71 (broad s, 1H), 3.88 (br s, 2H), 3.74–3.67 (m, 4H), 3.53–3.46 (m, 4H), 2.55–2.44 (m, 4H), 2.05–1.92 (m, 4H); ^13^C NMR (DMSO-*d*_6_): δ 154.8, 154.4, 152.5, 148.8, 138.1, 135.8, 134.4, 125.2 (d, *J* = 11.7 Hz), 123.3 (d, *J* = 8.0 Hz), 121.3, 119.7, 116.7 (d, *J* = 23.8 Hz), 109.0 (d, *J* = 10.9 Hz), 66.6, 53.3, 51.6, 46.2, 25.5. HRMS (ES^+^): *m*/*z* [M + H]^+^ calcd for C_22_H_26_FN_6_O_2_, 425.2096; found, 425.2104.

### *N*-(4-Fluoro-3-(3-(4-methylmorpholin-3-yl)imidazo[1,2-*a*]pyrimidin-7-yl)phenyl)pyrrolidine-1-carboxamide (**33**)

To a solution of **95** (0.08 g, 0.16 mmol) in DCM (1 mL), trifluoroacetic acid (0.5 mL, 6.53 mmol) was added, and the resulting mixture stirred for 1 h and evaporated to dryness. To the crude compound was added acetic acid (0.6 mL, 10.5 mmol) and paraformaldehyde (0.045 g, 1.5 mmol) in tetrahydrofuran (THF) (3 mL), and the mixture stirred at RT for 4 h. Sodium triacetoxyborohydride (0.127 g, 0.6 mmol) was added, and the RM was stirred at RT for 24 h, evaporated to dryness, and purified by prep. HPLC to afford **33** (0.018 g, 0.042 mmol, 28%). ^1^H NMR (DMSO-*d*_6_): δ 9.29 (d, *J* = 7.3 Hz, 1H), 8.43 (s, 1H), 8.24–8.22 (m, 1H), 7.80–7.77 (m, 2H), 7.47 (d, *J* = 7.3 and 2.2 Hz, 1H), 7.27 (dd, *J* = 11.4 and 8.9 Hz, 1H), 3.88–3.85 (m, 1H), 3.77–3.69 (m, 4H), 3.41–3.37 (m, 4H), 2.91–2.88 (d, *J* = 11.7 Hz, 1H), 2.35–2.33 (m, 1H), 2.02 (s, 3H), 1.89–1.85 (m, 4H); *m*/*z* 425.19 [M + H]^+^. HRMS (ES^+^): *m*/*z* [M + H]^+^ calcd for C_22_H_26_FN_6_O_2_, 425.2101; found, 425.2088.

### *N*-(4-Fluoro-3-(3-(piperidin-1-yl)imidazo[1,2-*a*]pyrimidin-7-yl)phenyl)pyrrolidine-1-carboxamide (**34**)

**34** was synthesized by an analogous method to **1**, from **87** (0.2 g, 0.66 mmol), **96b** (0.567 g, 1.32 mmol), and zinc bromide (0.148 g, 0.66 mmol) with purification by prep. HPLC to yield **34** (0.021 g, 0.061 mmol, 9%). ^1^H NMR (DMSO-*d*_6_): δ 8.64 (d, *J* = 7.2 Hz, 1H), 8.43 (s, 1H), 8.23–8.20 (m, 1H), 7.80–7.76 (m, 1H), 7.45 (s, 1H), 7.41 (d, *J* = 6.9 Hz, 1H), 7.26 (dd, *J* = 9.5 and 10.7 Hz, 1H), 3.41–3.37 (m, 4H), 3.01–2.98 (m, 4H), 1.88–1.84 (m, 4H), 1.76–1.71 (m, 4H), 1.62–1.58 (m, 2H); ^13^C NMR (DMSO-*d*_6_): δ 156.7, 154.8, 154.4, 151.1 (d, *J*_CF_ = 2.7 Hz), 144.3, 138.0, 135.0, 131.7, 125.2 (d, *J*_CF_ = 11.6 Hz), 123.11, 121.1, 116.7 (d, *J*_CF_ = 23.8 Hz), 108.8 (d, *J*_CF_ = 11.4 Hz), 52.6, 46.2, 25.9, 25.5, 24.1; HRMS (ES^+^): *m*/*z* [M + H]^+^ calcd for C_22_H_26_FN_6_O, 409.2147; found, 409.2154.

### *N*-(4-Fluoro-3-(3-(piperazin-1-yl)imidazo[1,2-*a*]pyrimidin-7-yl)phenyl)pyrrolidine-1-carboxamide (**35**)

**35** was synthesized by an analogous method to **1**, from **87** (0.3 g, 1 mmol), **96c** (0.693 g, 1.1 mmol), and zinc bromide (0.045 g, 0.19 mmol) to give crude **Boc**-**35**. This was treated with TFA (3 mL)/DCM (6 mL), the solvent was evaporated, and crude material was purified by prep. HPLC to give **35** as the TFA salt (0.092 g, 0.23 mmol, 23% over two steps). ^1^H NMR (DMSO-*d*_6_): δ 9.06–8.98 (m, 3H), 8.45 (s, 1H), 8.34–8.31 (m, 1H), 7.91 (s, 1H), 7.78–7.74 (m, 1H), 7.73–7.70 (m, 1H), 7.35–7.30 (m, 1H), 3.42–3.34 (m, 8H), 3.30–3.27 (m, 4H), 1.89–1.85 (m, 4H); ^13^C NMR (DMSO-*d*_6_): δ 158.7, 158.4, 156.9, 154.9, 154.3, 138.2 (d, *J*_CF_ = 2.3 Hz), 133.6, 133.2, 124.3, 121.2, 117.0 (d, *J*_CF_ = 23.8 Hz), 48.4, 46.2, 43.4, 25.5; *m*/*z* 409.9 [M + H]^+^. *m*/*z* [M + H]^+^ calcd for C_21_H_25_N_7_OF, 410.2105; found, 410.2119.

### *N*-(3-(3-(4-Acetylpiperazin-1-yl)imidazo[1,2-*a*]pyrimidin-7-yl)-4-fluorophenyl)pyrrolidine-1-carboxamide (**36**)

To a mixture of *N*-(3-(2-aminopyrimidin-4-yl)-4-fluorophenyl)pyrrolidine-1-carboxamide (**87**, 0.5 g, 1.66 mmol) in 1,2-DCE (11 mL) were added **96d** (1.29 g, 2.49 mmol) and zinc bromide (0.187 g, 0.83 mmol) and stirred at 80 °C overnight. The solvent was evaporated and the residue was purified by column chromatography (0–10% MeOH/DCM), followed by prep. HPLC. The resulting solid was triturated with MeOH (5 mL) and dried to yield **36** (0.039 g, 0.08 mmol, 5%). ^1^H NMR (DMSO-*d*_6_): δ 8.78 (d, *J* = 7.2 Hz, 1H), 8.41 (s, 1H), 8.22 (dd, *J* = 2.8 and 7.1 Hz, 1H), 7.80–7.75 (m, 1H), 7.53 (s, 1H), 7.47–7.43 (m, 1H), 7.29–7.23 (m, 1H), 3.70–3.65 (m, 4H), 3.41–3.36 (m, 4H), 3.08–2.97 (m, 4H), 2.09 (s, 3H), 1.89–1.85 (m, 4H); ^13^C NMR (DMSO-*d*_6_): δ 168.9, 157.0, 154.6, 154.4, 151.7, 144.5, 138.1, 113.7, 131.9, 125.2 (d, *J*_CF_ = 11.8 Hz), 123.8, 123.2 (d, *J*_CF_ = 8.3 Hz), 121.2, 116.7 (d, *J*_CF_ = 23.8 Hz), 109.0 (d, *J*_CF_ = 11.1 Hz), 51.7, 51.4, 46.2, 25.5, 21.7; *m*/*z* 452.1 [M + H]^+^. *m*/*z* [M + H]^+^ calcd for C_21_H_23_N_5_O_3_F, 452.2210; found, 452.2229.

### *N*-(4-Fluoro-3-(3-(3-oxopiperazin-1-yl)imidazo[1,2-*a*]pyrimidin-7-yl)phenyl)pyrrolidine-1-carboxamide (**37**)

**37** was synthesized by an analogous method to **1**, from **87** (0.5 g, 1.66 mmol), **96e** (1.15 g, 2.49 mmol), and zinc bromide (0.187 g, 0.83 mmol) with purification by flash chromatography (0–10% MeOH/DCM), followed by trituration with MeOH to give **37** (0.06 g, 0.14 mmol, 8%). ^1^H NMR (DMSO-*d*_6_): δ 8.76 (d, *J* = Hz, 1H), 7.2 Hz, 8.41 (s, 1H), 8.21 (dd, *J* = 7.2 and 2.8 Hz, 1H), 8.03 (s, 1H), 7.81–7.76 (m, 1H), 7.55 (s, 1H), 7.42 (dd, *J* = 2.1 and 7.1 Hz, 1H), 7.25 (dd, *J* = 9.0 and 11.3 Hz, 1H), 3.69 (s, 2H), 3.42–3.36 (m, 6H), 3.24–3.20 (m, 2H), 1.89–1.84 (m, 4H); ^13^C NMR (DMSO-*d*_6_): δ 167.3, 156.8, 154.8, 154.4, 151.7, 144.7, 138.1 (d, *J*_CF_ = 2.1 Hz), 132.3, 132.1, 125.1 (d, *J*_CF_ = 11.6 Hz), 124.4, 123.3 (d, *J*_CF_ = 8.1 Hz), 121.2, 116.6 (d, *J*_CF_ = 23.9 Hz), 109.0 (d, *J*_CF_ = 11.1 Hz), 54.7, 48.2, 46.2, 40.8, 25.5; *m*/*z* 424.0 [M + H]^+^. *m*/*z* [M + H]^+^ calcd for C_21_H_23_N_7_O_2_F, 424.1897; found, 424.1902.

### *N*-(4-Fluoro-3-(3-(2-methylmorpholino)imidazo[1,2-*a*]pyrimidin-7-yl)phenyl)pyrrolidine-1-carboxamide (**38**)

A mixture of **87** (0.15 g, 0.5 mmol), **96f** (0.345 g, 0.75 mmol), and zinc bromide (0.056 g, 0.25 mmol) 1,2-DCE (5 mL) was heated to reflux for 6 h. The reaction was cooled to RT, solvent was evaporated, and the residue was purified by prep. HPLC to give **38** (0.036 g, 0.13 mmol, 17%). ^1^H NMR (DMSO-*d*_6_): δ 8.76 (d, *J* = 7.2 Hz, 1H), 8.43 (s, 1H), 8.23–8.19 (m, 1H), 7.80–7.75 (m, 1H), 7.50 (s, 1H), 7.42 (d, *J* = 6.8 Hz, 1H), 7.28 (dd, *J* = 9.5 and 10.7 Hz, 1H), 3.93–3.89 (m, 1H), 3.83–3.76 (m, 2H), 3.41–3.37 (m, 4H), 3.20 (d, *J* = 11.3 Hz, 1H), 3.13 (d, *J* = 11.4 Hz, 1H), 2.91–2.84 (m, 1H), 2.59 (dd, *J* = 10.5 and 10.9 Hz, 1H), 1.88–1.85 (m, 4H), 1.14 (d, *J* = 6.2 Hz, 3H); ^13^C NMR (DMSO-*d*_6_): δ 156.7, 154.7, 154.4, 151.5, 144.5, 138.0, 133.8, 131.9, 125.2 (d, *J*_CF_ = 11.6 Hz), 123.4, 121.2, 116.7 (d, *J*_CF_ = 24.0 Hz), 108.9 (d, *J*_CF_ = 11.5 Hz), 71.7, 66.3, 57.5, 51.0, 46.2, 25.5, 19.1; HRMS (ES^+^): *m*/*z* [M + H]^+^ calcd for C_22_H_26_FN_6_O_2_, 425.2096; found, 425.2100.

### (*R*)-*N*-(4-Fluoro-3-(3-(3-methylmorpholino)imidazo[1,2-*a*]pyrimidin-7-yl)phenyl)pyrrolidine-1-carboxamide (**39**)

A mixture of **100a** (0.343 g, 0.743 mmol) and 10% activated palladium on carbon (0.040 g, 0.037 mmol) was cooled to −78 °C and MeOH (7.43 mL) was added. The flask was purged with N_2_ and H_2_, and the resulting mixture stirred at RT under a H_2_ atmosphere overnight. The RM was filtered through a celite pad, washed with DCM (5 × 20 mL), and the eluent was concentrated to dryness and dried under high vacuum to afford (*R*)-4-fluoro-3-(3-(3-methylmorpholino)imidazo[1,2-*a*]pyrimidin-7-yl)aniline which was used without purification. The crude material was taken up in DCM (10 mL) to which CDI (0.213 g, 1.314 mmol) and DIPEA (0.229 mL, 1.314 mmol) were added and stirred overnight. Pyrrolidine (0.110 mL, 1.314 mmol) was added dropwise and the RM was stirred at RT for 4.5 h. Further pyrrolidine (0.02 mL) was added and the reaction was stirred at RT for a further 30 min, then poured into 2 M NaOH (50 mL), and extracted with DCM (5 × 20 mL). The organic layers were combined, dried over Na_2_SO_4_, filtered, concentrated, and the crude material was purified by flash chromatography (0–50% EtOAc/EtOH (3:1)/cyclohexane). Fractions containing the product were further purified by flash chromatography (0–50% EtOAc/EtOH (3:1)/cyclohexane) to give **39** (0.127 g, 0.34 mmol, 46%). ^1^H NMR (DMSO-*d*_6_): δ 8.79 (d, *J* = 7.2 Hz, 1H), 8.41 (s, 1H), 8.21 (dd, *J* = 7.1, 2.8 Hz, 1H), 7.77 (ddd, *J* = 9.0, 4.4, 2.8 Hz, 1H), 7.68 (s, 1H), 7.44 (dd, *J* = 7.2, 2.1 Hz, 1H), 7.25 (dd, *J* = 11.4, 9.0 Hz, 1H), 3.91–3.80 (m, 2H), 3.72 (ddd, *J* = 11.3, 9.5, 2.9 Hz, 1H), 3.45–3.33 (m, 5H), 3.21 (dqd, *J* = 9.1, 6.2, 2.8 Hz, 1H), 3.04 (dddd, *J* = 21.5, 12.0, 9.2, 3.0 Hz, 2H), 1.92–1.80 (m, 4H), 0.78 (d, *J* = 6.3 Hz, 3H); ^13^C NMR (DMSO-*d*_6_): δ 156.7, 154.8, 154.4, 152.0, 144.7, 138.0, 131.9 (d, *J*_CF_ 9.1 Hz), 127.5, 125.2 (d, *J*_CF_ 11.8 Hz), 123.2 (d, *J*_CF_ 8.1 Hz), 121.2, 116.7 (d, *J*_CF_ 23.9 Hz), 109.1 (d, *J*_CF_ 11.1 Hz), 72.6, 67.3, 55.5, 52.4, 46.2, 25.5, 14.6; HRMS (ES^+^): *m*/*z* [M + H]^+^ calcd for C_22_H_26_FN_6_O_2_, 425.2096; found, 425.2093.

### (*S*)-*N*-(4-Fluoro-3-(3-(3-methylmorpholino)imidazo[1,2-*a*]pyrimidin-7-yl)phenyl)pyrrolidine-1-carboxamide (**40**)

**40** was prepared by an analogous method to **39**, from **100b** (0.37 g, 0.802 mmol) using 10% activated palladium on carbon (0.0427 g, 0.04 mmol), then CDI (0.155 g, 0.953 mmol), DIPEA (0.166 mL, 0.953 mmol), and pyrrolidine (0.080 mL, 0.953 mmol), purifying by prep. HPLC to yield **42** (0.12 g, 0.28 mmol, 35% over two steps). ^1^H NMR (DMSO-*d*_6_): δ 8.80 (d, *J* = 7.2 Hz, 1H), 8.43 (s, 1H), 8.24–8.20 (m, 1H), 7.80–7.76 (m, 1H), 7.69 (s, 1H), 7.44 (d, *J* = 6.7 Hz, 1H), 7.26 (dd, *J* = 9.5 and 10.8 Hz, 1H), 3.90–3.83 (m, 2H), 3.75–3.69 (m, 1H), 3.42–3.35 (m, 5H), 3.24–3.19 (m, 1H), 3.11–2.98 (m, 2H), 1.90–1.83 (m, 4H), 0.79 (d, *J* = 6.2 Hz, 3H); ^13^C NMR (DMSO-*d*_6_): δ 156.7, 154.8, 154.4, 152.0, 144.7, 138.0, 131.9 (d, *J*_CF_ 9.5 Hz), 127.5, 125.2 (d, *J*_CF_ 11.9 Hz), 123.3 (d, *J*_CF_ 8.0 Hz), 121.2, 116.7 (d, *J*_CF_ 23.9 Hz), 109.1 (d, *J*_CF_ 11.2 Hz), 72.6, 67.2, 55.5, 52.4, 46.2, 25.5, 14.5; HRMS (ES^+^): *m*/*z* [M + H]^+^ calcd for C_22_H_26_FN_6_O_2_, 425.2101; found, 425.2113.

### *N*-(3-(3-((2*S*,6*R*)-2,6-Dimethylmorpholino)imidazo[1,2-*a*]pyrimidin-7-yl)-4-fluorophenyl)pyrrolidine-1-carboxamide (**41**)

To a mixture of **87** (3.5 g, 11.62 mmol) in 1,2-DCE (77 mL), **96g** (8.55 g, 17.42 mmol) and zinc bromide (1.308 g, 5.81 mmol) were added and then stirred at 80 °C under a N_2_ atmosphere for 2 h, cooled to RT, and stirred overnight. The solvent was evaporated, and the residue purified by flash chromatography (0–10% MeOH/DCM). The resulting solid was triturated with MeOH (20 mL), and the solids were collected by filtration to give **41** (1.0 g, 2.28 mmol, 20%). ^1^H NMR (DMSO-*d*_6_): δ 9.16 (d, *J* = 7.2 Hz, 1H), 8.49 (s, 1H), 8.39 (dd, *J* = 7.1, 2.8 Hz, 1H), 8.01 (s, 1H), 7.87 (dd, *J* = 7.3, 1.7 Hz, 1H), 7.74 (ddd, *J* = 9.0, 4.4, 2.8 Hz, 1H), 7.35 (dd, *J* = 11.3, 9.0 Hz, 1H), 3.93–3.80 (m, 2H), 3.43–3.32 (m, 4H), 3.22 (d, *J* = 2.1 Hz, 2H), 2.55 (dd, *J* = 11.8, 10.2 Hz, 2H), 1.91–1.80 (m, 4H), 1.14 (d, *J* = 6.3 Hz, 6H); ^13^C NMR (DMSO-*d*_6_): δ 157.0, 155.1, 154.3, 141.3, 138.4, 134.9, 134.4, 125.2, 123.5 (d, *J*_CF_ = 11.1 Hz), 121.2, 117.2 (d, *J*_CF_ = 24.0 Hz), 113.2, 71.3, 56.5, 46.2, 25.5, 19.1; HRMS (ES^+^): *m*/*z* [M + H]^+^ calcd for C_23_H_28_FN_6_O_2_, 429.2252; found, 439.2227.

### *N*-(3-(3-(2,2-Dimethylmorpholino)imidazo[1,2-*a*]pyrimidin-7-yl)-4-fluorophenyl)pyrrolidine-1-carboxamide (**42**)

A mixture of **87** (0.2 g, 0.66 mmol), **96h** (0.486 g, 0.99 mmol), and zinc bromide (0.223 g, 0.99 mmol) in MeCN (4 mL) was stirred at 40 °C for 18 h. The solvent was evaporated, and the crude product purified by prep. HPLC to give **42** (0.134 g, 46% yield). ^1^H NMR (DMSO-*d*_6_): δ 8.98 (d, *J* = 7.0 Hz, 1H), 8.48 (s, 1H), 8.38–8.35 (m, 1H), 7.90 (s, 1H), 7.80 (d, *J* = 7.8 Hz, 1H), 7.77–7.73 (m, 1H), 7.35 (dd, *J* = 7.4 and 10.6 Hz, 1H), 3.89–3.85 (m, 4H), 3.42–3.37 (m, 4H), 3.00–2.97 (m, 2H), 2.91–2.88 (m, 2H), 1.89–1.85 (m, 4H), 1.33 (s, 6H); ^13^C NMR (DMSO-*d*_6_): 157.3, 154.9, 154.3, 141.6, 138.4, 134.8, 134.4, 125.1 (d, *J*_CF_ = 8.4 Hz), 123.5 (d, *J*_CF_ = 11.1 Hz), 121.2, 117.1 (d, *J*_CF_ = 23.7 Hz), 114.0, 113.1 (d, *J*_CF_ = 11.6 Hz), 71.2, 60.5, 60.4, 51.7, 46.2, 25.5, 24.9; HRMS (ES^+^): *m*/*z* [M + H]^+^ calcd for C_23_H_28_FN_6_O_2_, 439.2258; found, 439.2252.

### *N*-(3-(3-(3,3-Dimethylmorpholino)imidazo[1,2-*a*]pyrimidin-7-yl)-4-fluorophenyl)pyrrolidine-1-carboxamide (**43**)

A mixture of **87** (0.676 g, 2.243 mmol), **96i** (1.642 g, 3.347 mmol), and zinc bromide (0.777 g, 3.45 mmol) in MeCN (12 mL) was stirred at 60 °C for 4 h and evaporated to dryness. The crude material was purified by flash chromatography (2% MeOH/DCM) and then by prep. HPLC to obtain **43** (0.11 g, 0.25 mmol, 12%). ^1^H NMR (CD_3_OD): δ 9.26 (d, *J* = 7.2 Hz, 1H), 8.46 (dd, *J* = 6.8 and 2.8 Hz, 1H), 8.15 (s, 1H), 8.10 (d, *J* = 6.8 Hz, 1H), 7.63 (ddd, *J* = 8.8, 7.2 and 2.8 Hz, 1H), 7.29 (dd, *J* = 11.2 and 9.2 Hz, 1H), 4.01–3.78 (m, 2H), 3.65 (s, 2H), 3.49 (t, *J* = 6.8 Hz, 4H), 3.06–2.74 (m, 2H), 2.00 (t, *J* = 6.4 Hz, 4H), 1.54–0.80 (m, 6H); ^13^C NMR (DMSO-*d*_6_): δ 158.7, 158.4, 157.0, 155.1, 154.3, 141.9, 138.4, 135.0, 130.8, 125.1 (d, *J*_CF_ = 8.6 Hz), 123.6 (d, *J*_CF_ = 11.2 Hz), 121.2, 117.2 d, (*J*_CF_ = 23.3 Hz), 76.9, 67.7, 55.8, 48.3, 46.2, 25.5, 17.4; HRMS (ES^+^): *m*/*z* [M + H]^+^ calcd for C_23_H_28_FN_6_O_2_, 439.2258; found, 439.2265.

### *N*-(3-(3-(2-Oxa-7-azaspiro[4.4]nonan-7-yl)imidazo[1,2-*a*]pyrimidin-7-yl)-4-fluorophenyl)pyrrolidine-1-carboxamide (**44**)

**44** was prepared by an analogous method to **39**, from **100c** (0.283 g, 0.580 mmol) using 10% activated palladium on carbon (0.0309 g, 0.029 mmol), then CDI (0.125 g, 0.770 mmol), DIPEA (0.134 mL, 0.770 mmol), and pyrrolidine (0.064 mL, 0.770 mmol), purifying by flash chromatography (0–4% EtOAc/EtOH (3:1)/cyclohexanes) to yield **44** (0.07 g, 0.155 mmol, 27% over two steps). ^1^H NMR (DMSO-*d*_6_): δ 8.73 (d, *J* = 7.6 Hz, 1H), 8.40 (s, 1H), 8.20 (dd, *J* = 7.2 and 2.8 Hz, 1H), 7.76 (ddd, *J* = 8.8, 7.2 and 2.8 Hz, 1H), 7.40 (s, 1H), 7.36 (dd, *J* = 7.6 and 2.4 Hz, 1H), 7.24 (dd, *J* = 11.2 and 8.8 Hz, 1H), 3.84–3.77 (m, 2H), 3.71 (t, *J* = 8 Hz, 1H), 3.61 (t, *J* = 8.4 Hz, 1H), 3.42–3.34 (m, 6H), 3.26 (d, *J* = 9.2 Hz, 6H), 3.22 (d, *J* = 9.2 Hz, 6H), 2.06–1.95 (m, 4H), 1.86 (t, *J* = 6.8 Hz, 4H); ^13^C NMR (DMSO-*d*_6_): δ 156.7, 154.8, 154.4, 150.4, 144.0, 138.0, 133.5, 132.1, 125.3 (d, *J*_CF_ = 11.8 Hz), 123.0 (d, *J*_CF_ = 7.9 Hz), 121.1 (d, *J*_CF_ = 15.3 Hz), 116.8 (d, *J*_CF_ = 24.0 Hz), 108.7 (d, *J*_CF_ = 11.4 Hz), 77.1, 67.4, 60.8, 50.9, 49.7, 46.2, 37.7, 35.2, 25.5; HRMS (ES^+^): *m*/*z* [M + H]^+^ calcd for C_24_H_28_FN_6_O_2_, 451.2252; found, 451.2234.

### *N*-(4-Fluoro-3-(3-(isopropylamino)imidazo[1,2-*a*]pyrimidin-7-yl)phenyl)pyrrolidine-1-carboxamide (**45**)

**45** was synthesized by an analogous method to **39**, from **100d** (0.27 m, 0.64 mmol) and 10% activated palladium on carbon (0.034 g, 0.03 mmol), followed by DIPEA (233 μL, 1.33 mmol), CDI (0.216 g, 1.33 mmol), and pyrrolidine (111 μL, 1.33 mmol), purifying by prep. HPLC to give **45** (0.056 g, 0.15 mmol, 22%). ^1^H NMR (DMSO-*d*_6_): δ 8.61 (d, *J* = 7.3 Hz, 1H), 8.41 (s, 1H), 8.20–8.16 (m, 1H), 7.78–7.73 (m, 1H), 7.35 (d, *J* = 7.0 Hz, 1H), 7.23 (dd, *J* = 9.5 and 10.9 Hz, 1H), 7.16 (s, 1H), 5.37 (d, J = 6.3 Hz, 1H), 3.56–3.46 (m, 1H), 3.42–3.36 (m, 1H), 1.91–1.84 (m, 4H), 1.22 (d, *J* = 6.3 Hz, 6H); ^13^C NMR (DMSO-*d*_6_): δ 154.7, 154.4, 148.8, 142.9, 138.0, 131.3, 130.6, 125.5 (d, *J*_CF_ = 12.1 Hz), 122.7 (d, *J*_CF_ = 8.1 Hz), 121.1, 118.8, 116.5 (d, *J*_CF_ = 23.9 Hz), 108.0 (d, *J*_CF_ = 11.3 Hz), 46.6, 46.2, 25.5, 23.2; HRMS (ES^+^): *m*/*z* [M + H]^+^ calcd for C_20_H_24_FN_6_O, 383.1996; found, 383.1999.

### *N*-(4-Fluoro-3-(3-((4-methoxybutan-2-yl)amino)imidazo[1,2-*a*]pyrimidin-7-yl)phenyl)pyrrolidine-1-carboxamide (**46**)

**46** was prepared by an analogous method to **39** from **100e** (0.24 g, 0.53 mmol) and 10% activated palladium on carbon (0.028 g, 0.03 mmol), followed by DIPEA (0.188 mL, 1.08 mmol), CDI (0.175 g, 1.08 mmol), and pyrrolidine (0.090 mL, 1.08 mmol), purifying by prep. HPLC to yield **46** (0.098 g, 0.24 mmol, 44%). ^1^H NMR (DMSO-*d*_6_): δ 8.64 (d, *J* = 7.2 Hz, 1H), 8.41 (s, 1H), 8.20–8.17 (m, 1H), 7.79–7.74 (m, 1H), 7.38–7.35 (m, 1H), 7.27–7.20 (m, 2H), 5.39 (d, *J* = 6.5 Hz, 1H), 3.57–3.50 (m, 1H), 3.44–3.32 (m, 5H, under solvent signal), 3.31–3.28 (m, 4H), 1.90–1.83 (m, 4H), 1.19 (d, *J* = 6.4 Hz, 3H); ^13^C NMR (DMSO-*d*_6_): δ 156.7, 154.7, 154.4, 149.1, 143.0, 137.9, 130.9 (d, *J*_CF_ = 11.1 Hz), 125.5 (d, *J*_CF_ = 11.8 Hz), 122.8 (d, *J*_CF_ = 7.4 Hz), 121.1, 119.3, 116.7 (d, *J*_CF_ = 23.8 Hz), 108.1 (d, *J*_CF_ = 11.3 Hz), 76.2, 58.8, 50.7, 46.2, 25.5, 18.4; HRMS (ES^+^): *m*/*z* [M + H]^+^ calcd for C_21_H_26_FN_6_O_2_, 413.2101; found, 413.2083.

## Supporting Information Available

Experimental information, synthesis of intermediates, methods for parasitology and modeling, molecular ESP maps and HPLC traces of key compounds, *in vitro* and *in vivo* assays, and *in vivo* pharmacokinetics (PDF)
Homology model of *L. donovani* protease subunits β4 and β5 (PDB)
Molecular formula strings for key compounds (CSV)

## Supplementary Material

jm1c00047_si_001.pdf

jm1c00047_si_002.pdb

## Notes

All regulated procedures on living animals in Dundee were carried out under the authority of a project license issued by the Home Office under the Animals (Scientific Procedures) Act 1986, as amended in 2012 (and in compliance with EU Directive EU/2010/63). License applications have been approved by the University’s Ethical Review Committee (ERC) before submission to the Home Office. The ERC has a general remit to develop and oversee policy on all aspects of the use of animals on University premises and is a subcommittee of the University Court, its highest governing body. All animal studies were reviewed by GlaxoSmithKline’s (GSK) internal ethical review committee and performed in accordance with Animals (Scientific Procedures) Act 1986 and the GSK Policy on the Care, Welfare, and Treatment of Laboratory Animals (UK 1986).

## Abbreviations

CLND: chemiluminescent nitrogen detection
ESP: electrostatic surface potential
FaSSIF: fasted state simulated intestinal fluid
FMO: Frontier molecular orbital method
INMAC: intracellular *L. donovani* assay
MoA: mechanism of Action
PFI: property forecast index
PIE, PIEDA: pair interaction energy decomposition analysis
VL: visceral leishmaniasis
