# Supplementary material for: Scaffold-Hopping Strategy on a Series of Proteasome Inhibitors Led to a Preclinical Candidate for the Treatment of Visceral Leishmaniasis
Source: J Med Chem. 2021 Apr 27;64(9):5905–30. doi: 10.1021/acs.jmedchem.1c00047 (PMC8154566; doi:10.1021/acs.jmedchem.1c00047)
Supplement: Supplementary file 1 — jm1c00047_si_001.pdf [file jm1c00047_si_001.pdf]

Supporting Information

A scaffold-hopping strategy on a series of proteasome inhibitors which led to a preclinical candidate for the treatment of visceral leishmaniasis

Michael Thomas,[a,§] Stephen Brand,[a,§] Manu De Rycker,[a] Fabio Zuccotto,[a] Iva Lukac,[a] Peter G. Dodd,[a] Eun-Jung Ko,[a] Sujatha Manthri,[a] Kate McGonagle,[a] Maria Osuna-Cabello,[a] Jennifer Riley,[a] Caterina Pont,[a] Frederick Simeons,[a] Laste Stojanovski,[a] John Thomas,[a] Stephen Thompson,[a] Elisabet Viayna,[a] Jose M. Fiandor,[b] Julio Martin,[b] Paul G. Wyatt,[a] Timothy J. Miles,[b] Kevin D. Read,[a] Maria Marco[b,*], Ian H. Gilbert[a,*]

a. Drug Discovery Unit, Wellcome Centre for Anti-Infectives Research, Division of Biological Chemistry, University of Dundee, Dundee, DD1 5EH, UK.

b. Global Health R&D, GlaxoSmithKline, Tres Cantos 28760, Spain

§ These authors contributed equally to the project

* Authors for correspondence: maria.m.marco@gsk.com; i.h.gilbert@dundee.ac.uk

Contents

General Experimental Information S-2

Synthesis of intermediates S-3

In vitro assays S-32

In vivo pharmacokinetics S-32

Molecular Modelling S-34

HPLC Traces of Key Compounds S-36

References S-58

General Experimental Information

Chemicals and solvents were purchased from the Aldrich Chemical Company, Fluka, ABCR, VWR, Acros, Fluorochem and Alfa Aesar and were used as received. Air- and moisture-sensitive reactions were carried out under an inert atmosphere of argon in oven-dried glassware. Analytical thin-layer chromatography (TLC) was performed on pre-coated TLC plates (layer 0.20 mm silica gel 60 with fluorescent indicator UV254, from Merck). Developed plates were air-dried and analyzed under a UV lamp (UV254/365 nm). Flash column chromatography was performed using pre-packed silica gel cartridges (230-400 mesh, 40–63 μm, from SiliCycle) using a Teledyne ISCO Combiflash Companion, or Combiflash Retrieve. ^{1}H NMR and ^{13}C NMR spectra were recorded on a Bruker Avance DPX 500 spectrometer (^{1}H at 500.1 MHz, ^{13}C at 125.8 MHz). Chemical shifts (δ) are expressed in ppm recorded using the residual solvent as the internal reference in all cases. Signal splitting patterns are described as singlet (s), doublet (d), triplet (t), quartet (q), multiplet (m), broad (b), or a combination thereof. Coupling constants (*J*) are quoted to the nearest 0.1 Hz. LC-MS analyses were performed with either an Agilent HPLC 1100 series connected to a Bruker Daltonics MicrOTOF, or an Agilent Technologies 1200 series HPLC connected to an Agilent Technologies 6130 quadrupole LC/MS, where both instruments were connected to an Agilent diode array detector. Mobile phase was water/acetonitrile + 0.1% HCOOH, or water/acetonitrile + 0.1% NH3; linear gradient 80:20 to 5:95 over 3.5 min, and then held for 1.5 min; flow rate 0.5 mL min^{-1}. All intermediates had a measured purity ≥ 90% and all assay compounds had a measured purity of ≥ 95% as determined using this analytical LC-MS system (TIC and UV). High resolution electrospray measurements were performed on a Bruker Daltonics MicrOTOF mass spectrometer. Microwave-assisted chemistry was performed using a Biotage Initiator Microwave Synthesizer.

Synthesis of intermediates

[12]**2-Bromo-1-(4-fluoro-3-nitrophenyl)ethan-1-one (47a)**

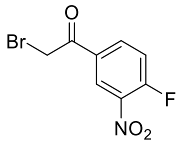

To 1-(2-fluoro-5-nitro-phenyl)ethanone (5.52 g, 30.1 mmol) in THF (100 mL) was added trimethyl(phenyl)ammonium bromide (10.3 g, 28 mmol) in THF (50 mL) drop-wise and the mixture stirred for 18 h. The resulting mixture was filtered to remove solid, concentrated and purified by flash chromatography (0-60% EtOAc/heptane) to give crude **47a** (6.5 g, 22.3 mmol, 81% crude yield), which was used in subsequent steps without further purification.

4-(2-(4-Fluoro-3-nitrophenyl)imidazo[1,2-*a*]pyrimidin-6-yl)morpholine (49a)

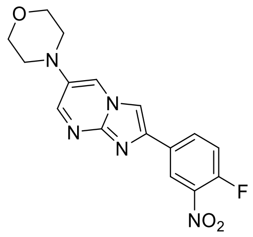

A mixture of crude **47a** (0.475 g, 1.813 mmol) and 5-morpholinopyrimidin-2-amine (**48**, 0.315 g, 1.748 mmol) in MeCN (12 mL) was heated at 60 ºC for 4 days. The reaction was partitioned between 10% MeOH/DCM (50 mL) and aqueous NaOH (2M, 100 mL). The phases were separated, and the aqueous layer extracted with DCM (50 mL). The combined organics were dried over Na_2SO_4, filtered and concentrated and the resulting solid triturated with MeCN to give **49a** as a brown pale solid (0.33 g, 0.96 mmol, 53%). ^{1}H NMR (DMSO-d_6): δ 8.78 – 8.61 (m, 2H), 8.44 – 8.31 (m, 3H), 7.69 (dd, J = 11.2, 8.7 Hz, 1H), 3.87 – 3.74 (m, 4H), 3.20 – 3.06 (m, 4H). m/z 344.2 [M + H]$^+$.

2-Fluoro-5-(6-morpholinoimidazo[1,2-*a*]pyrimidin-2-yl)aniline (50a)

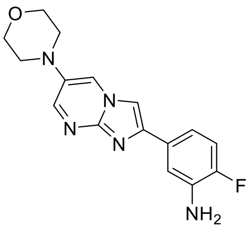

To a suspension of **49b** (0.35 g, 0.961 mmol) in THF/ EtOH (96 mL) was added Nickel Raney (0.13 g, 2.214 mmol) and the resulting suspension stirred at RT under H_2 overnight. More Nickel Raney (0.13 g, 2.214 mmol) was added and the mixture was stirred again at RT overnight under H_2. A third addition of Nickel Raney (0.13 g, 2.214 mmol) and further stirring at RT under H_2 for two days drove the reaction to completion. The reaction was filtered through celite, concentrated and the residue purified by flash chromatography (5% MeOH/DCM) to yield **50b** (0.32 g, quant. yield). ^{1}H NMR (DMSO-d_6): δ 8.67 (d, J = 2.8 Hz, 1H), 8.46 (d, J = 2.8 Hz, 1H), 8.10 (s, 1H), 7.48 (dd, J = 9.1, 2.0 Hz, 1H), 6.97 – 7.19 (m, 2H), 5.30 (br s, 2H), 3.65 – 3.89 (m, 4H), 3.11 – 3.21 (m, 4H). *m*/*z* 313.0 $[M + H]^+$.

N-(3-Acetylphenyl)pyrrolidine-1-carboxamide (51b)

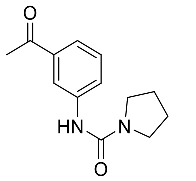

To 1-(3-aminophenyl)ethanone (1 g, 7.4 mmol) in 1,4-dioxane (10 mL) was added DMAP (0.09 g, 0.74 mmol) and pyrrolidine-1-carbonyl chloride (0.99 g, 7.40 mmol) and stirred at 90 ºC overnight. After cooling, the RM was diluted with aq. NaOH (20 mL, 2M) and extracted with EtOAc (2 x 30 mL). The organics were combined, dried over $MgSO_4$, filtered and concentrated. The residue was purified by flash chromatography (0 – 100% EtOAc / heptane) to give **51b** (0.89 g, 3.79 mmol, 51%).

^{1}H NMR (DMSO-d_6): δ 8.34 (s, 1H), 8.11 – 8.09 (m, 1H), 7.86 – 7.83 (m, 1H), 7.55 – 7.52 (m, 1H), 7.40 – 7.26 (m, 1H), 3.41 – 3.37 (m, 4H), 2.55 (s, 3H), 1.89 – 1.84 (m, 4H); *m/z* 233.2 [M + H]$^+$.

N-(3-(2-Bromoacetyl)phenyl)pyrrolidine-1-carboxamide (52b)

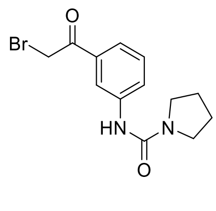

To a solution of **51b** (0.51 g, 2.2 mmol) in THF (5 mL) was added phenyltrimethylammonium tribromide (0.826 g, 2.20 mmol) and stirred at RT overnight. The RM was filtered and the filtrate concentrated and purified by flash chromatography (0 – 100% EtOAc / hexane) to give **52b** as a mixture with the dibrominated compound, which was used without further purification in the next step (0.53 g, 0.89 mmol, 40% crude yield). *m/z* 311.1, 313.1 [M + H]$^+$.

***N*-(3-(2-Bromoacetyl)-4-fluorophenyl)pyrrolidine-1-carboxamide (54)**

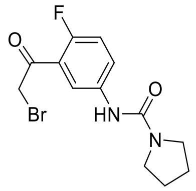

N-(3-acetyl-4-fluoro-phenyl)pyrrolidine-1-carboxamide (**53,** 11.7 g, 46.8 mmol) was dissolved in THF (400 mL), cooled to 0 °C and treated portion-wise with trimethyl(phenyl)ammonium tribromide (17.58 g, 46.8 mmol). The reaction was allowed to warm to RT and stirred overnight. Solvent was evaporated and the resulting solid dissolved in DCM (500 mL), washed with water (2 x 200 mL), brine (200 mL) and saturated aqueous $NaHCO_3$ solution (200 mL), then dried ($MgSO_4$), filtered and concentrated. The residual solid was triturated with EtOAc/Et_2O (1:1), then with EtOAc (200 mL),

collected by filtration and dried under vacuum to give **54** (12.47 g, 34.1 mmol, 73%). This contained approx. 5% **53** and 5% **55** and was used without further purification. ^{1}H NMR (DMSO-d_6): δ 8.40 (s, 1H), 8.02 (dd, *J* = 6.6 Hz, 2.9 Hz, 1H), 7.90 – 7.85 (m, 1H), 7.27 (dd, *J* = 10.9 Hz, 9.0 Hz, 1H), 4.78 (d, *J* = 2.4 Hz, 2H), 3.39 – 3.33 (m, 4H), 1.88 – 1.84 (m, 4H); m/z= 329.0 $[M+H]^+$

_N_-[3-(2,2-Dibromoacetyl)-4-fluoro-phenyl]pyrrolidine-1-carboxamide (55)

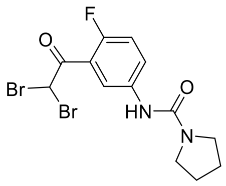

To a solution of *N*-(3-acetyl-4-fluoro-phenyl)pyrrolidine-1-carboxamide (**53**, 1.70 g, 6.45 mmol) in THF (50 mL) was added trimethylphenylammonium tribromide (9.70 g, 25.8 mmol) portion-wise, the reaction stirred at RT for 10 min then warmed to 60 °C and stirred for 12 h. The RM was filtered to remove solid, the solvent evaporated and the residue chromatographed (5 – 90% EtOAc/heptane) to give **55** (1.26 g, 3.1 mmol, 46%). ^{1}H NMR ($CDCl_3$): δ 8.07 – 8.03 (m, 1H), 7.71 – 7.67 (m, 1H), 7.15 (t, *J* = 10.0 Hz, 1H), 6.89 – 6.86 (m, 1H), 6.43 (s, 1H), 3.52 – 3.47 (m, 4H), 2.05 – 1.98 (s, 4H).

_N_-(3-(3-Amino-1,2,4-triazin-5-yl)-4-fluorophenyl)pyrrolidine-1-carboxamide (57) [3]

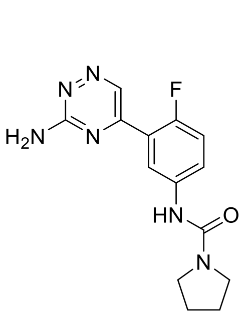

55 (1.47 g, 3.60 mmol) and morpholine (1.32 g, 15.13 mmol) in THF (10 mL) were stirred at 35 °C overnight, cooled, filtered to remove solid and solvent evaporated. Crude material was taken up in

MeOH (10 mL), aminoguanidine bicarbonate (0.487 g, 3.60 mmol) added, then acetic acid (0.044 g,0.73 mmol) added drop-wise over 5-10 min. RM was stirred at RT for 2 h, then temperature raised to 60 °C over 2 h and stirred overnight. After cooling to RT, volume was reduced by approx. half (no heating), and RM stirred at 0 °C for 1 h and the resulting solid collected, washed with cold 3:1 MeOH:water and dried under vacuum to yield **57** (0.295 g, 0.98 mmol, 26%) . ^{1}H NMR (DMSO-d_6): δ 8.93 (s, 1H), 8.40 (s, 1H), 8.18 (d, J = 6.3 Hz, 1H), 7.76 – 7.70 (m, 1H), 7.36 – 7.26 (m, 3H), 3.40 – 3.36 (m, 4H), 1.89 – 1.85 (m, 4H,); m/z 303.1 [M+H]$^+$

***N*-(3-(7-Bromoimidazo[1,2-*b*][1,2,4]triazin-3-yl)-4-fluorophenyl)pyrrolidine-1-carboxamide (58)**

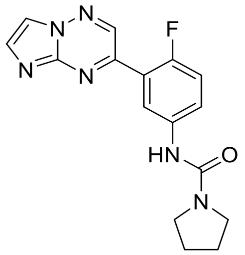

2-Bromo-1,1-diethoxy-ethane (0.7 g,3.56 mmol) in HBr (0.5 mL, 48%) / Water (0.5 mL) was stirred at 90 °C for 30 min, cooled and diluted with EtOH (2 mL). The solution was basified by addition of solid $NaHCO_3$, filtered to remove solid, and **57** (0.215 g, 0.71 mmol) added. RM was heated in a sealed tube at 100 °C overnight, solvent was evaporated, and crude material partitioned between EtOAc/water. The organics were washed with brine, dried over $MgSO_4$ and solvent evaporated. Et_2O was added and the resulting solid collected, washed with further ether and dried to yield **58** (0.09 g, 0.26 mmol, 36 %). ^{1}H NMR (DMSO-d_6): δ 9.05 (s, 1H), 8.47 – 8.43 (m, 2H), 8.30 – 8.26 (m, 1H), 8.08 (s, 1H), 7.88 – 7.83 (m, 1H), 7.33 (dd, J = 10.0, 10.2 Hz, 1H), 3.42 – 3.38 (m, 4H), 1.90 – 1.85 (m, 4H); m/z 327.1 [M+H]$^+$

N-(3-(7-bromoimidazo[1,2-*b*][1,2,4]triazin-3-yl)-4-fluorophenyl)pyrrolidine-1-carboxamide (59)

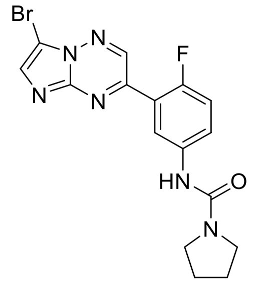

To **58** (0.085 g, 0.26 mmol) in acetic acid (1 mL) was added sodium acetate (0.032 g, 0.39 mmol) and bromine (0.046 g,0.29 mmol) and stirred at RT for 1 h. The RM was added drop-wise to sat. $NaHCO_3$/EtOAc and the organics separated. The aqueous layer was washed with further EtOAc and the combined organics washed with brine, dried over $MgSO_4$ and solvent evaporated. The residue was triturated with 3:1 ether/EtOAc and dried under vacuum to yield **59** (0.058 g, 0.13 mmol, 50 %). ^{1}H NMR (DMSO-d_6): δ 9.17 (s, 1H), 8.48 (s, 1H), 8.31 – 8.27 (m, 1H), 8.22 (s, 1H), 7.89 – 7.84 (m, 1H), 7.34 (d, J = 9.9 Hz, 1H), 3.43 – 3.37 (4H, s), 1.91 – 1.84 (4H, s); m/z = 405.0, 407.0 $[M+H]^+$

6-Bromo-2-(3-nitrophenyl)pyrazolo[1,5-*a*]pyrimidine (60)

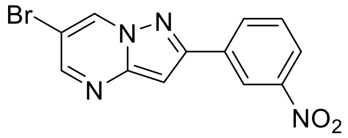

To a solution of 3-(3-nitrophenyl)-1*H*-pyrazol-5-amine (2.0 g, 9.8 mmol) in EtOH (150 mL) was added 2-bromopropanedial (1.63 g, 10.7 mmol) followed by acetic acid (0.5 mL) and stirred at 75 °C for 30 min. After cooling, the resulting solid was collected, washed with EtOAc and dried to give **60** (2.170 g, 6.46 mmol, 66%). ^{1}H NMR (DMSO-d_6): δ 967 (s, 1H), 8.81 (s, 1H), 8.66 (s, 1H), 8.48 (d, J = 7.5 Hz, 1H), 8.28 (d, J = 7.5 Hz, 1H), 7.84 – 7.79 (m, 1H), 7.55 (s, 1H) ; m/z = 318.0, 320.0 $[M+H]^+$

2-(3-Nitrophenyl)-6-phenylpyrazolo[1,5-*a*]pyrimidine (61)

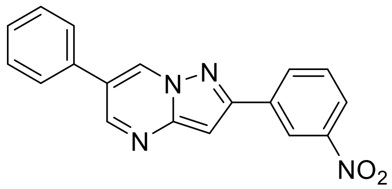

To a nitrogen purged solution of phenylboronic acid (0.382 g, 3.13 mmol), **60** (0.5 g, 1.57 mmol) and potassium acetate (0.307 g, 3.13 mmol) in dioxane (15 mL) was added Tetrakis(triphenylphosphine)palladium(0) (0.09 g, 0.078 mmol) and the mixture heated in a microwave (120 °C, 1 h). After cooling, the resulting solid was collected, washed with water and dried to give **61** (0.35 g, 0.99 mmol, 64 %) which was used in the next step without purification. m/z = 317.1 $[M+H]^+$

***N*-(3-Ethynylphenyl)pyrrolidine-1-carboxamide (62)**

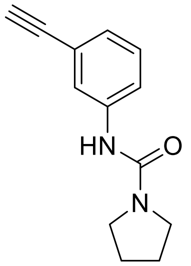

To a solution of 3-ethynylaniline (2.5 g, 21.3 mmol) and DMAP (0.26 g, 2.13 mmol) in DCM (30 mL) / Pyridine (10mL) was added pyrrolidine-1-carbonyl chloride (3.42 g, 25.6 mmol) and the solution stirred at 50 °C overnight. After cooling, the RM was washed with water (15 mL), 1M HCl (15 mL) and brine (15 mL), filtered through a phase separator and the solvent evaporated. Ether (10 mL) was added and the resulting solid collected, washed with further ether and dried to give **62** (3.78 g, 16.8 mmol, 79%). ^{1}H NMR (DMSO-d_6): δ 8.19 (s, 1H), 7.71 – 7.69 (m, 1H), 7.57 – 7.53 (m, 1H), 7.25 – 7.21 (m, 1H), 7.02 (d, J = 7.6 Hz, 1H), 4.11 (s, 1H), 3.38 – 3.34 (m, 4H), 1.88 – 1.82 (m, 4H); m/z = 215.1 $[M+H]^+$

N-(3-((2-Amino-5-phenylpyridin-3-yl)ethynyl)phenyl)pyrrolidine-1-carboxamide (63)

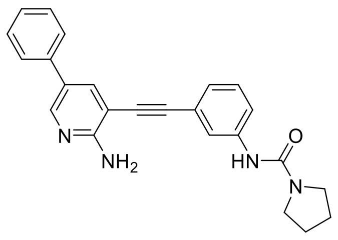

To a solution of **62** (0.086 g, 0.40 mmol) and 3-bromo-5-phenylpyridin-2-amine (0.1 g, 0.40 mmol) in DMF (3 mL) was added Tetrakis (0.014 g, 0.02 mmol), copper iodide (0.008 g, 0.04 mmol) and NEt_3 (0.243 g, 2.4 mmol) and stirred at 80 °C overnight. After cooling, the RM was partitioned between DCM (15 mL) and sat. NH_4Cl (15 mL). The organics were washed with brine (15 mL), dried over $MgSO_4$ and solvent evaporated. Crude material was chromatographed (0 – 100% EtOAc / heptane) to give **63** (0.075 g, 0.19 mmol, 46%. ^1H NMR (DMSO-d_6): δ 8.33 – 8.31 (m, 1H), 7.86 (d, J = 2.4 Hz, 1H), 7.56 – 7.53 (m, 2H), 7.48 – 7.44 (m, 2H), 7.42 – 7.40 (m, 1H), 7.38 – 7.34 (m, 1H), 7.32 – 7.29 (m, 1H), 7.22 – 7.20 (m, 1H), 6.23 (s, 1H), 5.12 (s, 2H), 3.54 – 3.48 (m, 4H), 2.04 – 2.00 (m, 4H); m/z = 383.2 $[M+H]^+$

N-(3-ethynyl-4-fluorophenyl)pyrrolidine-1-carboxamide (66)

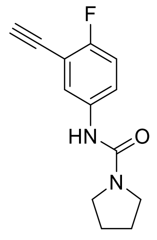

To a suspension of 2-ethynyl-1-fluoro-4-nitro-benzene (2 g, 12.1 mmol) in EtOH (30 mL) was added iron (5.4 g, 97 mmol) and a solution of ammonium chloride (2.59 g, 48.5 mmol) in water (1 mL)and stirred at 75 °C for 2 h. The RM was filtered through celite, washed through with DCM and water, the layers separated and the aqueous washed with further DCM. The combined organics were washed with brine, dried over $MgSO_4$ and concentrated to give crude material which was used without

purification. To this crude 3-ethynyl-4-fluoro-aniline (0.966 g, 7.15 mmol) and DMAP (0.088 g, 0.715 mmol) in DCM (40 mL) / pyridine (10 mL) was added pyrrolidine-1-carbonyl chloride (1.15 g, 8.6 mmol) and stirred at 50 °C for 18 h. The mixture was cooled, washed with water, 1M HCl then brine and the organics evaporated. Ether was added and resulting solid collected, washed with ether and dried under vacuum to give **66** (1.3 g, 5.60 mmol, 78 %). ^{1}H NMR (DMSO-d_6): δ 8.35 (s, 1H), 7.85 – 7.82 (m, 1H), 7.70 – 7.65 (m, 1H), 7.31 – 7.26 (m, 1H), 4.55 (s, 1H), 3.49 – 3.44 (m, 4H), 2.00 – 1.95 (m, 4H); *m/z* 233.1 [M + H]$^+$.

***N*-(3-((3-amino-6-bromopyrazin-2-yl)ethynyl)-4-fluorophenyl)pyrrolidine-1-carboxamide (67)**

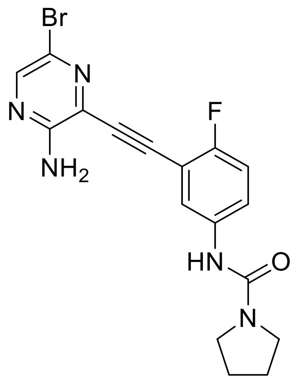

To 3,5-dibromopyrazin-2-amine (1.12 g, 4.43 mmol) and **66** (1.0286 g, 4.43 mmol) in DMF (18 mL) was added $PdCl_2(PPh_3)_2$ (0.1554 g, 0.22 mmol), copper iodide (0.0843 g, 0.44 mmol) then NEt_3 (0.4473 g, 4.43 mmol) and stirred for 30 minutes. Solvent was evaporated, EtOAc/water added, filtered to remove solid and the organics washed with brine, evaporated and chromatographed (20-100% EtOAc/heptane) to give **67** (0.425 g, 0.99 mmol, 23 %). ^{1}H NMR (DMSO-d_6): δ 8.29 (s, 1H), 8.16 (s, 1H), 7.93 (dd, *J* = 2.7, 6.4 Hz, 1H), 7.59 (ddd, *J* = 2.8, 4.7, 9.0 Hz, 1H), 7.25 (dd, *J* = 9.2, 9.2 Hz, 1H), 6.97 (s, 2H), 3.39 – 3.35 (m, 4H), 1.86 (dd, *J* = 6.7, 6.7 Hz, 4H). *m/z* 404.1/406.1 [M+H]$^+$.

N-(3-(2-Bromo-5H-pyrrolo[2,3-b]pyrazin-6-yl)-4-fluorophenyl)pyrrolidine-1-carboxamide (68)

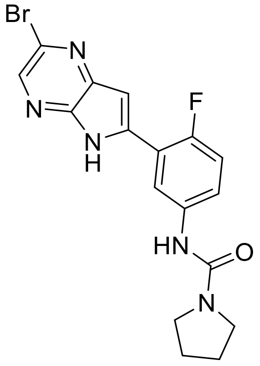

To **67** (0.388 g, 0.96 mmol) in NMP (6 mL) was added potassium *tert*-butoxide (0.3231 g, 2.88 mmol) and stirred at 75 °C for 2 h. After cooling, the RM was poured onto sat. aqueous NH_4Cl, and the resulting solid collected, washed with water and dried to give **68** (0.35 g, 0.82 mmol, 86 %). ^{1}H NMR (DMSO-d_6): δ 12.72 (s, 1H), 8.39 (s, 1H), 8.34 (s, 1H), 8.06 (dd, J = 2.6, 6.9 Hz, 1H), 7.61 – 7.57 (m, 1H), 7.33 – 7.28 (m, 1H), 6.93 – 6.91 (m, 1H), 3.42 – 3.37 (m, 4H), 1.88 (dd, J = 6.6, 6.6 Hz, 4H). m/z 404.1/406.1 $[M+H]^+$.

N-(3-(5-Bromofuro[2,3-b]pyridin-2-yl)-4-fluorophenyl)pyrrolidine-1-carboxamide (69)

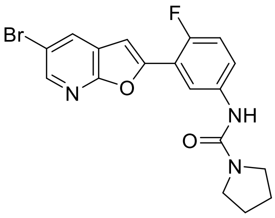

To a degassed solution of 5-bromo-3-iodopyridin-2-ol (0.8 g, 2.67 mmol), **66** (0.682 g, 2.93 mmol), copper iodide (0.0508 g, 0.27 mmol) and $PdCl_2(PPh_3)_4$ (0.187 g, 0.27 mmol) in DMF (27 mL), was added degassed NEt_3 (1.49 mL, 10.67 mmol) and stirred at 50 °C for 16 h. Solvent was evaporated and the mixture purified by flash chromatography (0 –30% EtOAc/EtOH (3:1) / cyclohexane) to give **69** (0.462 g, 1.14 mmol, 42 %). ^{1}H NMR ($CDCl_3$): δ 8.40 – 8.32 (m, 1H), 8.08 – 8.00 (m, 1H), 7.88

– 7.74 (m, 2H), 7.20 -7.10 (m, 2H), 6.35 – 6.22 (m, 1H), 3.58 – 3.40 (m, 4H), 2.06 – 1.97 (m, 4H); *m/z* 404.1, 406.1 [M + H]+.

N-(3-((6-Chloro-4-(4-methoxybenzyl)-3-oxo-3,4-dihydropyrazin-2-yl)ethynyl)-4-fluorophenyl)pyrrolidine-1-carboxamide (70)

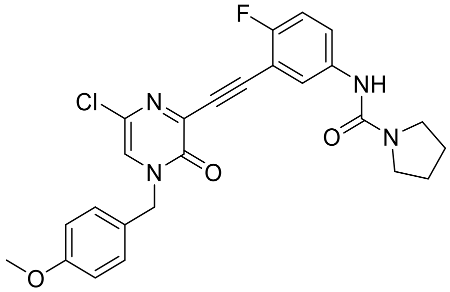

To **66** (2.57 g, 11.1 mmol), copper (I) iodide (0.042 g, 0.22 mmol), $Pd(PPh_3)_2Cl_2$ (0.052 g, 0.074 mmol) and 3,5-dichloro-1-[(4-methoxyphenyl)methyl]pyrazin-2-one (2.11 g, 7.4 mmol) was added DMF (30 mL) and NEt_3 (15.0 g, 148 mmol) and the mixture stirred at 80 °C for 2 h. The mixture was cooled, partitioned between EtOAc / sat. NH_4Cl, the aqueous layer extracted with EtOAc and the combined organics washed with brine, dried over $MgSO_4$ and solvent evaporated. The crude material was purified by flash chromatography (0 – 70% EtOAc / heptane) to give **70** (3.02 g, 6.0 mmol, 81 %). ^1H NMR ($CDCl_3$) δ 7.77 – 7.73 (m, 1H), 7.45 – 7.43 (m, 1H), 7.34 – 7.31 (m, 2H), 7.19 (s, 1H), 7.09 – 7.05 (m, 1H), 6.96 – 6.92 (m, 2H), 6.22 (bs, 1H), 5.06 (s, 2H), 3.84 (s, 3H), 3.50 – 3.45 (m, 4H), 2.03 – 1.98 (m, 4H); m/z [M+H]+ 481.3.

***N*-(3-(2-Chlorofuro[2,3-*b*]pyrazin-6-yl)-4-fluorophenyl)pyrrolidine-1-carboxamide (71)**

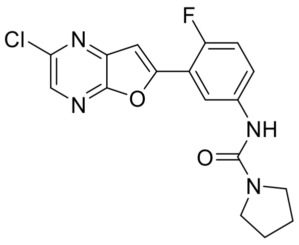

To a solution of **70** (1.5 g, 3.12 mmol) in DCM (25 mL) was added silver nitrate (0.028 g, 0.31 mmol) then trifluoroacetic acid (1.8 g, 15.6 mmol) and stirred for 0.5 h. Solvent was evaporated and the resulting solid triturated with ether and dried to give **71** (1.05 g, 2.77 mmol, 89 %). ^{1}H NMR ($CDCl_3$) δ 8.25 (s, 1H), 7.96 – 7.94 (m, 1H), 7.80 – 7.75 (m, 1H), 7.38 (d, *J* = 3.0 Hz, 1H), 7.18 (dd, *J* = 9.0, 10.6 Hz, 1H), 6.37 (s, 1H), 3.55 – 3.51 (m, 4H), 2.06 – 2.03 (m, 4H); m/z $[M+H]^+$ 361.1.

Ethyl 3-(2-fluoro-5-nitrophenyl)propiolate (72)

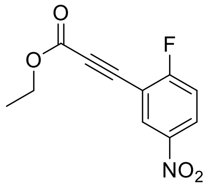

2-Ethynyl-1-fluoro-4-nitrobenzene (1.5 g, 9.1 mmol) was dissolved in THF (20 mL) under N_2 at -78 °C. Lithium diisopropylamide (13.6 mL, 13.6 mmol, 1.0 M in THF) was added, and the solution stirred at -78 °C for 1 h. After this time ethyl chloroformate (1.479 g, 13.63 mmol) was added drop-wise and the reaction stirred at -78 °C for 2 h. The RM was slowly added to sat. aq. $NaHCO_3$ (50 mL), extracted into EtOAc (3 x 75 mL), dried over $MgSO_4$ and solvent evaporated. Crude material was purified by flash chromatography (0 – 85% EtOAc / heptane) to give **72** (2.06 g, 8.25 mmol, 91 %). ^{1}H NMR ($CDCl_3$) δ 8.31 (dd, *J* = 2.8, 5.8 Hz, 1H), 8.17 (ddd, *J* = 2.8, 4.3 and 9.2 Hz, 1H), 7.18 – 7.10 (m, 1H), 4.20 – 4.14 (m, 2H), 1.21 (t, *J* = 7.2 Hz, 3H).

1-Amino-4-morpholinopyridazin-1-ium iodide (73)

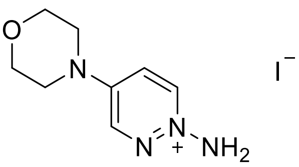

To amino hydrogen sulfate (1.40 g, 12.35 mmol) in water (10 mL) at 0 °C was added sodium hydrogen carbonate (1.037 g, 12.35 mmol) in water (10 mL) drop-wise and stirred for 10 min. The resulting solution was added drop-wise to 4-pyridazin-4-ylmorpholine (1.70 g, 10.29 mmol) in water (10 mL) and the mixture stirred for 5 h at 70 °C . Further amino hydrogen sulfate / $NaHCO_3$ aqueous solution (0.4 equiv) was added, and the mixture stirred overnight at 70 °C. After cooling to RT, Potassium iodide (2.05 g, 12.35 mmol) was added and stirred for 10 min. RM was filtered to remove solid, the water evaporated and MeOH added and evaporated to dryness. Residue was triturated from EtOH, and the solid stirred in MeOH, filtered and evaporated to give **73** (1.3 g, 3.80 mmol, 37%). ^{1}H NMR (DMSO-*d*$_6$): δ 8.86 (d, *J* = 3.7 Hz, 1H), 8.72 (d, *J* = 7.7 Hz, 1H), 7.76 (s, 2H), 7.46 (dd, *J* = 3.7, 7.7 Hz, 1H), 3.78 – 3.70 (m, 8H).

Ethyl 2-(2-fluoro-5-nitrophenyl)-5-morpholinopyrazolo[1,5-b]pyridazine-3-carboxylate (74)

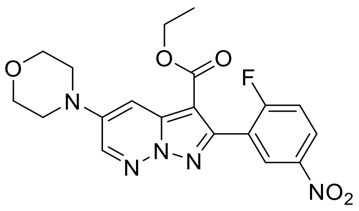

To **72** (0.512 g, 2.05 mmol) and **73** (0.758 g, 2.46 mmol) in MeCN (20 mL) at 0 °C was added 1,8-diazabicyclo[5.4.0]undec-7-ene (DBU) (0.687 g, 4.51 mmol) drop-wise and stirred at RT overnight. Solvent was evaporated and crude material chromatographed (0 – 80% EtOAc/heptane) to give **74** (0.245 g, 0.53 mmol, 26%). ^{1}H NMR ($CDCl_3$): δ 8.63 (dd, *J* = 2.8, 6.0 Hz, 1H), 8.39 – 8.34 (m, 1H), 8.28 (d, *J* = 5.5 Hz, 1H), 7.32 (t, *J* = 9.0 Hz, 0H), 6.56 (d, *J* = 5.5 Hz, 1H), 4.21 (q, *J* = 7.2 Hz, 2H),

3.95 (dd, J = 4.6, 4.6 Hz, 4H), 3.25 (dd, J = 4.5, 4.5 Hz, 4H), 1.11 (dd, J = 7.1, 7.1 Hz, 3H). m/z 416.2 $[M+H]^+$.

5-Bromo-1-methyl-pyrrolo[2,3-*b*]pyridine (76)

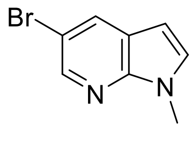

To a suspension of NaH (0.2639 g, 6.60 mmol) in THF (15 mL), cooled to 0 ^{0}C, 5-bromo-1*H*-pyrrolo[2,3-*b*]pyridine (1 g, 5.08 mmol) was added portion-wise. The RM was allowed to stir at 0 °C for 5 min then iodomethane (0.8645 g, 6.09 mmol) was added drop-wise. The RM was allowed to warm to RT, stirred for 5 h, cooled to 0 °C and quenched by careful addition of water. The RM was partitioned between water / EtOAc and the aqueous layer further extracted with EtOAc. The combined organics were washed with brine, dried over $MgSO_4$ and concentrated to give a brown oil which was purified by flash chromatography (0 – 50% EtOAc/heptane) to give **76** (0.785 g, 3.53 mmol, 70%). ^{1}H NMR ($CDCl_3$): δ 8.37 (d, J = 2.1 Hz, 1H), 8.04 (d, J = 2.1 Hz, 1H), 7.21 (d, J = 3.4 Hz, 1H), 6.42 (d, J = 3.4 Hz, 1H), 3.89 (s, 3H).

4-(1-Methylpyrrolo[2,3-*b*]pyridin-5-yl)morpholine (77)

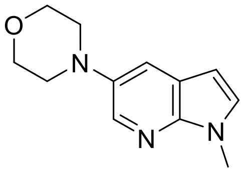

A microwave vial was charged with **76** (0.141 g, 0.67 mmol), NaO*t*Bu (0.096 g, 1.00 mmol), Pd2(dba)3 (0.0125 g, 0.01 mmol) and Xantphos (0.0233 g, 0.04 mmol). The vial was sealed and purged with nitrogen. Toluene (2 mL) was added followed by morpholine (0.07 g, 0.80

mmol) and the RM heated to 80 °C overnight. After cooling, solvent was evaporated and crude material purified by flash chromatography (0 – 100% EtOAc/heptane) to afford **77** (0.089 g, 0.39 mmol, 48%). ^{1}H NMR ($CDCl_3$): δ 8.19 (d, *J* = 2.6 Hz, 1H), 7.50 (d, *J* = 2.6 Hz, 1H), 7.16 (d, *J* = 3.4 Hz, 1H), 6.38 (d, *J* = 3.4 Hz, 1H), 3.95 – 3.92 (m, 4H), 3.88 (s, 3H), 3.16 - 3.13 (m, 4H).

_N_-(4-Fluoro-3-iodo-phenyl)pyrrolidine-1-carboxamide (78)

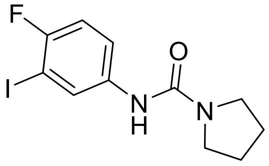

To a solution of 4-fluoro-3-iodo-aniline (0.43 g, 1.81 mmol) and DMAP (0.0111 g, 0.09 mmol) in pyridine (1 mL) / DCM (5 mL) at 0 °C was added pyrrolidine-1-carbonyl chloride (0.30 g, 2.18 mmol) drop-wise. The RM was allowed to warm to RT and heated to 50 °C overnight. After cooling, RM was partitioned between DCM and 1M HCl. The aqueous layer was further extracted with DCM (2 x 20 mL) and the combined organics washed with brine (20 mL), dried over $MgSO_4$ and concentrated to give a gum which was triturated with a 1:1 mixture of EtOAc:Et_2O to give **78** (0.524 g, 1.49 mmol, 82%). ^{1}H NMR ($CDCl_3$): δ 7.87 (dd, *J* = 2.7, 5.4 Hz, 1H), 7.39 (ddd, *J* = 2.7, 4.3, 8.9 Hz, 1H), 6.99 (dd, *J* = 7.5, 8.9 Hz, 1H), 6.11 (brs, 1H), 3.49 – 3.46 (m, 4H), 2.03 – 1.99 (m, 4H).

1,6-Diamino-4-morpholinopyridazin-1-ium (79)

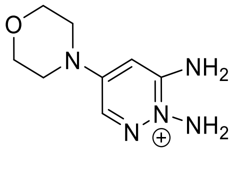

To 5-morpholinopyridazin-3-amine (6.12 g, 34.0 mmol) in DCM (113 mL) / MeOH (113 mL) at 0 °C was added *O*-(mesitylsulfonyl)hydroxylamine (8.04 g, 37.4 mmol) portion-wise and stirred for 10

min. Solvent was evaporated and Et_2O added and evaporated to give crude **79** (1:1 mix of isomers) which was used in the next step without purification. *m/z* 196.2 $[M + H]^+$.

2-Fluoro-5-(pyrrolidine-1-carboxamido)benzoic acid (80)

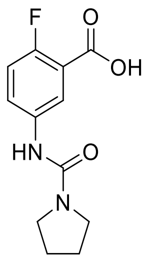

To methyl 5-amino-2-fluoro-benzoate (2.0 g, 11.8 mmol) in DCM (15 mL) / Pyridine (5 mL) was added pyrrolidine-1-carbonyl chloride (2.37 g, 17.7 mmol) drop-wise, followed by DMAP (0.145 g, 1.18 mmol) and stirred at 60 °C for 3 h. The RM was cooled, washed with water, 1M HCl and brine and the solvent evaporated. MeCN was added and the resulting solid collected, washed with further MeCN and dried to give crude ester which was used in the next step without purification (*m/z* 267.1 $[M + H]^+$). The crude ester (2.0 g,7.5 mmol) was taken up in NaOH (10 mL, 2M) / MeOH (5mL) and stirred at RT for 18 h. The mixture was concentrated to approx. 10 mL, acidified by addition of conc. HCl and the resulting solid collected, washed with water and dried to give **80** (1.99 g, 7.5 mmol, 64 % over 2 steps). ^1H NMR (DMSO-d_6): δ 13.11 (s, 1H), 8.33 (s, 1H), 8.05 – 8.02 (m, 1H), 7.81 – 7.75 (m, 1H), 7.16 (dd, J = 9.9, 9.7 Hz, 1H), 3.39 – 3.34 (m, 4H, under water signal), 1.88 – 1.83 (m, 4H); *m/z* 253.1 $[M + H]^+$.

5-Bromo-2-hydrazinylpyrimidine (82)

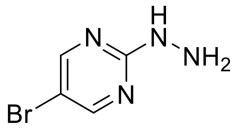

To 5-bromo-2-chloropyrimidine (10 g, 51.7 mmol) in MeOH (150 mL), hydrazine monohydrate (9.30 mL, 191 mmol) was added and the solution stirred at 80 °C overnight. The mixture was cooled to RT and the resulting solid collected, washed with MeOH (200 mL) and dried under vacuum to afford **82** (9.7 g, 51.1 mmol, 99%).^{1}H NMR (DMSO-d_6): δ 8.46 (bs, 1H), 8.39 (s, 2H), 4.17 – 4.22 (bs, 2H).

(E)-5-Bromo-2-(2-(2-fluoro-5-nitrobenzylidene)hydrazinyl)pyrimidine (83)

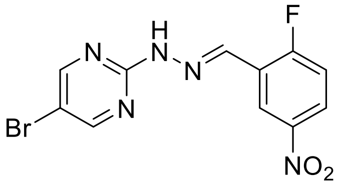

To a suspension of **82** (9.6 g, 50.8 mmol) in EtOH (150 mL), 2-fluoro-5-nitrobenzaldehyde (9.02 g, 53.3 mmol) was added and the mixture stirred at RT overnight. Solvent was evaporated and the residue triturated with Et_2O (2x100 mL). The resulting solid was collected and dried to yield **83** (16.0 g, 47.0 mmol, 93%). ^{1}H NMR (DMSO-d_6): δ 11.88 (bs, 1H), 8.69 – 8.65 (m, 3H), 8.37 (s, 1H), 8.23 – 8.30 (m, 1H), 7.58 (t, J = 9.5 Hz, 1H)

6-Bromo-3-(2-fluoro-5-nitrophenyl)-[1,2,4]triazolo[4,3-a]pyrimidine (84)

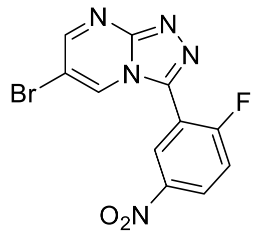

To a suspension of **83** (16 g, 49.7 mmol) in DCM (350 mL), Iodobenzene diacetate (16 g, 49.7 mmol) was added and the mixture stirred at RT overnight. Solvent was evaporated and the residue triturated with Et_2O (3x100 mL) to afford **84** (13.9 g, 43.4 mmol, 87%). ^{1}H NMR (DMSO-d_6): δ 9.29 (t, J = 2.2 Hz, 1H), 8.82 (d, J = 2.3 Hz, 1H), 8.62 (dd, J = 5.9, 2.9 Hz, 1H), 8.48 (ddd, J = 9.2, 4.3 and 2.9 Hz, 1H), 7.72 (t, J = 9.2 Hz, 1H).

6-Bromo-2-(2-fluoro-5-nitrophenyl)-[1,2,4]triazolo[1,5-a]pyrimidine (85)

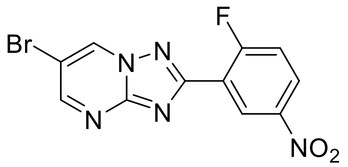

A solution of **84** (13.88 g, 41.1 mmol) in formic acid (150 mL) was heated to reflux for 5 h. Solvent was evaporated and the resulting solid washed with Et_2O (100 mL), collected and dried to yield **85** (12.5 g, 36.8 mmol, 90%). ^{1}H NMR (DMSO-d_6): δ 10.04 (d, J = 2.3 Hz, 1H), 9.08 (d, J = 2.3 Hz, 1H), 9.01 (dd, J = 6.3 and 3.0 Hz, 1H), 8.45 – 8.52 (m, 1 H), 7.77 (dd, J = 9.9 and 9.2 Hz, 1H).

3-(6-Bromo-[1,2,4]triazolo[1,5-a]pyrimidin-2-yl)-4-fluoroaniline (86)

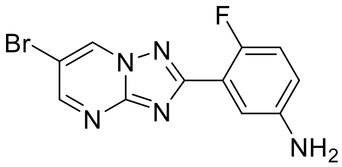

To a suspension of **85** (12.45 g, 36.8 mmol) in EtOH (150 mL) / THF (300 mL) was added iron (16.45 g, 295 mmol) and a solution of ammonium chloride (7.88 g, 147 mmol) in water (150 mL) and stirred at 75 °C overnight. The hot mixture was filtered through celite and washed through with hot MeOH (10 L). The filtrate was diluted with DCM (12 L) and washed with water (12 L). The organic layer was dried over Na_2SO_4, filtered and concentrated to afford **86** (9.0 g, 29.2 mmol, 79%). ^{1}H NMR (DMSO-d_6): δ 9.92 (d, J = 2.5 Hz, 1H), 8.98 (d, J = 2.5 Hz, 1H), 7.42 (dd, J = 6.2 and 2.9 Hz, 1H), 7.05 (dd, J = 10.9 and 8.8 Hz, 1H), 6.76 – 6.68 (m, 1H), 5.23 (s, 2H).

***N*-(4-Fluoro-3-(imidazo[1,2-*a*]pyrimidin-7-yl)phenyl)pyrrolidine-1-carboxamide (88)**

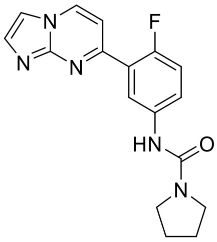

To **87** (0.5 g, 1.66 mmol) in EtOH (5 mL) was added 2-bromo-1,1-dimethoxy-ethane (0.56 g, 3.32 mmol) and hydrobromic acid (0.805 g, 9.96 mmol) and the RM stirred at 80 °C for 9 h. Solvent was evaporated, and the RM partitioned between sat. aq. $NaHCO_3$ / DCM. The organics were evaporated and purified by flash chromatography (0 – 20 % MeOH in DCM) to give crude **88** which was used without purification. m/z = 326.3 $[M+H]^+$

***N*-(3-(3-Bromoimidazo[1,2-*a*]pyrimidin-7-yl)-4-fluorophenyl)pyrrolidine-1-carboxamide (89)**

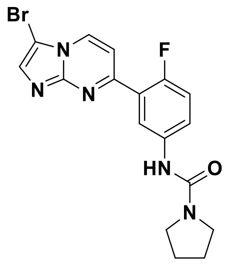

A mixture of **88** (1.0 g, 3.07 mmol) and sodium acetate (0.504 g, 6.14 mmol) in MeOH (4 mL, saturated with KBr) was cooled to -10 °C and bromine (0.491 g, 3.07 mmol) added drop-wise over 5 min. The RM was quenched by addition of 1M sodium sulfite solution (2 mL) and the solvent evaporated. The residue was taken up in water (15 mL) / sat. aq. $NaHCO_3$ (15 ml) and extracted with EtOAc (2 x 50 mL). The combined organics were washed with brine (40 mL), dried over Na_2SO_4 and evaporated to give **89** as an off-white solid (0.527 g, 1.04 mmol, 34 %). ^{1}H NMR (DMSO-d_6): δ 8.88 (d, J = 7.3 Hz, 1H), 8.44 (br s, 1H), 8.25 (dd, J = 7.1, 2.8 Hz, 1H), 7.97 (s, 1H), 7.83 – 7.79 (m, 1H), 7.59 (dd, J = 7.3, 1.8 Hz, 1H), 7.28 (dd, J = 11.3, 9.1 Hz, 1H), 3.41 – 3.36 (m, 4H), 1.88 – 1.84 (m, 4H). m/z = 404.1, 406.1 $[M+H]^+$

3-(7-(2-Fluoro-5-nitrophenyl)imidazo[1,2-*a*]pyrimidin-3-yl)morpholine (93)

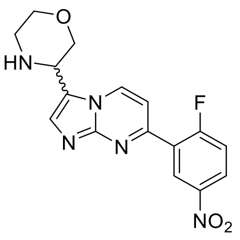

To DMF (6 mL) at 0 ºC was added $POCl_3$ (1.96 g, 12.78 mmol) dropwise. After stirring at 0 ºC for 1 h, **90** (1.5 g, 5.81 mmol) was added and the RM stirred at 80 ºC for 20 h. Solvent was evaporated and crude product quenched by dropwise addition to ice/water then purified by flash chromatography (3 % MeOH / DCM) to yield crude **92** (0.512 g, 1.8 mmol). This was taken up in DCM (5 mL), 2-

[(tributylstannyl)methoxy]-ethanamine (0.636 g, 1.75 mmol, SnAP-M reagent) and 4Å MS (0.65 g) added and the mixture stirred at RT for 15 h. Separately, 2,6 lutidine (0.375 g, 3.5 mmol) was added to a suspension of hexafluoro-2-propanol (3 mL) and $Cu(OTf)_2$ (1.27 g, 3.5 mmol) and stirred at 50 ºC for 3 h. This was added in one portion to the RM and stirred at RT for 18 h. Solvent was evaporated and the crude product purified by flash chromatography (3 % MeOH / DCM) to provide **93** (0.3 g, 0.87 mmol, 50%) which was used without further purification. m/z = 344.0 $[M+H]^+$

***tert*-Butyl 3-(7-(2-fluoro-5-(pyrrolidine-1-carboxamido)phenyl)imidazo[1,2-*a*]pyrimidin-3-yl)morpholine-4-carboxylate (95)**

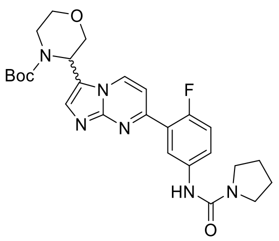

A mixture of **93** (0.3 g, 0.87 mmol) and di-tert-butyl dicarbonate (0.381 g, 1.75 mmol) in MeOH (3 mL) was stirred at 50 ºC for 3 h. Solvent was evaporated and crude material chromatographed (3% MeOH / DCM) to give **94** (0.24 g, 0.54 mmol, 62%). m/z = 444.0 $[M+H]^+$. A mixture of **94**, iron (0.242 g, 4.33 mmol) and NH_4Cl (0.116 g, 2.16 mmol) in 4:1 $EtOH/H_2O$ (2.5 mL) was stirred at 75 ºC for 3 h. The RM was filtered and solvent evaporated to give crude aniline which was dissolved in 1:4 pyridine/DCM (2.5 mL). Pyrrolidine-1-carbonyl chloride (0.071 g, 0.53 mmol) and DMAP (0.0024 g, 0.02 mmol) were added and stirred at 50 ºC for 20 h. After cooling, the solvent was evaporated and the crude material chromatographed to give **95** (0.08 g, 0.19 mmol, 36%) which was used without further purification. m/z = 511.8 $[M+H]^+$

4,4'-(1-(1*H*-Benzo[*d*][1,2,3]triazol-1-yl)-2-(indolin-1-yl)ethane-1,2-diyl)dimorpholine (96a)

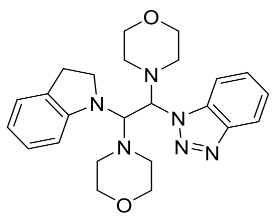

The reaction was carried out in two batches. To a mixture of benzotriazole (200 g, 1.68 mol) in EtOH (2 L) was added morpholine (146 g, 1.68 mol) at 15 °C. After stirring for 30 min, 40% aq. glyoxal in water (121.8 g, 0.84 mol) was added at 15 °C and the mixture stirred for 12 h during which time a precipitate formed. The solid was filtered and the batches combined and washed with EtOH (3 x 500 mL) to give **96a** (610 g, 1.4 mol, 83%) as a white solid. ^{1}H NMR ($CDCl_3$): δ 8.20 – 8.15 (m, 2H), 7.7 – 7.5 (m, 4H), 7.5 – 7.35 (m, 2H), 6.6 (br s, 2H), 3.35 – 3.2 (m, 4H), 3.15 – 2.9 (m, 4H), 2.7 – 2.5 (m, 8H).

Benzyl (*R*)-(4-fluoro-3-(2-((2-(3-methylmorpholino)-2-oxoethyl)amino)pyrimidin-4-yl)phenyl)carbamate (99a)

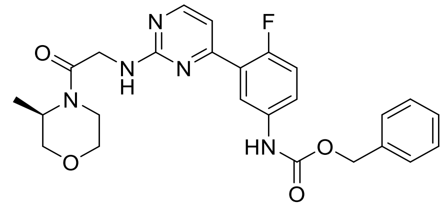

To a solution of **98a** (0.464 g, 2.93 mmol) in 1,4-dioxane (13 mL) was added **97** (0.7 g, 1.957 mmol) and DIPEA (0.513 mL, 2.93 mmol) and the resulting solution stirred at 120 ºC overnight in a sealed tube. After cooling, the RM was partitioned between DCM and water. The layers were separated, and the aqueous layer extracted with DCM (2x 10 mL). The combined organics were dried over anh. Na_2SO_4, filtered and concentrated and the crude material was purified by flash chromatography (0 – 50 % EtOAc:EtOH (3.1) / cyclohexane) to give **99a** (0.53 g, 1.64 mmol, 56%). ^{1}H NMR (DMSO-d_6):

δ 9.93 (bs, 1H), 8.38 (d, J = 5.1 Hz, 1H), 8.19 (bs, 1H), 7.50 (bs, 1H), 7.46 – 7.33 (m, 5H), 7.28 (dd, J = 8.7 and 11.0 Hz, 1H), 7.11 (s, 1H), 7.01 – 6.98 (m, 1H), 5.19 (s, 2H), 4.36 – 3.95 (m, 4H), 3.85 – 3.80 (m, 1H), 3.64 – 3.34 (m, 4H), 1.35 – 1.06 (m, 3H); m/z = 480.1 $[M+H]^+$

Benzyl (S)-(4-fluoro-3-(2-((2-(3-methylmorpholino)-2-oxoethyl)amino)pyrimidin-4-yl)phenyl)carbamate (99b)

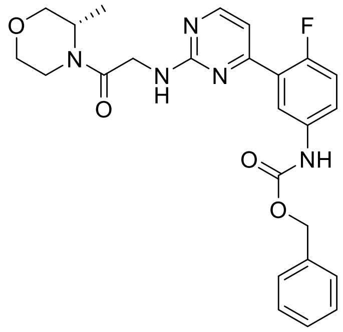

A suspension of **97** (0.7 g, 1.957 mmol), **98b** (0.464 g, 2.93 mmol) and DIPEA (0.513 mL, 2.93 mmol) in 1,4-dioxane (2 mL) was stirred at 120 ºC overnight in a sealed tube. The reaction mixture was poured into water (100 mL) and extracted with DCM (100 mL). The organic layer was dried over anh. Na_2SO_4, filtered and concentrated to dryness. The crude residue was purified by flash cromatography on SiO_2 (0-50% EtOH:EtOAc1:3/cyclohexane) to afford **99b** (0.62 g, 1.29 mmol, 66%). ^1H NMR (DMSO-d_6): δ 9.93 (bs, 1H), 8.38 (d, J= 5.1 Hz, 1H), 8.19 (bs, 1H), 7.51 (bs, 1H), 7.46 – 7.34 (m, 5H), 7.27 (dd, J = 9 and 11 Hz, 1H), 7.12 (bs, 1H), 7.0 – 6.98 (m, 1H), 5.18 (s, 2H), 4.35 – 4.01 (m, 4H), 3.85 – 3.79 (m, 1H), 3.64 – 3.55 (m, 2H), 3.55- 3.45 (b, 1H), 3.44- 3.35 (b, 1H), 1.37 – 1.05 (m, 3H); m/z = 480.0 $[M+H]^+$

Benzyl (4-fluoro-3-(2-((2-oxo-2-(2-oxa-7-azaspiro[4.4]nonan-7-yl)ethyl)amino)pyrimidin-4-yl)phenyl)carbamate (99c)

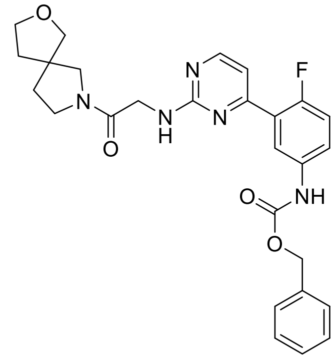

A suspension of **97** (0.5 g, 1.398 mmol), **98c** hydrochloride (0.463 g, 2.096 mmol) and DIPEA (0.732 mL, 4.19 mmol) in 1,4-dioxane (10 mL) was stirred at 120 ºC overnight in a sealed tube. The reaction was poured into water (50 mL) and extracted with DCM (50 mL). The organic layer was dried over anh. Na_2SO_4, filtered and concentrated. The crude residue was purified by flash cromatography on SiO_2 (0-40% EtOH:EtOAc1:3/cyclohexanes) to afford **99c** (0.38 g, 0.75 mmol, 54%). ^{1}H NMR (DMSO-d_6): δ 9.94 (bs, 1H), 8.38 (dd, *J*= 1.5 and 5.1 Hz, 1H), 8.17 (bs, 1H), 7.52 (bs, 1H), 7.46 – 7.34 (m, 5H), 7.27 (dd, *J* = 9 and 11 Hz, 1H), 7.15 (bs, 1H), 7.01 – 6.98 (m, 1H), 5.17 (s, 2H), 4.11 – 4.01 (m, 2H), 3.76 (bs, 1H), 3.67 – 3.48 (m, 4H), 3.44- 3.35 (m, 2H), 3.30 (bs, 1H), 2.68 (dt, *J*= 1.8, 1.8 and 3.7 Hz, 1H), 1.95 – 1.75 (m, 3H); m/z = 506.0 $[M+H]^+$

Benzyl (4-fluoro-3-(2-((2-(isopropylamino)-2-oxoethyl)amino)pyrimidin-4-yl)phenyl)carbamate (99d)

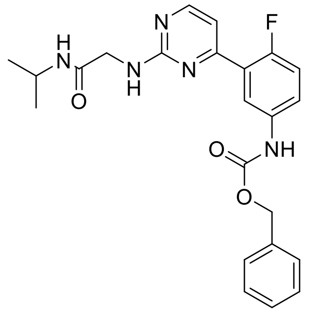

To a solution of **97** (0.600 g, 1.677 mmol) in 1,4-dioxane (11.18 ml), **98d** (0.292 g, 2.52 mmol) and DIPEA (0.439 ml, 2.52 mmol) were added. The resulting solution was stirred at 120 ºC for 45h. The reaction was partitioned between DCM and water. The layers were separated and the aqueous layer was extracted with DCM (2x10 mL). The organic layers were combined, dried with anh. Na_2SO_4, filtered and concentrated to give a brown solid, which was suspended in MeOH / DCM and absorbed onto silica. The resulting mixture was added to a Merck 50 g and eluted with 0-50% ethyl acetate:ethanol(3:1)/cyclohexane to give **99d** (0.35 g, 0.80 mmol, 48%). ^{1}H NMR (DMSO-d_6): δ 9.90 (bs, 1H), 8.37 (d, *J*= 5.0 Hz, 1H), 8.11 (dd, *J*= 2.6 and 6.7 Hz, 1H), 7.66 (bd, *J*= 8.1 Hz, 1H), 7.63-7.54 (b, 1H), 7.45 – 7.24 (m, 7H), 6.99 (dd, *J* = 2.3 and 5.0 Hz, 1H), 5.17 (s, 2H), 3.93- 3.79 (m, 3H), 1.09 – 0.98 (bd, *J*= 6.3 Hz, 6H); m/z = 438.2 $[M+H]^+$

Benzyl (4-fluoro-3-(2-((2-((1-methoxypropan-2-yl)amino)-2-oxoethyl)amino)pyrimidin-4-yl)phenyl)carbamate (99e)

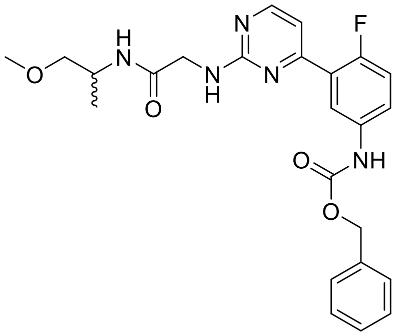

To a solution of **97** (0.600 g, 1.677 mmol) in 1,4-dioxane (11.18 ml), **98e** (0.368 g, 2.52 mmol) and DIPEA (0.439 ml, 2.52 mmol) were added. The resulting solution was stirred at 120 ºC for 35 h. The reaction was partitioned between DCM and water. The layers were separated and the aqueous layer was extracted with DCM (2x10 mL). The organic layers were combined, dried with anh. Na_2SO_4, filtered and concentrated to give a brown oil, which was dissolved in DCM and purified by flash chromatography (0-50% ethyl acetate:ethanol(3:1)/cyclohexane) to give **99e** (0.38g, 0.80 mmol, 48%). ^{1}H NMR (DMSO-d_6): δ 9.90 (bs, 1H), 8.37 (d, *J*= 5.0 Hz, 1H), 8.11 (dd, *J*= 2.6 and 6.7 Hz, 1H), 7.67 (bd, *J*= 7.8 Hz, 1H), 7.66- 7.55 (bs, 1H), 7.44 – 7.25 (m, 7H), 6.99 (dd, *J* = 2.5 and 5.0 Hz, 1H), 5.17 (s, 2H), 3.97- 3.85 (m, 3H), 3.25- 3.1 (m, 5H), 1.06 – 0.98 (m, 3H); m/z = 468.2 [M+H]$^+$

Benzyl (*R*)-(4-fluoro-3-(3-(3-methylmorpholino)imidazo[1,2-*a*]pyrimidin-7-yl)phenyl)carbamate (100a)

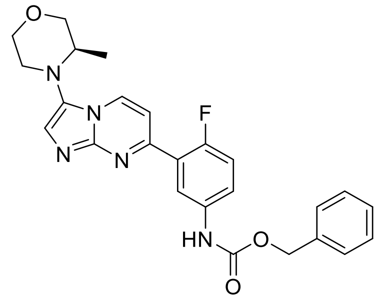

A solution of **99a** (0.53 g, 1.105 mmol) in $POCl_3$ (3.39 mL, 36.5 mmol) was stirred at 80 ºC for 10.5 h, then at RT for 2 days. Solvent was evaporated and further DCM (2x 10 mL) added and evaporated. The crude material was partitioned between DCM / 2M NaOH. The layers were separated and the aqueous phase further extracted with DCM (3x 10 mL). The combined organics were dried over Na_2SO_4, filtered and concentrated and the crude material purified by flash chromatography (0 – 30 % EtOAc:EtOH (3:1) / cyclohexane) to give **100a** (0.343 g, 0.74 mmol, 67% crude yield) which was used without further purification. m/z = 462.2 $[M+H]^+$.

Benzyl (S)-(4-fluoro-3-(3-(3-methylmorpholino)imidazo[1,2-a]pyrimidin-7-yl)phenyl)carbamate (100b)

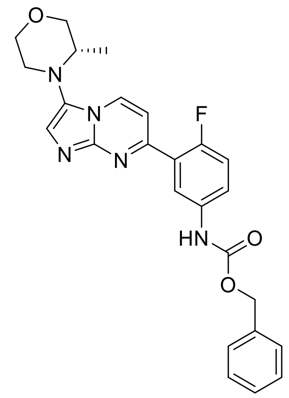

A solution of **99b** (0.58 g, 1.21 mmol) in $POCl_3$ (5 mL, 53.8 mmol) was stirred at 80 ºC for 5 h, then at RT for 2 days. The reaction mixture was concentrated to dryness and the resulting crude was dissolved in DCM (50 mL) and washed with water (30 mL) and $NaHCO_3$ (saturated aqueous solution 20 mL). The organic layer was dried over anh. Na_2SO_4, filtered and concentrated to afford **100b** (0.37 g, 0.80 mmol, 66% crude yield) which was used without further purification. m/z = 462.2 $[M+H]^+$.

Benzyl (3-(3-(2-oxa-7-azaspiro[4.4]nonan-7-yl)imidazo[1,2-a]pyrimidin-7-yl)-4-fluorophenyl)carbamate (100c).

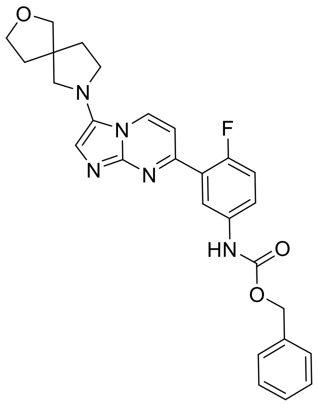

A solution of **99c** (0.38 g, 0.75 mmol) in $POCl_3$ (5 mL, 53.8 mmol) was stirred at 80 ºC for 24 h. The reaction mixture was concentrated to dryness and the resulting crude was dissolved in DCM (3x10 mL) and concentrated to dryness. The crude was disolved in MeOH (10 mL) and NH_3 (7N in MeOH, 1 mL) was added and the solvents were removed by concentration to give **100c** (0.283 g, 0.58 mmol, 77% crude yield) which was used without further purification. m/z = 488.1 $[M+H]^+$.

Benzyl (4-fluoro-3-(3-(isopropylamino)imidazo[1,2-a]pyrimidin-7-yl)phenyl)carbamate (100d).

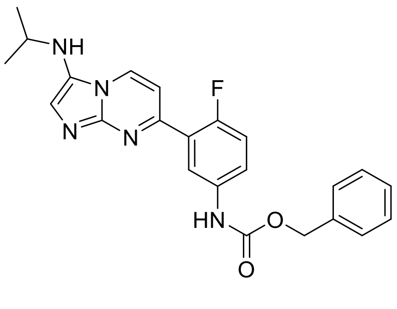

A solution of **99d** (0.35 g, 0.8 mmol) in $POCl_3$ (5.5 mL, 59.2 mmol) was stirred at RT for 6 days. The solvents were evaporated in vacuo and redissolved in dichloromethane (2x10 mL). The crude was partitioned between dichlromethane and an aqueous solution of NaOH 2M. The layers were separated and the aqueous phase was extracted with dichloromethane (3x10 mL) The organic layers were

combined, dried with anh. Na_2SO_4, filtered and concentrated to give **100d** (0.272 g, 81%) which was used without further purification. m/z = 420.2 $[M+H]^+$.

Benzyl (4-fluoro-3-(3-((1-methoxypropan-2-yl)amino)imidazo[1,2-a]pyrimidin-7-yl)phenyl)carbamate (100e).

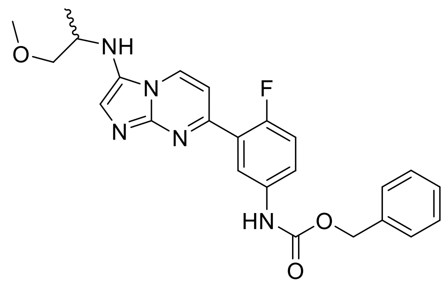

A solution of **99e** (0.38 g, 0.8 mmol) in $POCl_3$ (3.5 mL, 37.7 mmol) was stirred at RT for 43 h. The solvents were evaporated in vacuo and redissolved in DCM (2x10 mL). The crude was partitioned between DCM and an aqueous solution of NaOH 2M. The layers were separated and the aqueous phase was extracted with DCM (3x10 mL) The organic layers were combined, dried with anh. Na_2SO_4, filtered and concentrated to give a brown foam which was purified by flash chromatography (0-30% ethyl acetate:ethanol(3:1)/cyclohexane) to give **100e** (0.24 g, 0.53 mmol, 66% crude yield). ^{1}H NMR (DMSO-d$_6$): δ 9.97 (bs, 1H), 8.63 (d, *J*= 7.1 Hz, 1H), 8.17 (dd, *J*= 3.0 and 7.1 Hz, 1H), 7.63- 7.55 (m, 2H), 7.49- 7.26 (m, 6H), 7.22 (s, 1H), 5.35 (d, *J*= 7.1 Hz, 1H), 5.18 (s, 2H), 3.58- 3.29 (m, 6unesH), 1.18 (d, *J*= 6.6 Hz, 3H); m/z = 450.2 $[M+H]^+$

In vitro assays

Intramacrophage *L. donovani Assay*. This assay was conducted as previously described, except for compound exposure time, which was 96 h instead of 72 h.[4]

Intrinsic Clearance. This assay was conducted as previously described.[5]

Aqueous Solubility. This assay was conducted as previously described.[6]

FaSSIF Solublity. This assay was conducted as previously described.[5,7]

***In vivo* Mouse Efficacy Studies**. In vivo studies were carried out as previously described, with minor modifications.[7] Briefly: Control drug used was Miltefosine (30 mg/kg orally, twice daily for 5 days), vehicle used was 10% (v/v) dimethyl sulfoxide (DMSO), 40% polyethylene glycol 400, and 50% deionized water (10 Ml/kg orally, twice daily for 5 days) and mice were treated with test compound intraperitoneally (50 mg/kg twice daily for 5 days). [5]

***In vivo* pharmacokinetics**

All regulated procedures on living animals were carried out under the authority of a project license issued by the Home Office under the Animals (Scientific Procedures) Act 1986, as amended in 2012 (and in compliance with EU Directive EU/2010/63). Infected animals had access to food and water ad libitum and were housed under a 12 hour light/dark photoperiod. Animals used in these experiments were female NMRI and Balb/c mice (Harlan, UK).

Test compound DDD01008714 was dosed orally (P.O.) at 10 mg free base/kg (dose volume 10 mL/kg; dose vehicle: 10% (vol/vol) dimethyl sulfoxide (DMSO), 40% polyethylene glycol 400 (PEG400) and 50% deionized water) to female NMRI mice (n = 3) or intravenously (I.V.) at 3 mg free base/kg (dose volume 5 mL/kg; dose vehicle: 10% (vol/vol) dimethyl sulfoxide (DMSO) and 90% Saline) to female Balb/c mice (n = 3). Blood samples (10 µL) where taken from the tail vein of each mouse at 0.03, 0.08, 0.25, 0.5, 1, 2, 4, 6, 8, 24 hours post dose (0.03 and 24 hours post dose

samples for I.V. leg only) and mixed with two volumes of deionized water (20 µL). After suitable sample preparation, the concentration of test compound in the whole blood from treated mice was determined by UPLC-MS/MS using a Quattro Premier XE (Waters, USA). Pharmacokinetic parameters were derived from the mean blood concentration time curve using PK solutions software v 2.0 (Summit Research Services, USA).

Pharmacokinetics of 4

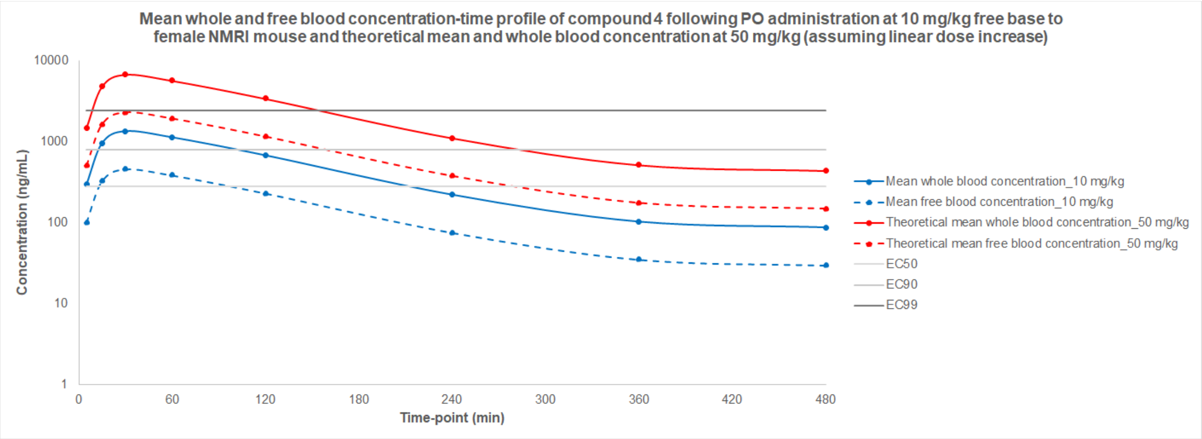

Plot of concentration vs. Time for **4**, dosed at 10 mg/kg. Extrapolated to 50 mg/kg assuming a linear dose increase, showing potential coverage above EC_{90} for approximately 6 hours

Modelling

Structure preparation

The homology model of the *L. donovani* 20S proteasome β4-β5 subunits (generated from the *L. tarantolae* cryo-EM structure in complex with compound **1)**[2] was used as a template to model the other compounds studied. . The complexes were prepared either by docking (using the Glide module of the Schrodinger platform) [9], or by modifying the central core of **1** in place. Atom position is crystal structures and models especially, can have certain errors associated with them (some of which include missing atoms, connectivity issues and incorrect bond orders, steric clashes between protein and the ligand, etc.) which translate into large errors in energy terms. To ensure the best possible starting point for QM calculations, the structures were subjected to a restrained minimisation procedure with the OPLS3e force field, as implemented in the Schrödinger platform,[9] where each heavy atom was allowed to deviate by up to 1 Å from its original position in crystal structure or model.

FMO calculation protocol

In recent years, significant progress has been made in approaches that use mainly QM to estimate binding.[8-10] FMO (Fragment Molecular Orbital) method is a general quantum- mechanical approach in which a large biological system is divided into smaller fragments. Residues within 5 Å from the ligand atoms were included in the FMO calculations, C-terminal N-methylated, N-terminal acetylated while maintaining the geometry of the neighbouring residue. Some residues are removed/added depending on the local substructure to ensure minimal disruption in the backbone chain. Fragmentation was done using Facio,[11,12] according to the well-established strategy where each FMO fragment is defined by the side chain, the Cα and backbone NH of a given aminoacid plus the carbonyl group of the adjacent aminoacid and the ligand was treated as one fragment. The calculations were

performed at MP2/6-31G* theory level, using GAMESS implementation.[13,14] The analysis of the results was performed using in-house tools developed in Python 3.[15]

Electrostatic surface potentials were generated using Jaguar[16] at the M06 level of theory with the 6-31+G** basis set. Electrostatic surface of the protein was generated using APBS plugin for PyMol.[17]

Molecular ESP maps for compounds 1,15, 18 – 27

Protein surfaces were generated using APBS plugin for PyMol and coloured by calculated charges (red -5kbT/ec, blue +5kbT/ec). The ligands surfaces were generated using Jaguar in Schrödinger and coloured by electrostatic potential (red -80 kcal/mol, blue +75 kcal/mol).

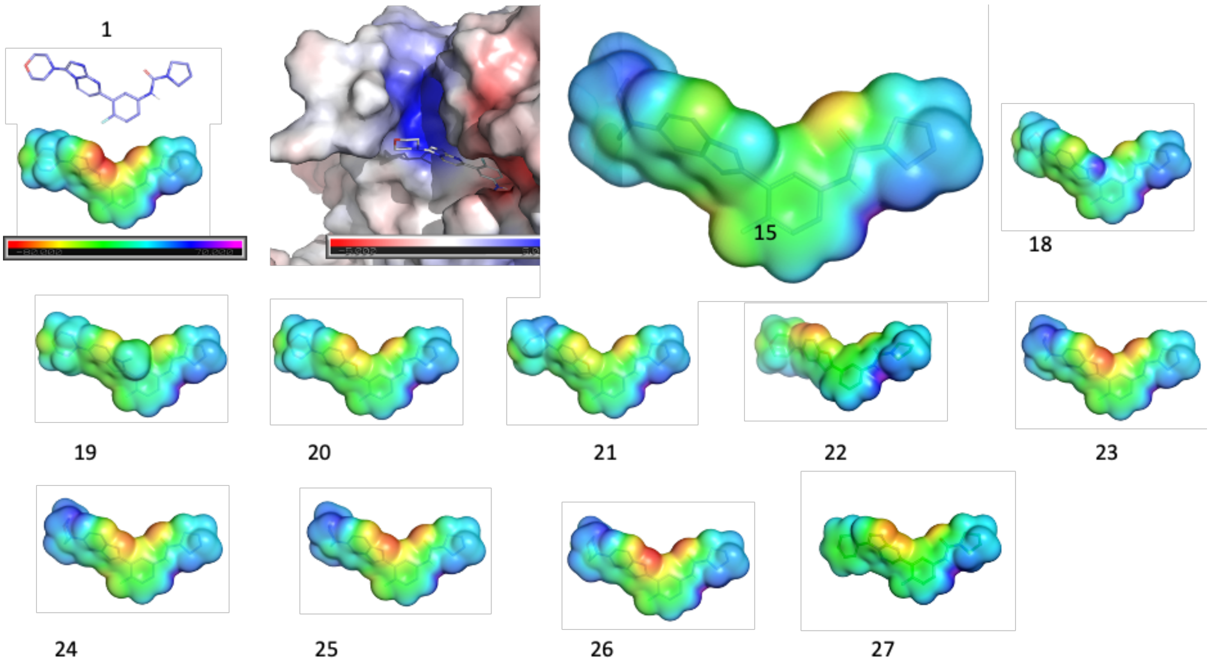

Supporting Information

HPLC Traces of Key Compounds

Compound 5

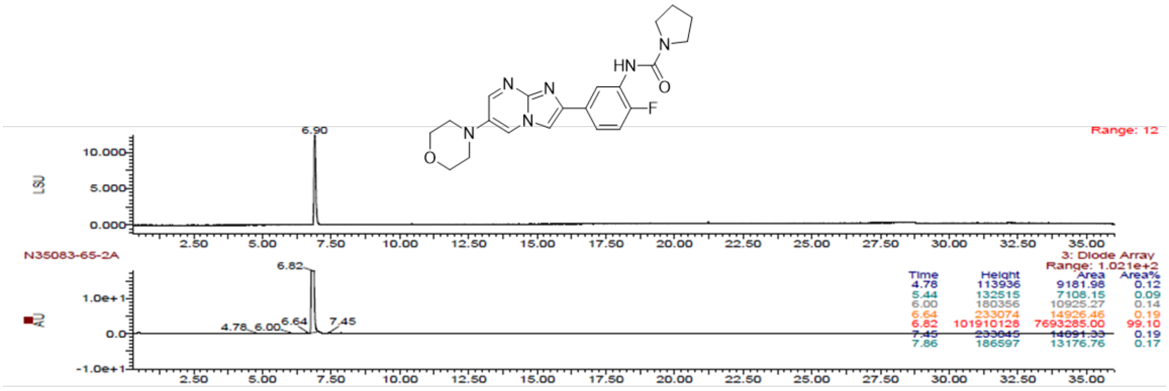

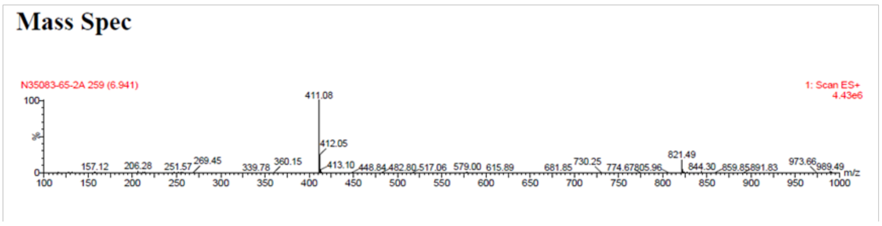

Compound 6

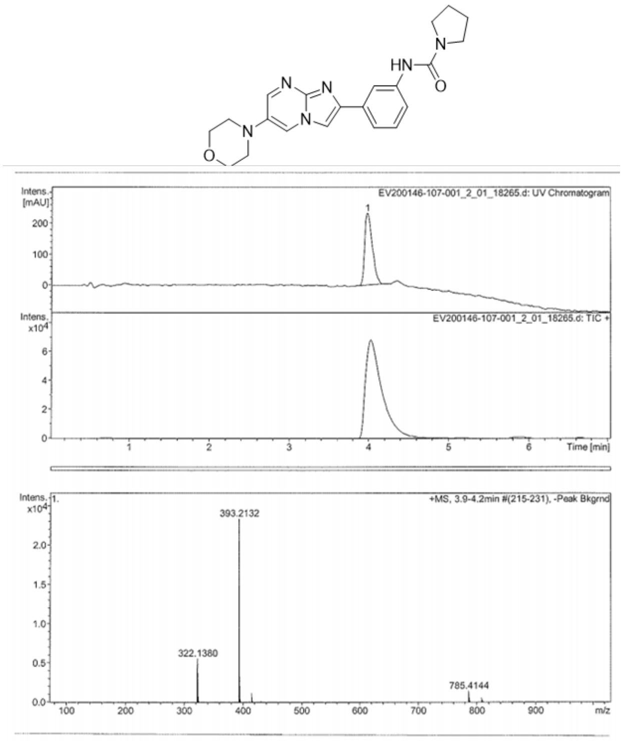

#	RT [min]	Area	Area Frac. %
1	4.0	1555.2	100.0

Compound 7

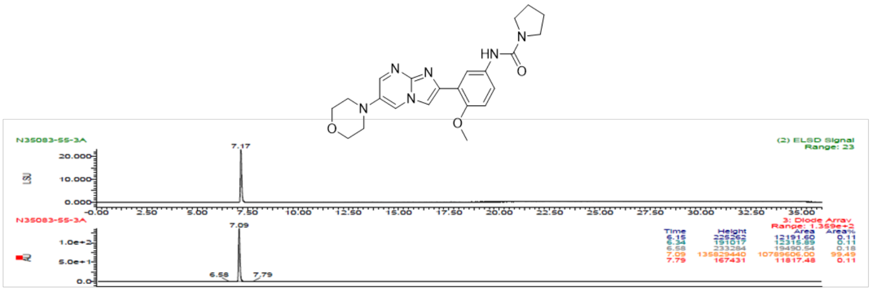

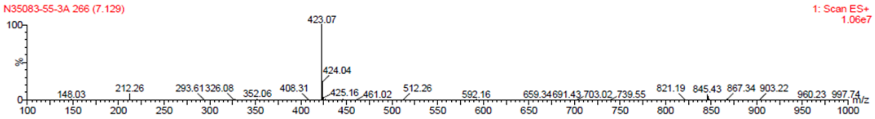

Compound 8

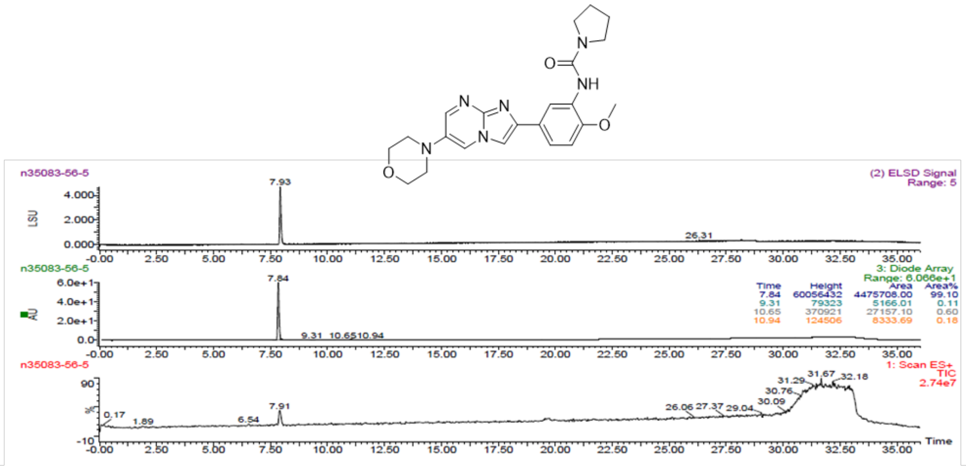

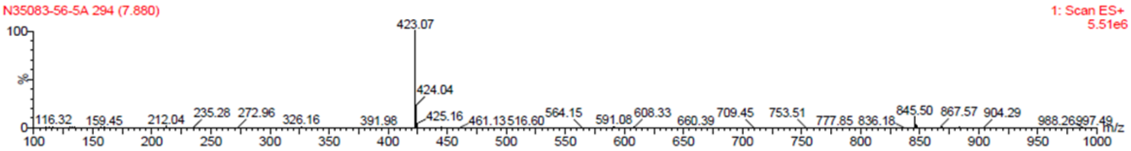

Compound 9

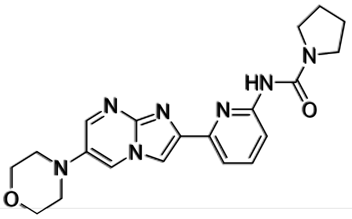

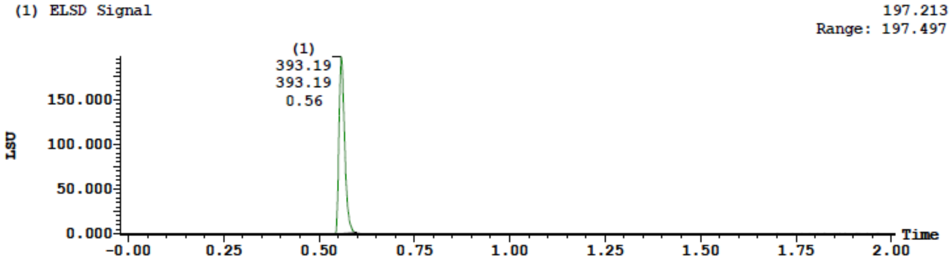

Peak Number	Compound	Time	AreaAbs	Area %Total	Width	Height	Mass Found
1	Found	0.56	3e+003	100.00	0	2e+005	393.19, 393.19

Peak ID	Compound	Time	Mass Found
1	Found	0.56	393.19

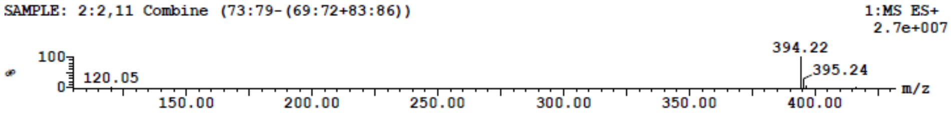

Compound 10

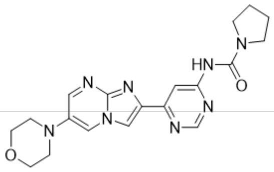

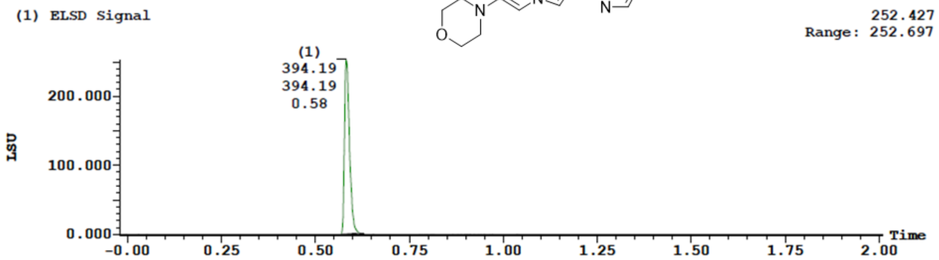

Peak Number	Compound	Time	AreaAbs	Area %Total	Width	Height	Mass Found
1	Found	0.58	4e+003	100.00	0	3e+005	394.19, 394.19

Peak ID	Compound	Time	Mass Found
1	Found	0.58	394.19

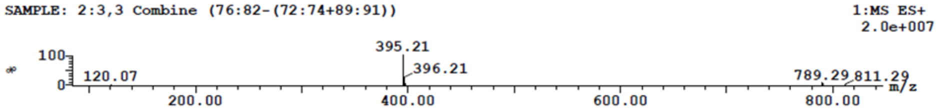

Compound 11

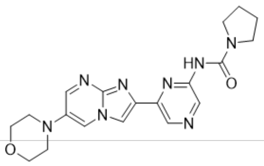

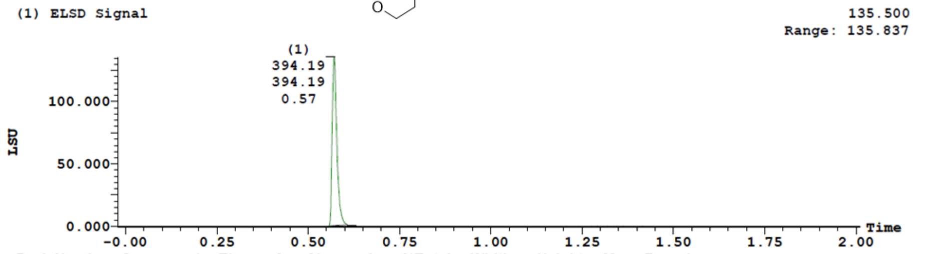

Peak Number	Compound	Time	AreaAbs	Area %Total	Width	Height	Mass Found
1	Found	0.57	2e+003	100.00	0	1e+005	394.19, 394.19

Peak ID	Compound	Time	Mass Found
1	Found	0.57	394.19

SAMPLE: 2:2,16 Combine (75:81-(70:72+89:91))

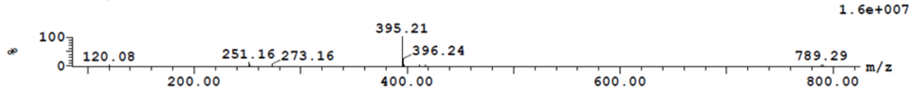

Compound 12

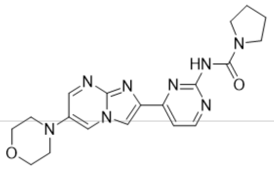

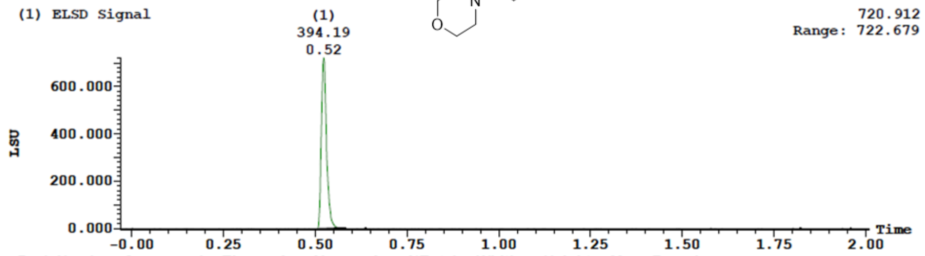

Peak Number	Compound	Time	AreaAbs	Area %Total	Width	Height	Mass Found
1	Found	0.52	1e+004	100.00	0	7e+005	394.19

Peak ID	Compound	Time	Mass Found
1	Found	0.52	394.19

SAMPLE: 2:2,7 Combine (69:75-(40:42+104:107))

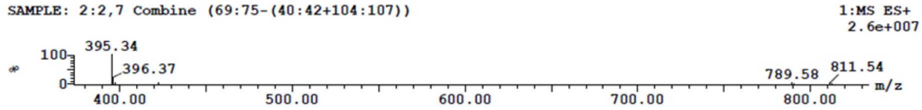

Compound 13

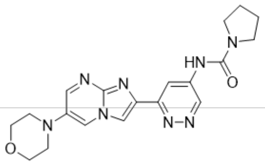

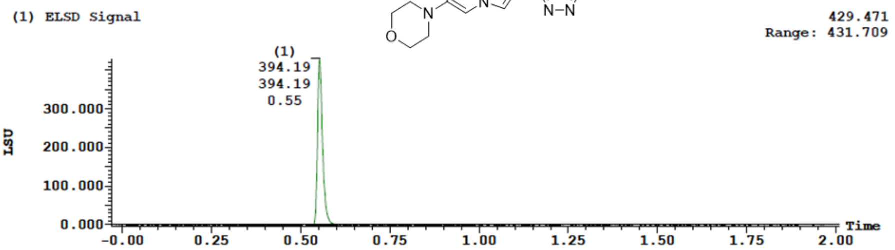

Peak Number	Compound	Time	AreaAbs	Area %Total	Width	Height	Mass Found
1	Found	0.55	7e+003	100.00	0	4e+005	394.19, 394.19

Peak ID	Compound	Time	Mass Found
1	Found	0.55	394.19

SAMPLE: 2:1,6 Combine (73:79-(43:46+110:113))

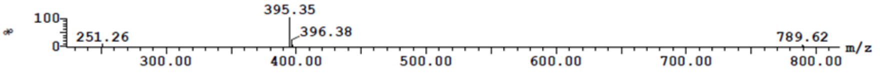

Compound 15

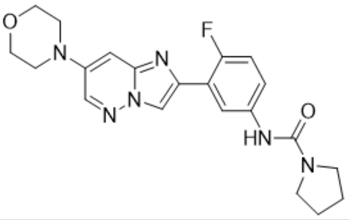

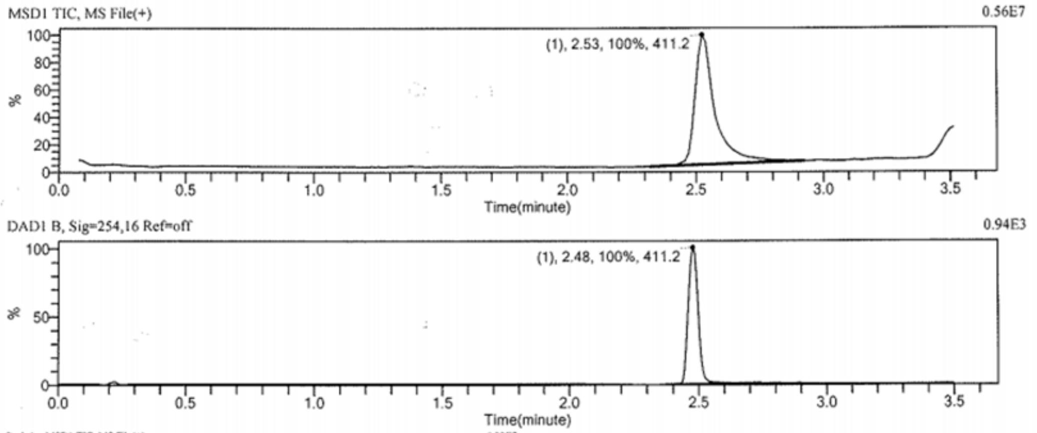

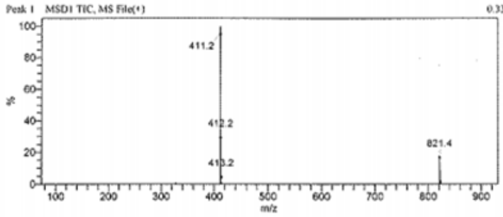

Compound 17

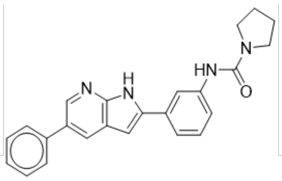
HN
N
H
N
O
N

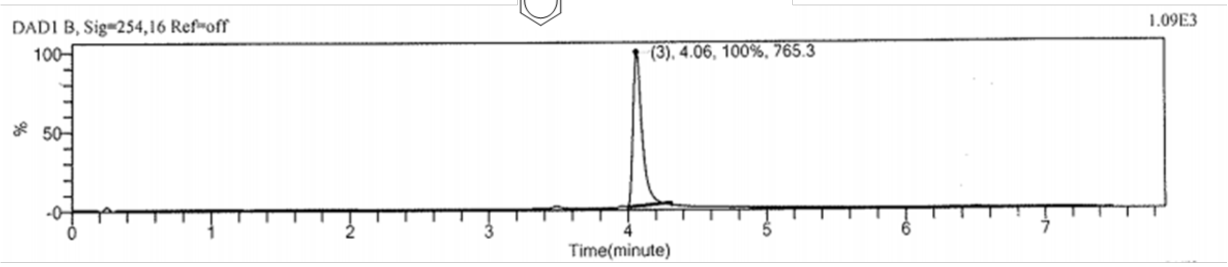
DAD1 B, Sig=254,16 Ref=off
1.09E3
(3), 4.06, 100%, 765.3
%
Time(minute)

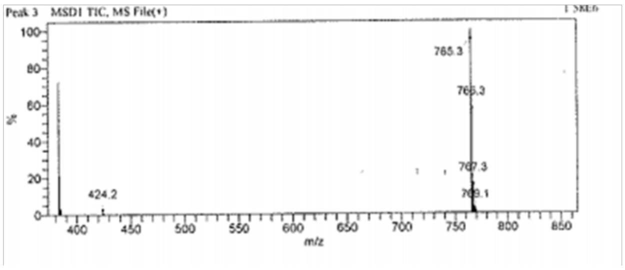
Peak 3 MSD1 TIC, MS File(+)
765.3
424.2
%
m/z

Compound 18

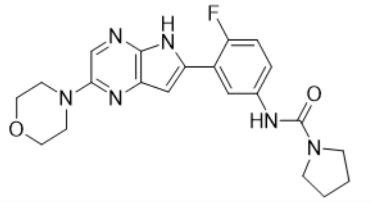

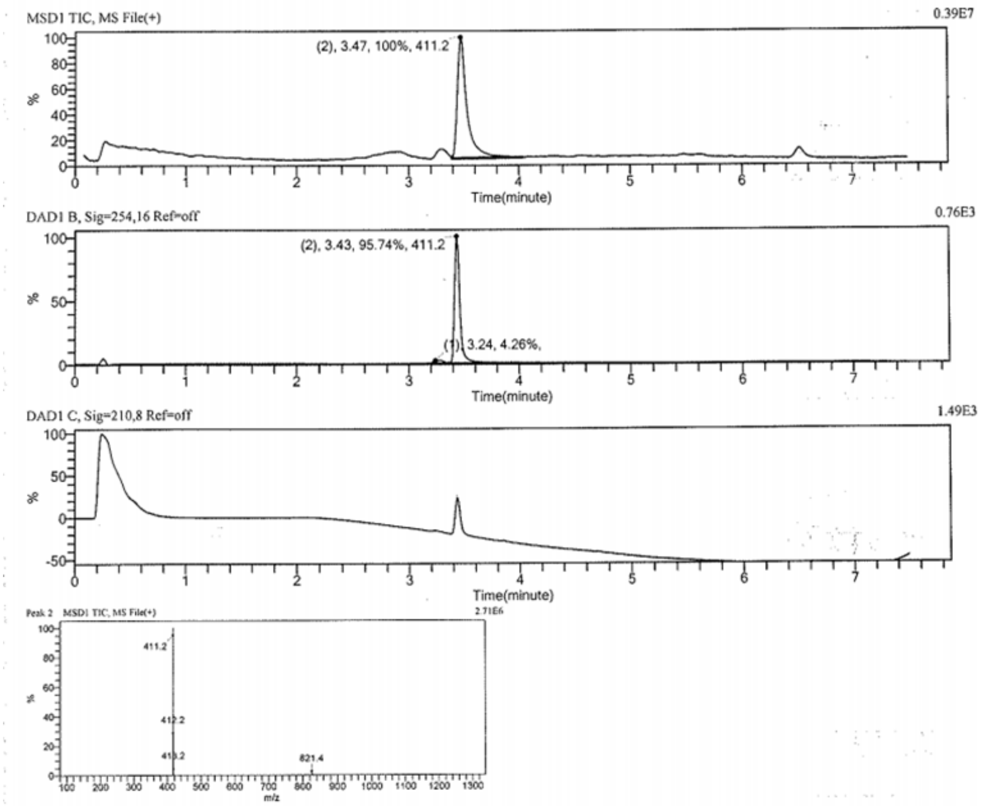
MSD1 TIC, MS File(+)
0.39E7
(2), 3.47, 100%, 411.2
Time(minute)
DAD1 B, Sig=254,16 Ref=off
0.76E3
(2), 3.43, 95.74%, 411.2
(1), 3.24, 4.26%,
Time(minute)
DAD1 C, Sig=210,8 Ref=off
1.49E3
Time(minute)
411.2
821.4
m/z

Compound 19

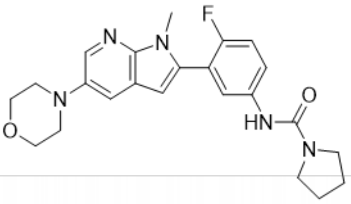

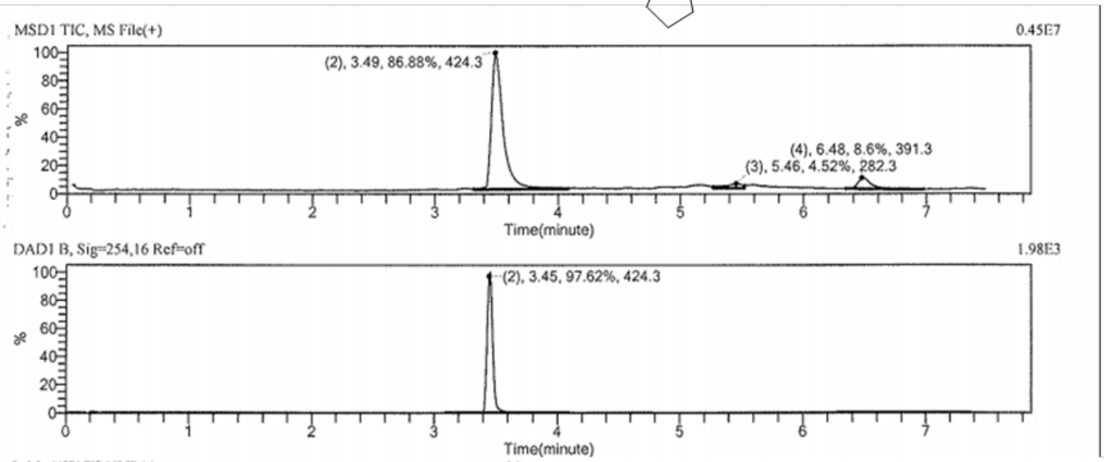

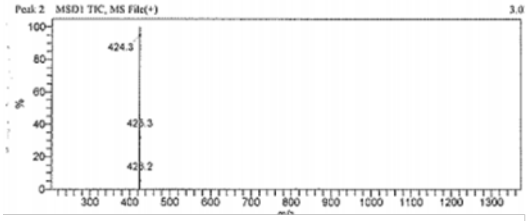

Compound 20

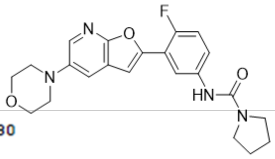

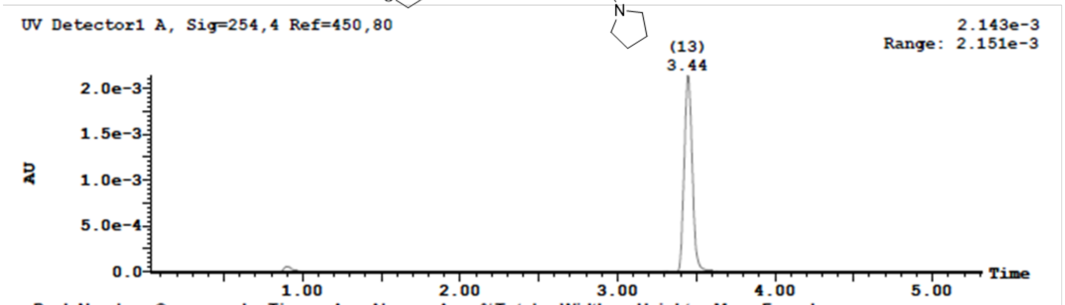

Peak Number	Compound	Time	AreaAbs	Area %Total	Width	Height	Mass Found
2		0.91	2e+002	2.62	1	5e+001	
13		3.45	8e+003	97.38	1	2e+003	

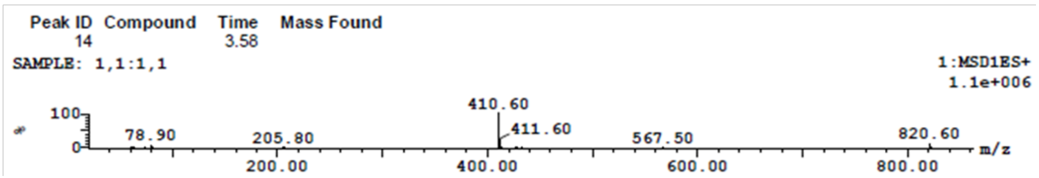

Compound 21

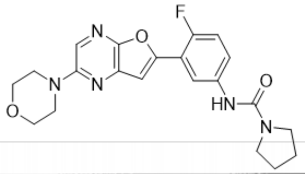

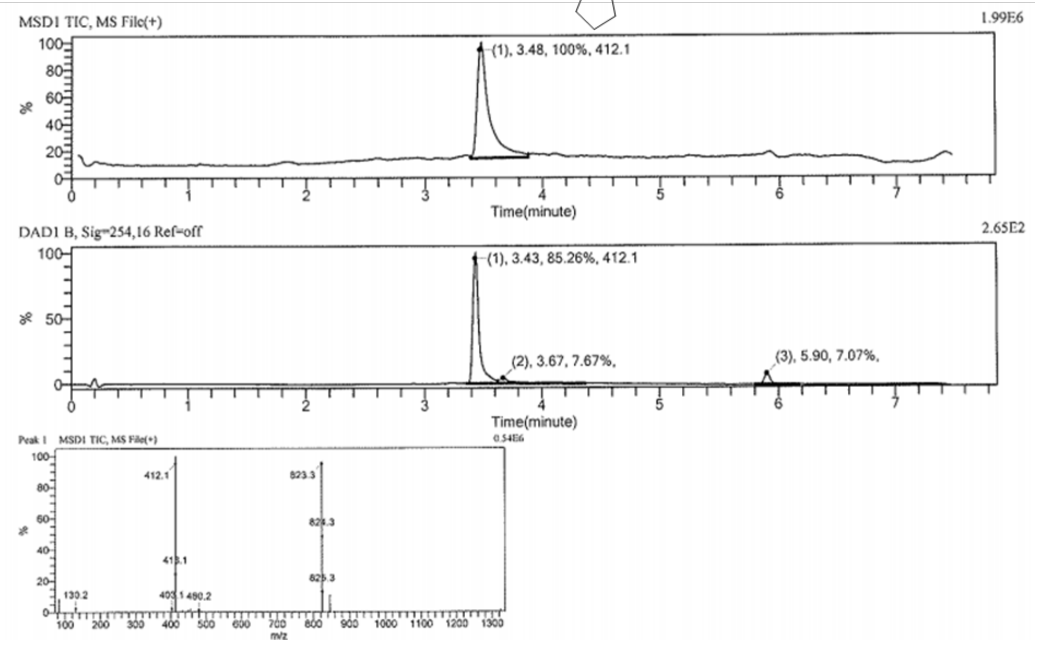

Compound 22

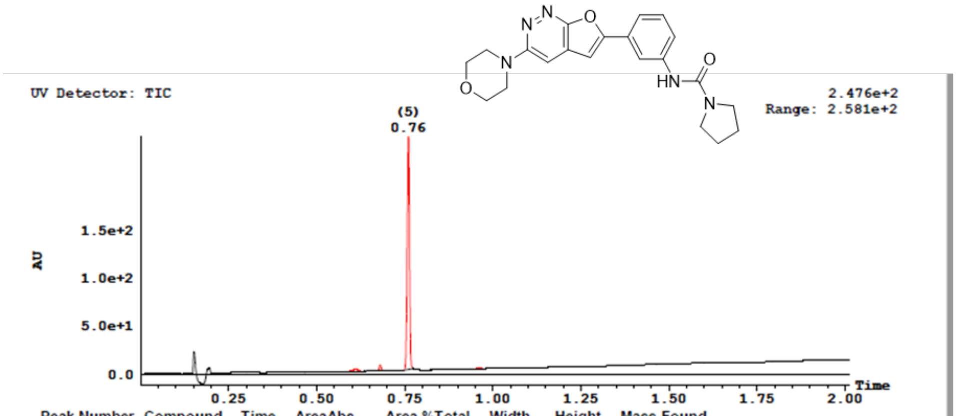

Peak Number	Compound	Time	AreaAbs	Area %Total	Width	Height	Mass Found
2		0.61	4e+004	1.94	0	2e+006	
4		0.68	4e+004	2.02	0	6e+006	
5		0.76	2e+006	95.47	0	2e+008	
7		0.96	1e+004	0.58	0	1e+006	

Peak ID	Compound	Time	Mass Found
5		0.76	

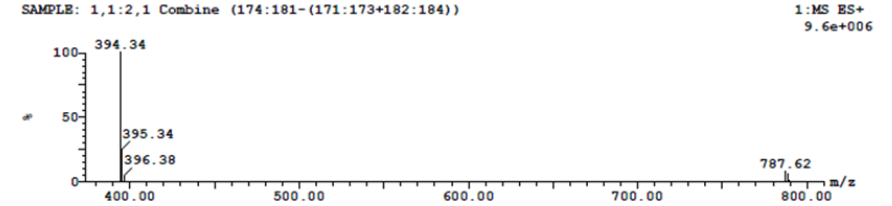

Compound 24

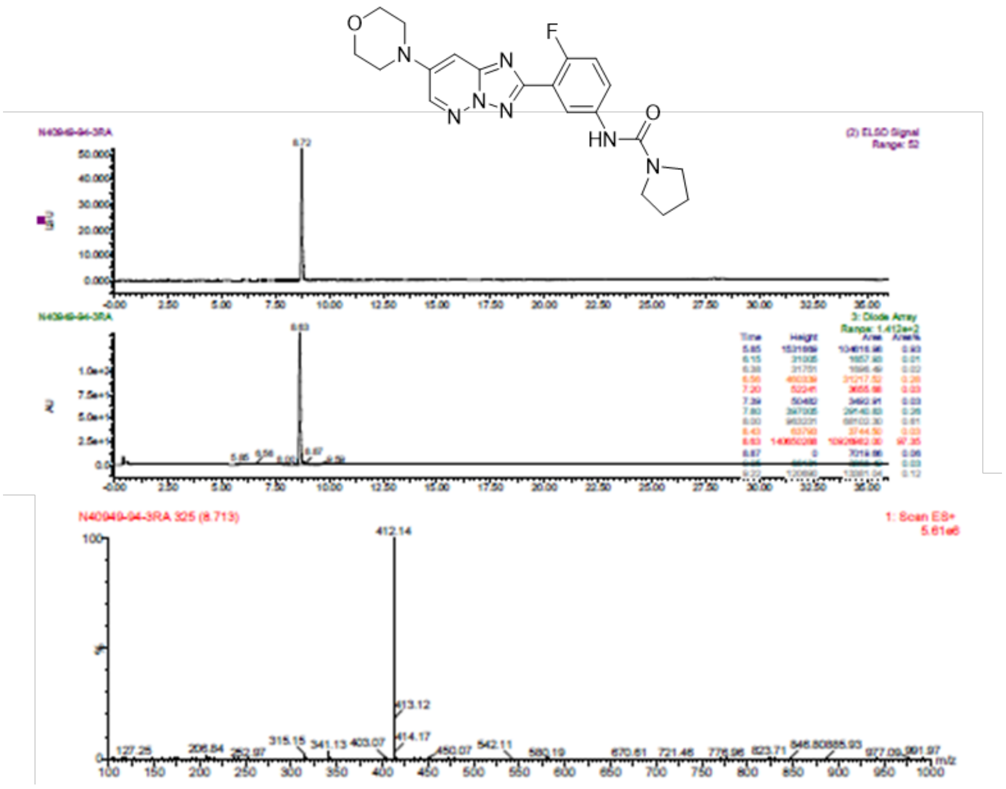

Compound 25

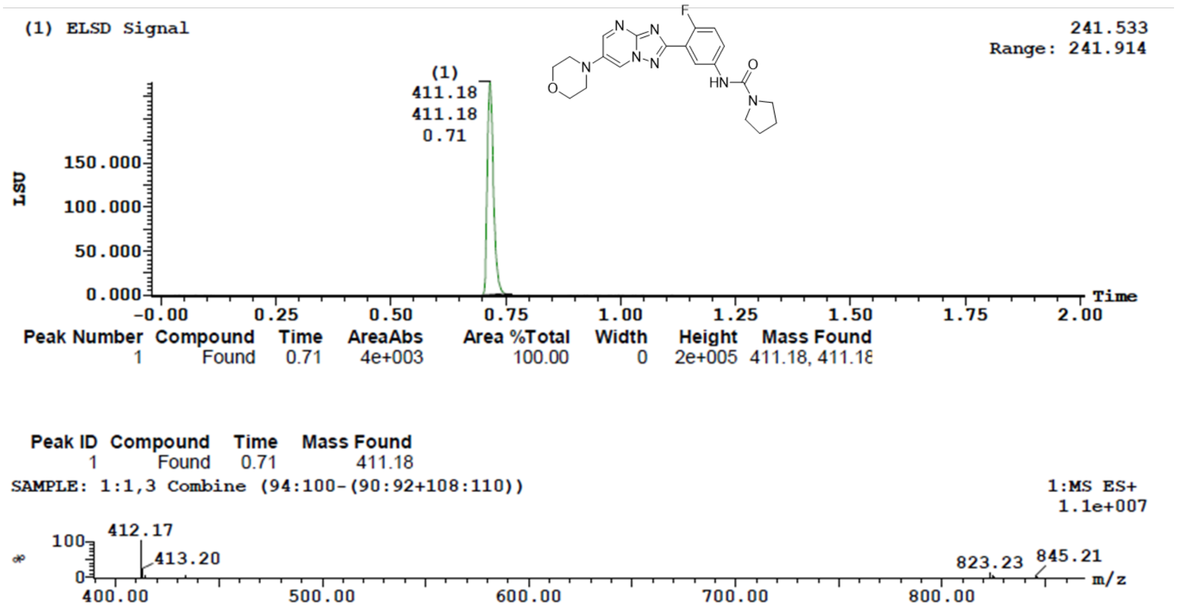

Compound 26

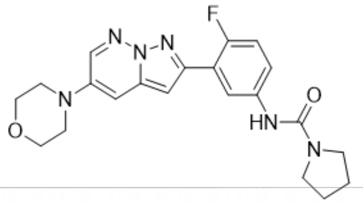

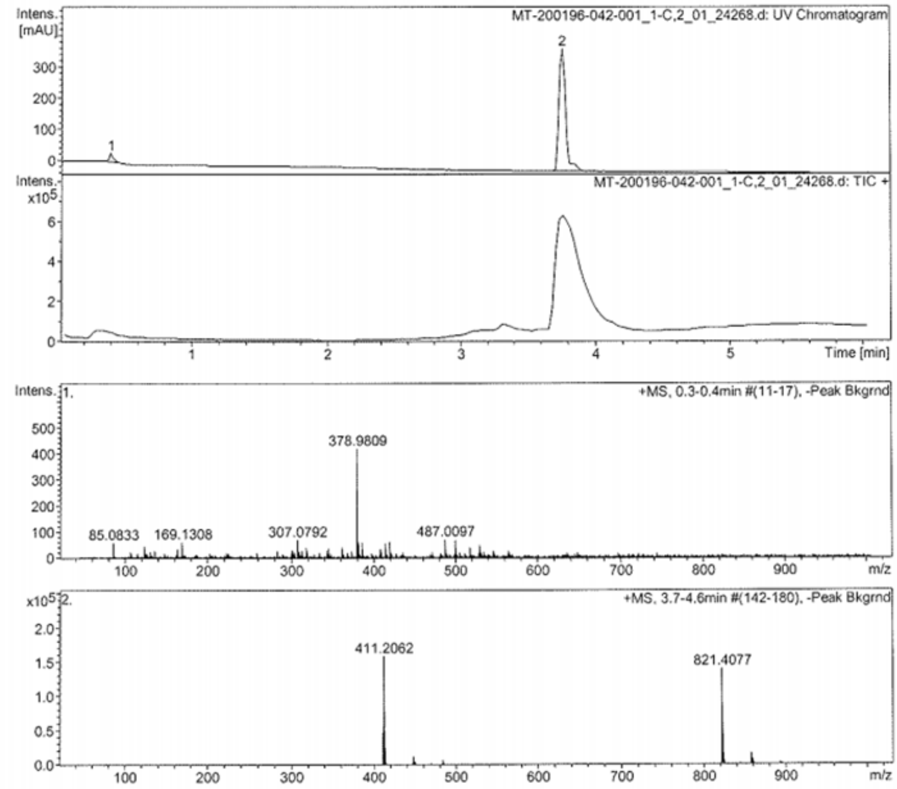

#	RT [min]	Area	Area Frac. %
1	0.4	64.518	4.5
2	3.8	1371.849	95.5

Compound 27

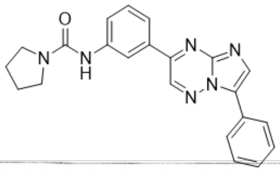

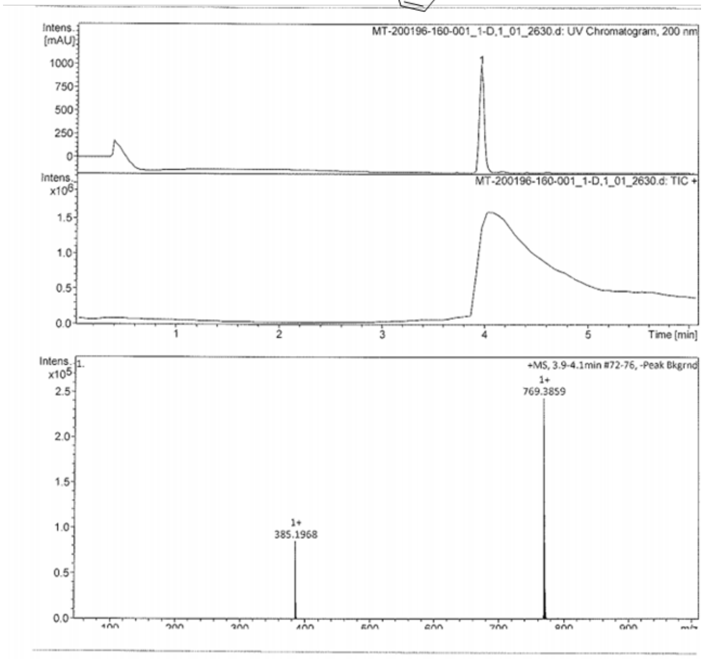

#	RT [min]	Area	Area Frac. %
1	4.0	3890.0	100.00

Compound 29

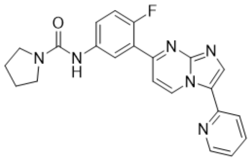

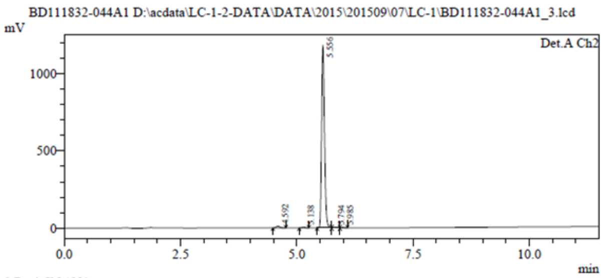

1 Det.A Ch2 / 254nm

<Result>

PeakTable

Detector A Ch2 254nm

Peak#	Ret. Time	Area	Height	Area %
1	4.592	60367	11097	1.153
2	5.138	21665	5130	0.414
3	5.556	5117367	1183243	97.724
4	5.794	23088	3644	0.441
5	5.985	14089	2886	0.269
Total		5236575	1205999	100.000

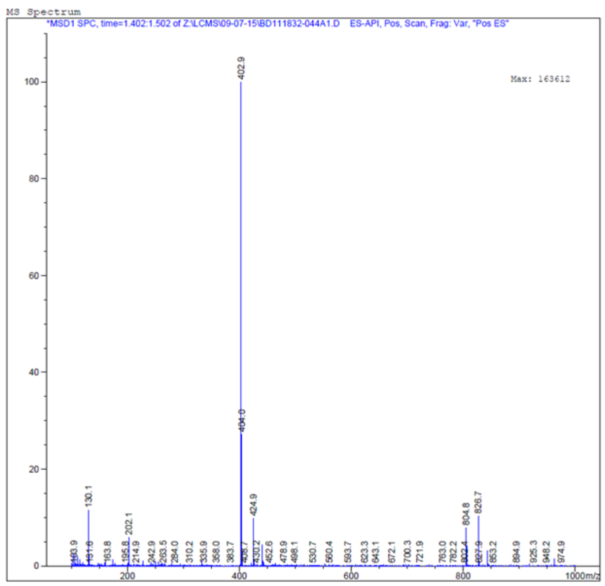

Compound 30

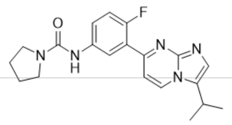

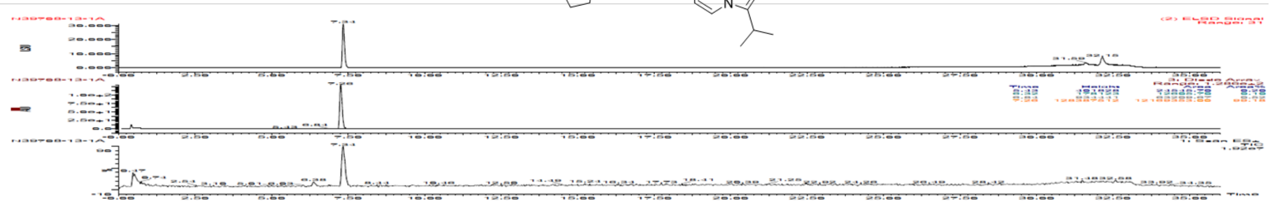

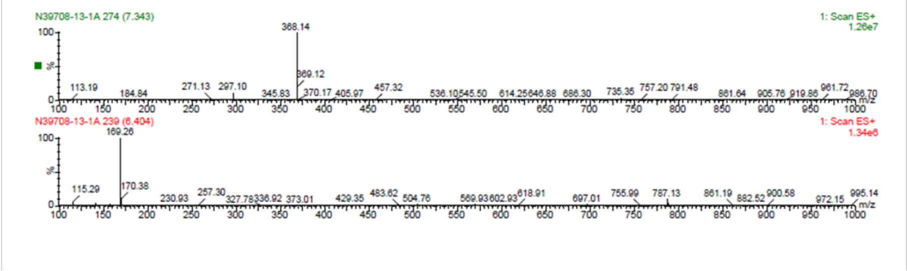

Compound 31

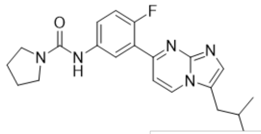

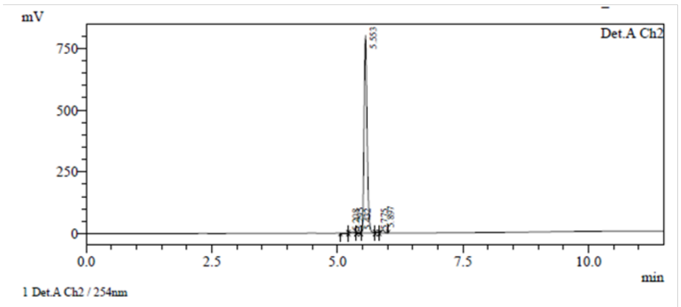

<Result>

PeakTable

Detector A Ch2 254nm

Peak#	Ret. Time	Area	Height	Area %
1	5.208	4931	864	0.143
2	5.295	25570	4530	0.742
3	5.432	14080	3207	0.409
4	5.553	3345994	799892	97.129
5	5.775	5464	1263	0.159
6	5.897	48852	12274	1.418
Total		3444892	822030	100.000

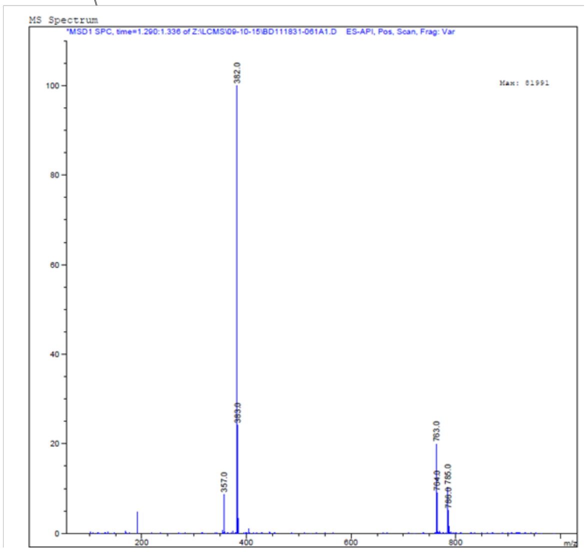

Compound 32

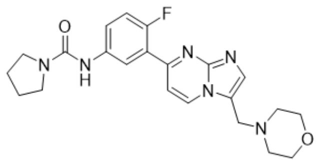

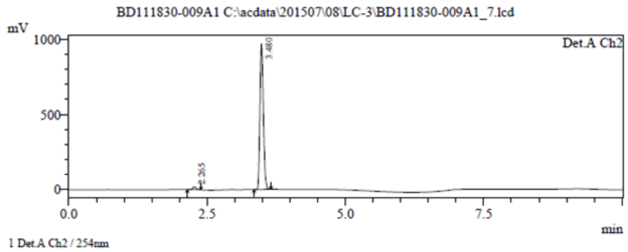

<Result>

PeakTable

Detector A Ch2 254nm

Peak#	Ret. Time	Area	Height	Area %
1	2.265	74807	19512	1.760
2	3.480	4175728	969491	98.240
Total		4250535	989003	100.000

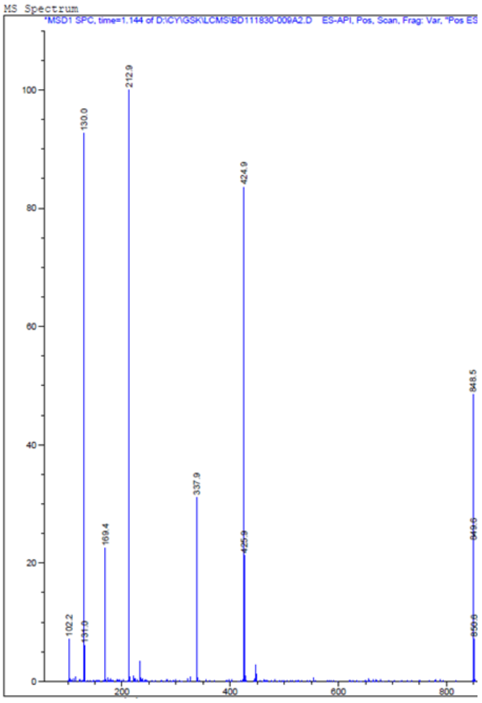

Compound 33

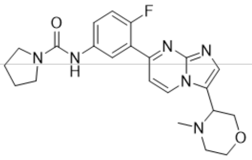

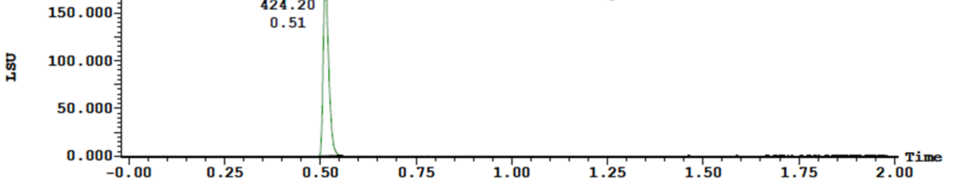

Peak Number	Compound	Time	AreaAbs	Area %Total	Width	Height	Mass Found
1	Found	0.51	3e+003	100.00	0	2e+005	424.20, 424.20

Peak ID	Compound	Time	Mass Found
1	Found	0.51	424.20

SAMPLE: 2:16,6 Combine (67:73-(39:41+102:104)) 1:MS ES+ 2.5e+007

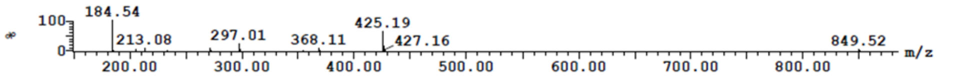

Compound 34

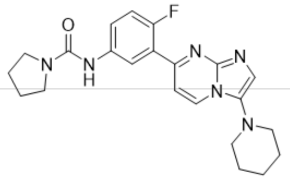

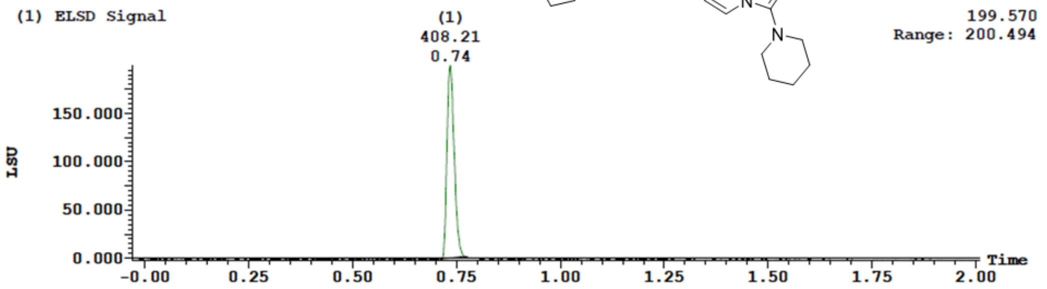

Peak Number	Compound	Time	AreaAbs	Area %Total	Width	Height	Mass Found
1	Found	0.74	4e+003	100.00	0	2e+005	408.21

Peak ID	Compound	Time	Mass Found
1	Found	0.73	408.21

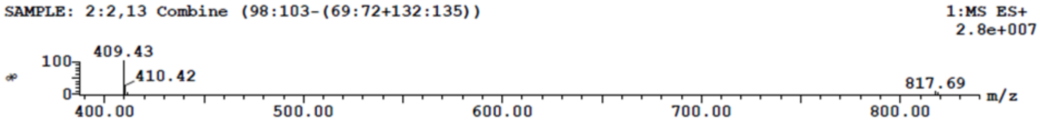

Compound 35

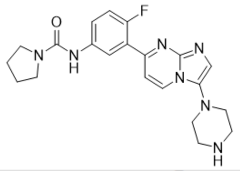

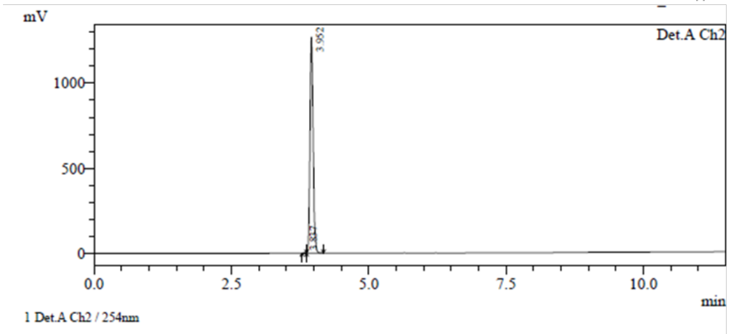

<Result>

PeakTable

Detector A Ch2 254nm

Peak#	Ret. Time	Area	Height	Area %
1	3.837	8768	2836	0.173
2	3.952	5068072	1268488	99.827
Total		5076840	1271324	100.000

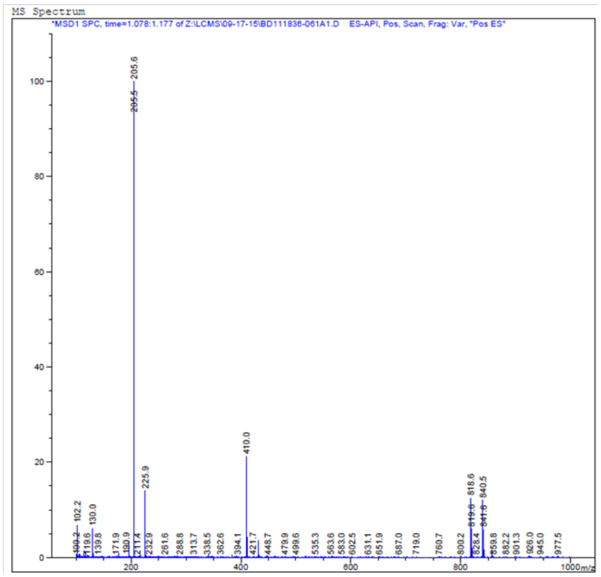

Compound 36

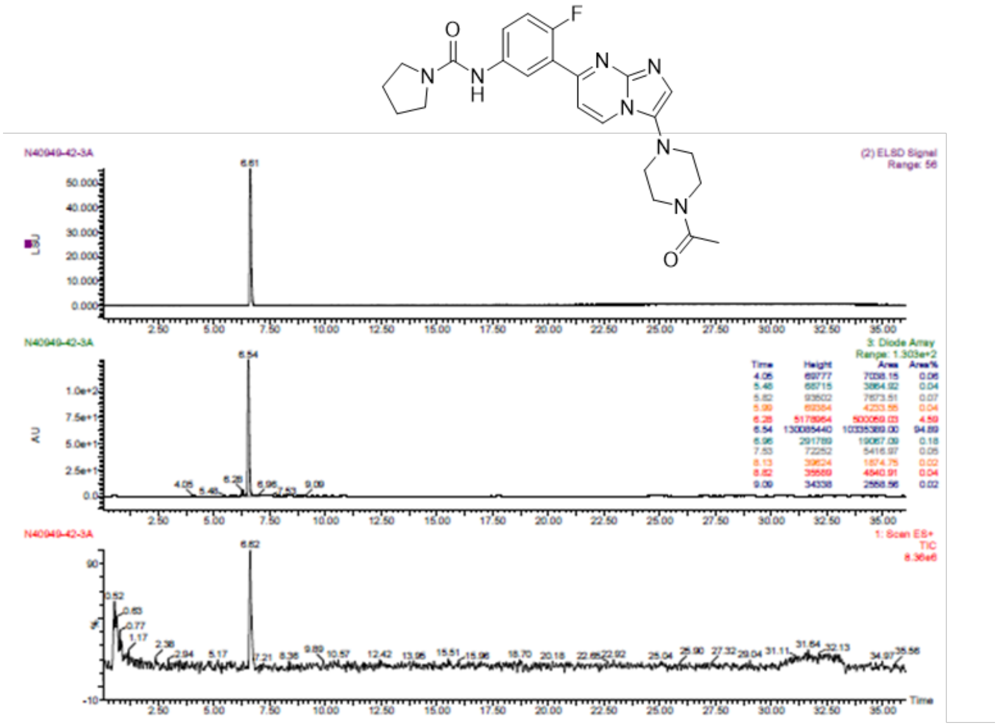

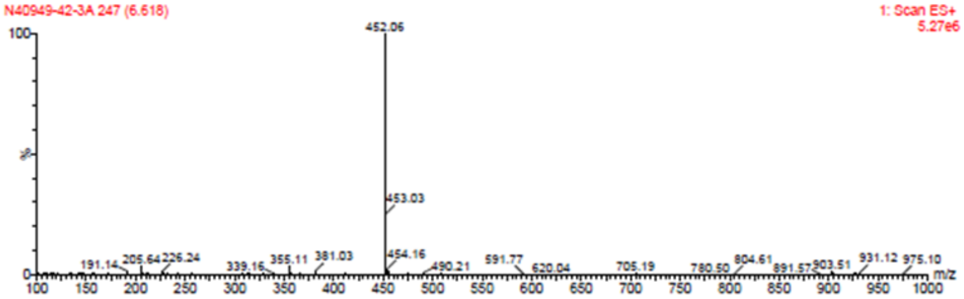

Compound 37

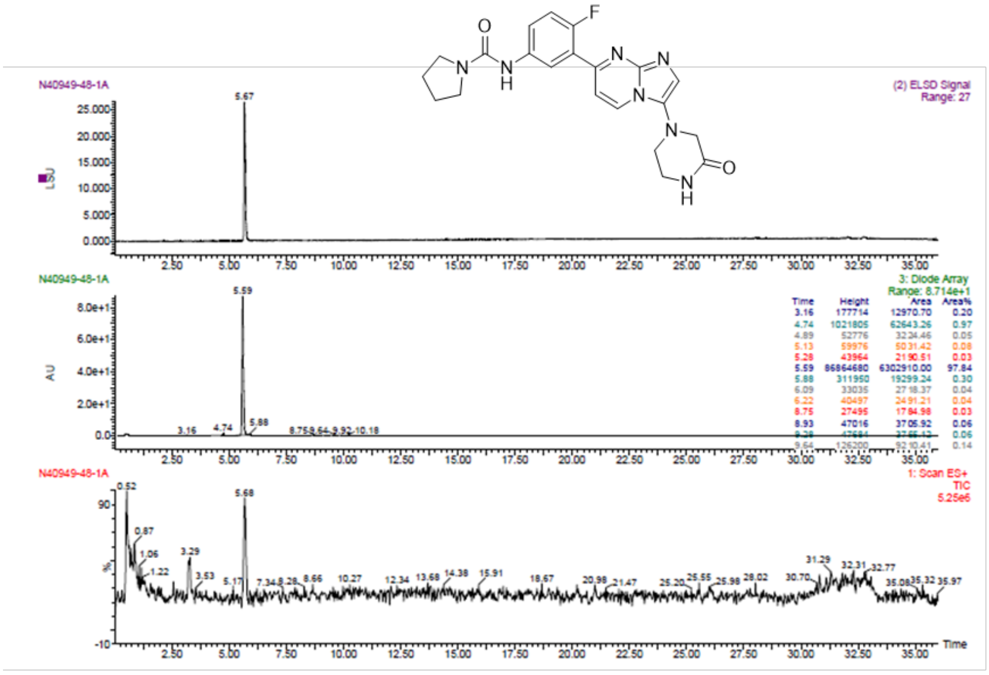
N40949-48-1A
(2) ELSD Signal
Range: 27
5.67
N40949-48-1A
3: Diode Array
Range: 8.714e+1
5.59
Time Height Area Area%
3.16 177714 12970.70 0.20
4.74 1021805 62643.26 0.97
4.89 52776 3224.46 0.05
5.13 59976 5031.42 0.08
5.28 43964 2190.51 0.03
5.59 86864680 6302910.00 97.84
5.88 311950 19299.24 0.30
6.09 33035 2718.37 0.04
6.22 40497 2491.21 0.04
8.75 27495 1784.98 0.03
8.93 47016 3705.92 0.06
9.64 126200 9210.41 0.14
N40949-48-1A
1: Scan ES+
TIC
5.25e6
5.68
Time

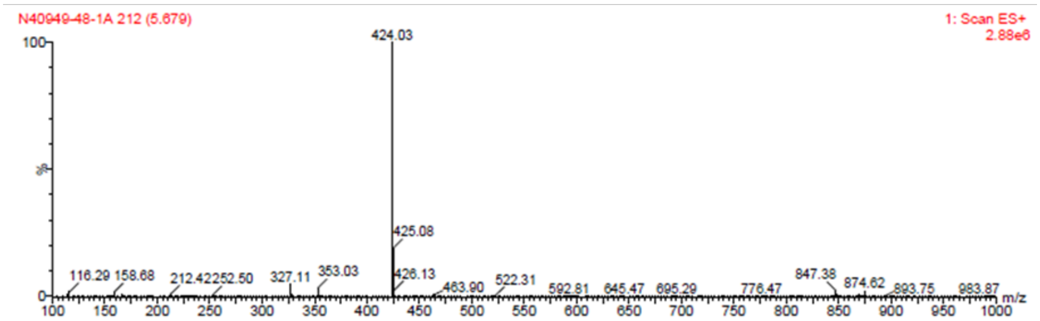
N40949-48-1A 212 (5.679)
1: Scan ES+
2.88e6
424.03
425.08
426.13
m/z

Compound 38

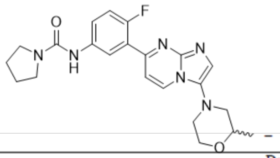

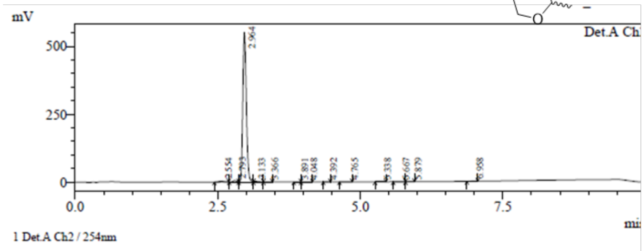

<Result>

PeakTable

Detector A Ch2 254nm

Peak#	Ret. Time	Area	Height	Area %
1	2.554	16837	3022	0.694
2	2.793	20699	4737	0.853
3	2.964	2353041	551217	97.025
4	3.133	11970	2153	0.494
5	3.366	1767	318	0.073
6	3.891	1379	307	0.057
7	4.048	1993	309	0.082
8	4.392	2171	576	0.090
9	4.765	2233	312	0.092
10	5.338	3670	659	0.151
11	5.667	3420	711	0.141
12	5.879	1809	444	0.075
13	6.958	4205	836	0.173

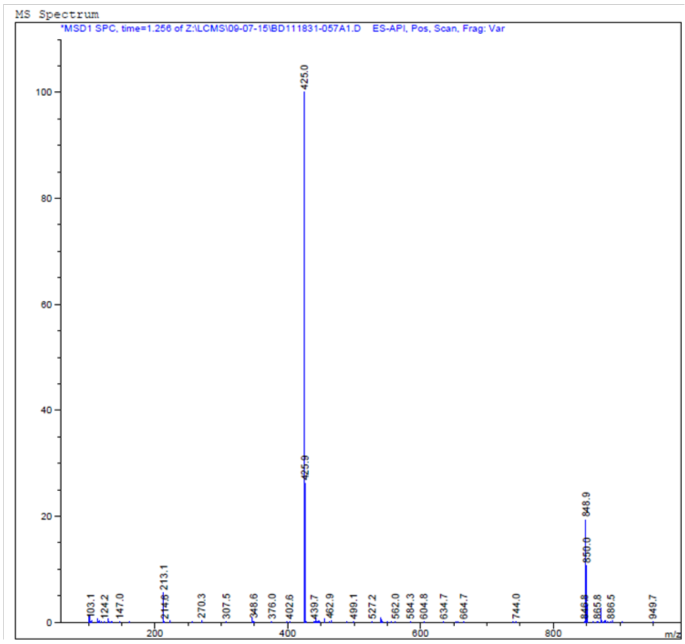

Compound 39

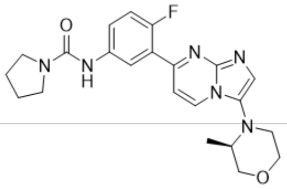

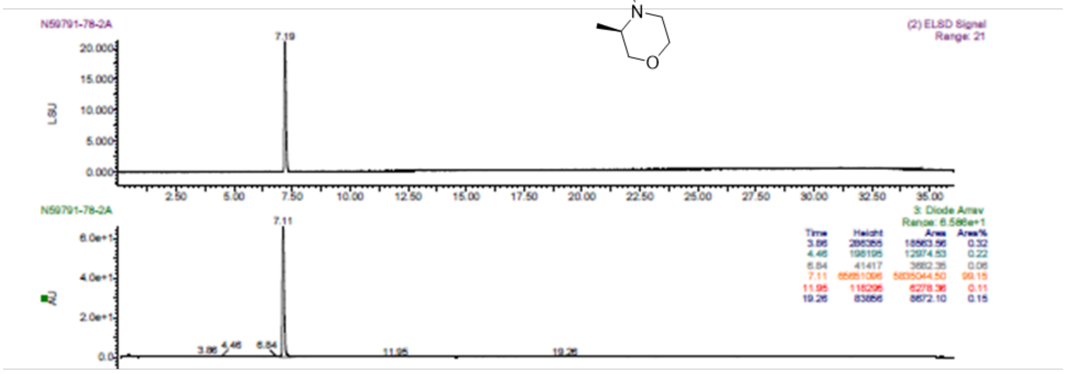

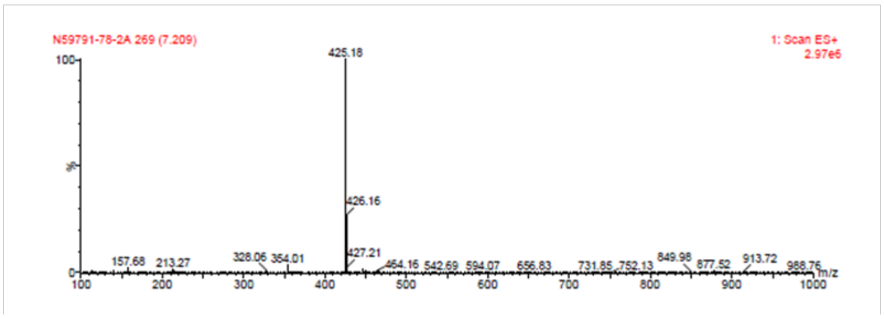

Compound 40

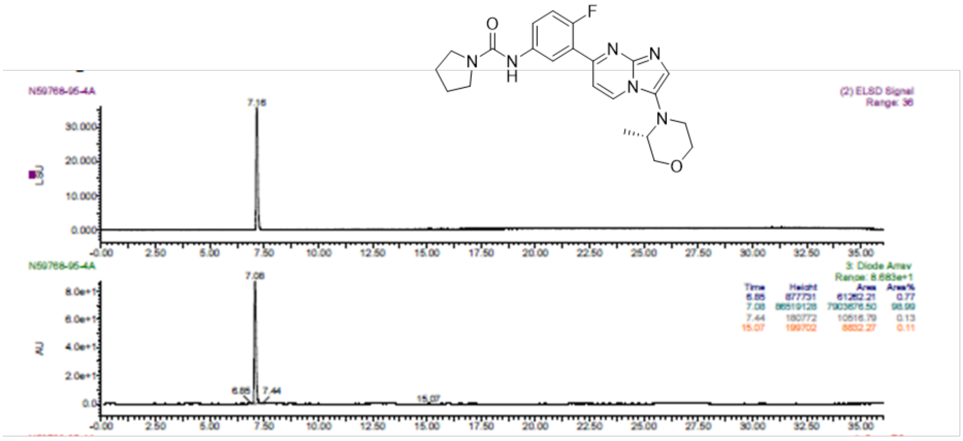

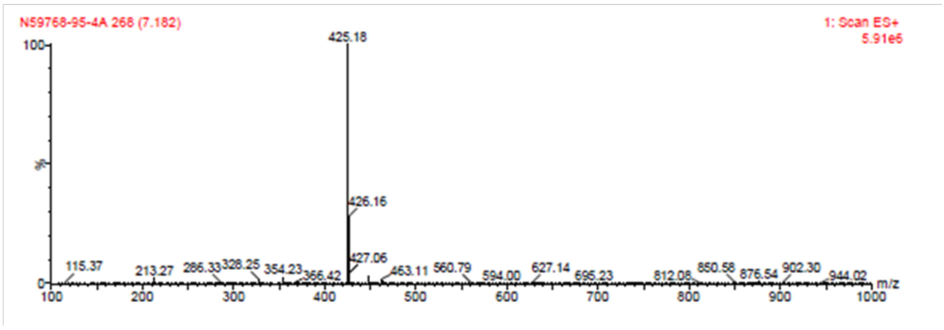

Compound 41

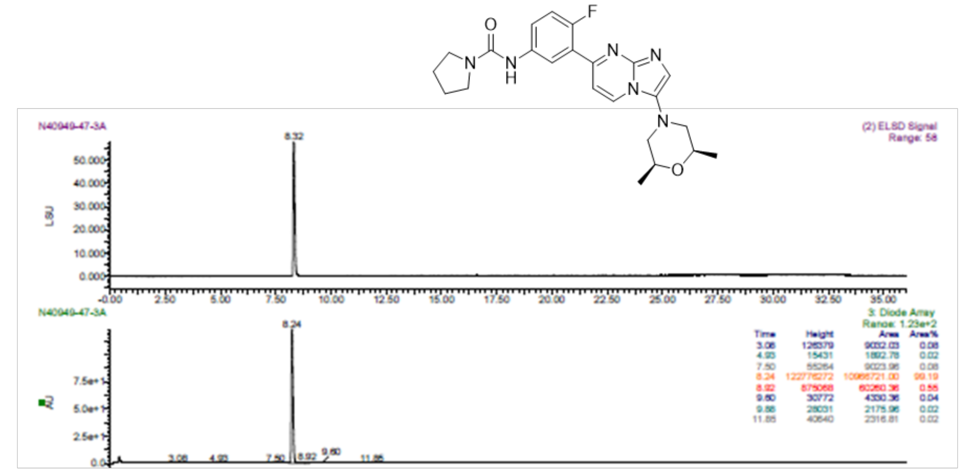

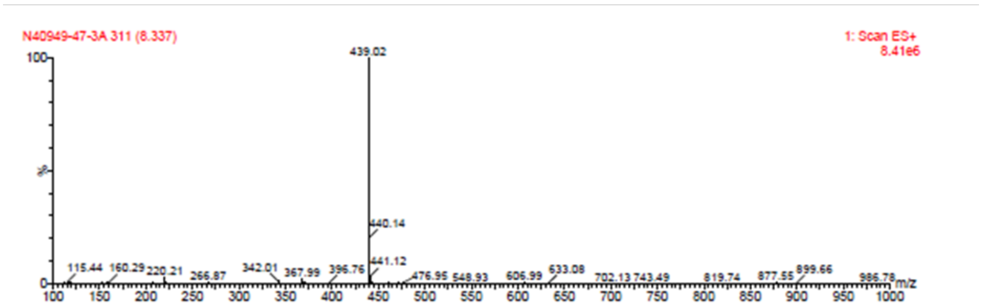

Compound 42

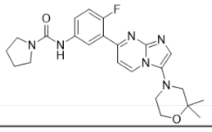

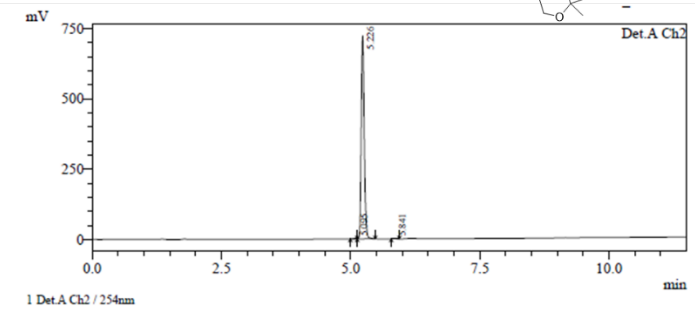

<Result>

PeakTable

Detector A Ch2 254nm

Peak#	Ret. Time	Area	Height	Area %
1	5.095	8285	1710	0.283
2	5.226	2920116	721911	99.587
3	5.841	3828	975	0.131
Total		2932228	724596	100.000

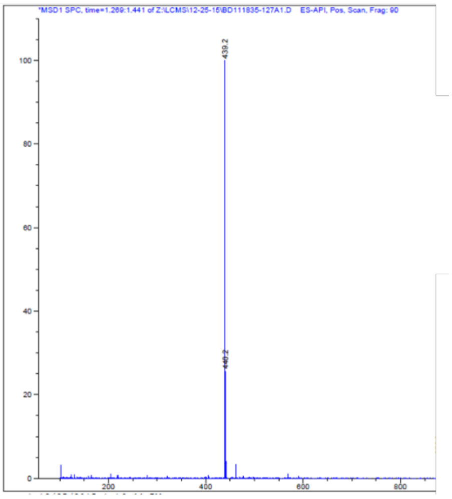

Compound 43

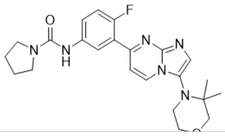

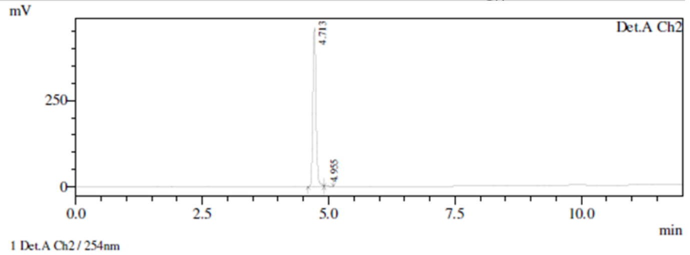

<Result>

PeakTable

Detector A Ch2 254nm

Peak#	Ret. Time	Area	Height	Area %
1	4.713	1977124	457113	99.175
2	4.955	16456	3574	0.825
Total		1993581	460687	100.000

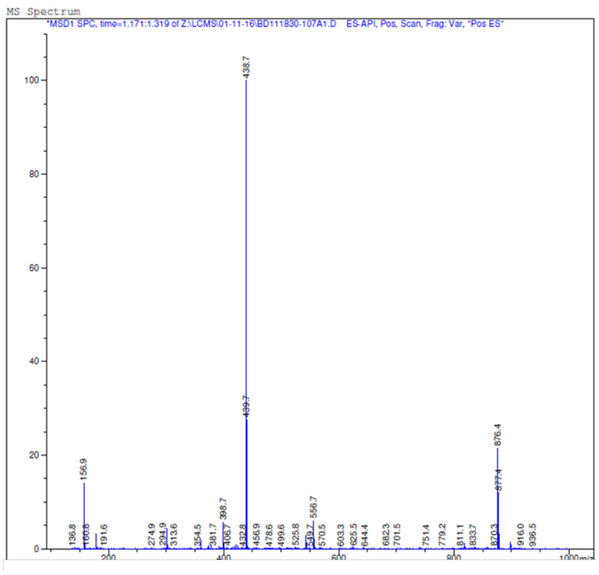

Compound 44

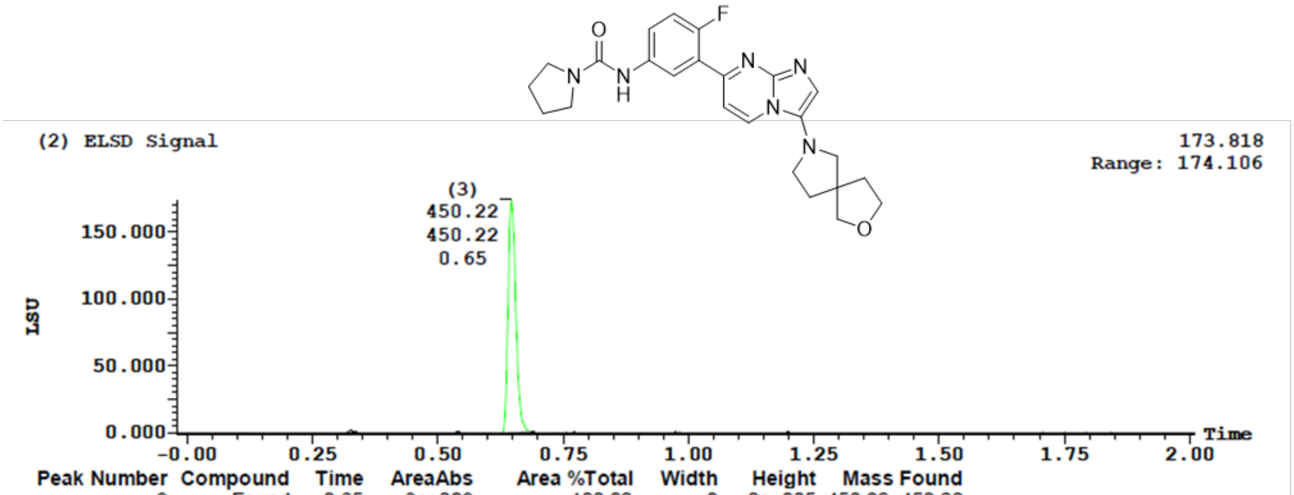

Peak Number	Compound	Time	AreaAbs	Area %Total	Width	Height	Mass Found
3	Found	0.65	3e+003	100.00	0	2e+005	450.22, 450.22

Peak ID	Compound	Time	Mass Found
3	Found	0.65	450.22

SAMPLE: 2:1,7 Combine (86:91-(56:59+122:124))

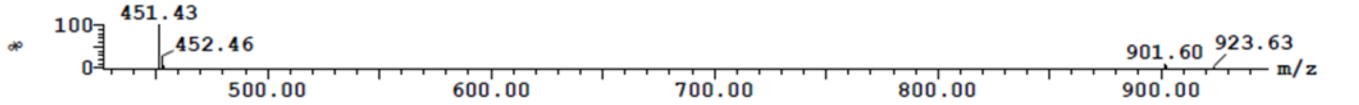

Compound 45

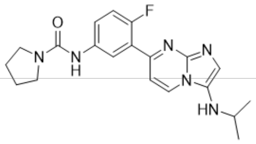

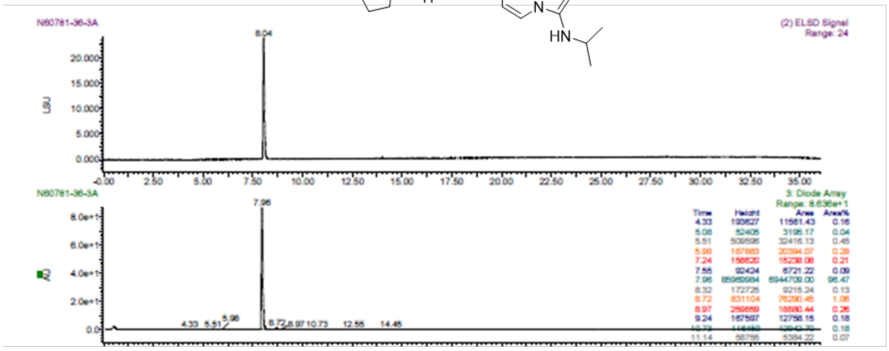

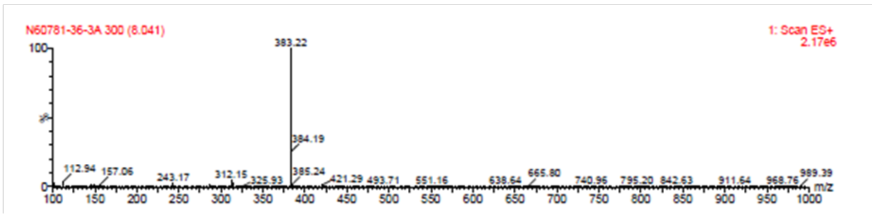

Compound 46

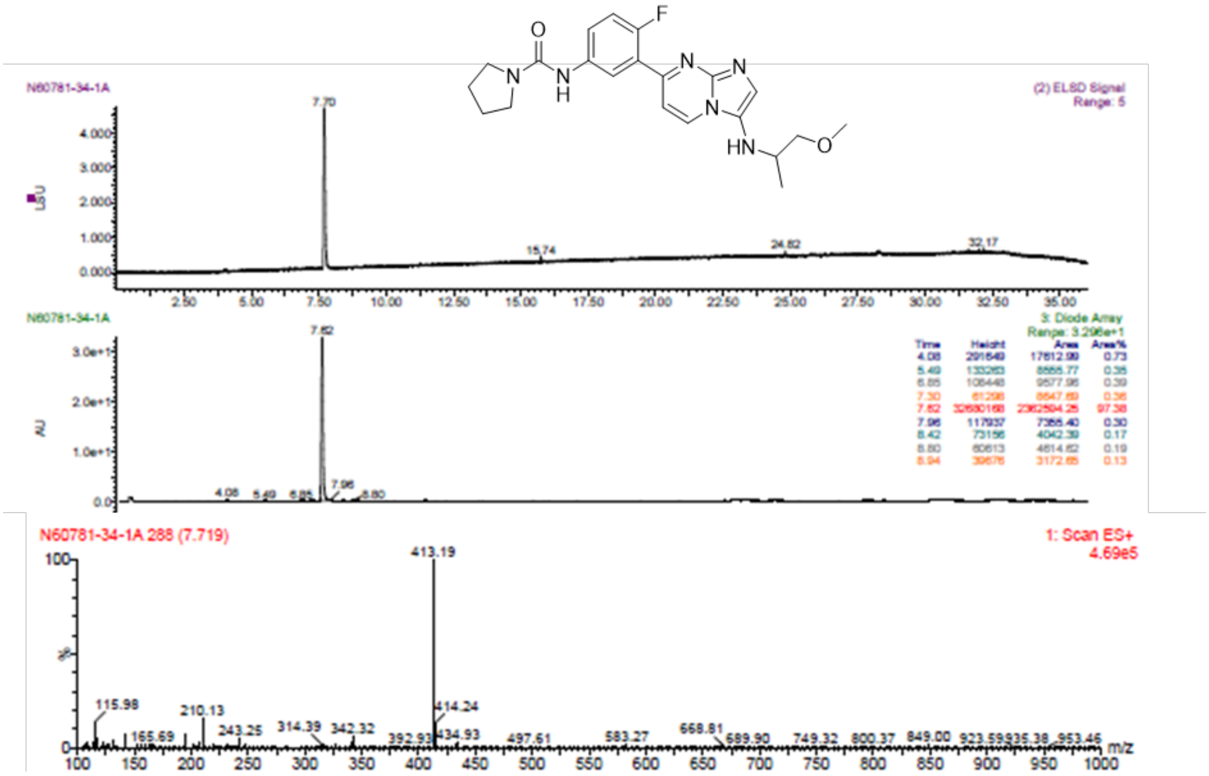
O
F
N
N
H
N
N
N
HN
O
N60781-34-1A
(2) ELSD Signal
Range: 5
LSU
7.70
15.74
24.82
32.17
2.50 5.00 7.50 10.00 12.50 15.00 17.50 20.00 22.50 25.00 27.50 30.00 32.50 35.00
N60781-34-1A
3: Diode Array
Range: 3.296e+1
AU
7.82
4.08 5.49 6.85 7.96 8.80
Time Height Area Area%
4.08 291649 17612.99 0.73
5.49 133263 8565.77 0.35
6.85 108448 9577.96 0.39
7.30 61298 8647.69 0.36
7.82 32680168 2362594.25 97.38
7.96 117937 7365.40 0.30
8.42 73156 4042.39 0.17
8.80 60613 4614.62 0.19
8.94 39676 3172.65 0.13
N60781-34-1A 288 (7.719)
1: Scan ES+
4.69e5
413.19
115.98
210.13
414.24
165.69
243.25
314.39
342.32
392.93
434.93
497.61
583.27
668.81
689.90
749.32
800.37
849.00
923.59
935.38
953.46
m/z
100 150 200 250 300 350 400 450 500 550 600 650 700 750 800 850 900 950 1000

References

1 Bode, C.; Boezio, A.; Cheng, A. C.; Choquette, D.; Coats, J. R.; Copeland, K. W.; Huang, H.; La, D.; Lewis, R.; Liao, H.; Potashman, M.; Stellwagen, J.; Yl, S.; Norman, M.; Stec, M.; Peterson, E. A.; Gracefa, R. Heteroaryl compounds as PIKK inhibitors. WO 2010/132598 (2010).

2 Brand, S.; Dodd, P. G.; Ko, E.-J.; Marco Martin, M.; Miles, T. J.; Sandberg, L. H.; Thomas, M. G.; Thompson, S. Compounds. WO 2017025416 (2017).

3 Kim, J.; Lee, D.; Park, C.; So, W.; Jo, M.; Ok, T.; Kwon, J.; Kong, S.; Jo, S.; Kim, Y.; Choi, J.; Kim, H. C.; Ko, Y.; Choi, I.; Park, Y.; Yoon, J.; Ju, M. K.; Kim, J.; Han, S. J.; Kim, T. H.; Cechetto, J.; Nam, J.; Sommer, P.; Liuzzi, M.; Lee, J.; No, Z. Discovery of Phenylaminopyridine Derivatives as Novel HIV-1 Non-nucleoside Reverse Transcriptase Inhibitors. *ACS Med Chem Lett* **2012**, *3*, 678-682.

4 De Rycker, M.; Hallyburton, I.; Thomas, J.; Campbell, L.; Wyllie, S.; Joshi, D.; Cameron, S.; Gilbert, I. H.; Wyatt, P. G.; Frearson, J. A.; Fairlamb, A. H.; Gray, D. W. Comparison of a high-throughput high-content intracellular *Leishmania donovani* assay with an axenic amastigote assay. *Antimicrob. Agents Chemother.* **2013**, *57*, 2913-2922.

5 Brand, S.; Ko, E. J.; Viayna, E.; Thompson, S.; Spinks, D.; Thomas, M.; Sandberg, L.; Francisco, A. F.; Jayawardhana, S.; Smith, V. C.; Jansen, C.; De Rycker, M.; Thomas, J.; MacLean, L.; Osuna-Cabello, M.; Riley, J.; Scullion, P.; Stojanovski, L.; Simeons, F. R. C.; Epemolu, O.; Shishikura, Y.; Crouch, S. D.; Bakshi, T. S.; Nixon, C. J.; Reid, I. H.; Hill, A. P.; Underwood, T. Z.; Hindley, S. J.; Robinson, S. A.; Kelly, J. M.; Fiandor, J. M.; Wyatt, P. G.; Marco, M.; Miles, T. J.; Read, K. D.; Gilbert, I. H. Discovery and Optimization of 5-Amino-1,2,3-triazole-4-carboxamide Series against *Trypanosoma cruzi*. *J Med Chem* **2017**, *60*, 7284-7299.

6 Hill, A. P.; Young, R. J. Getting physical in drug discovery: a contemporary perspective on solubility and hydrophobicity. *Drug Discov Today* **2010**, *15*, 648-655.

7 Thomas, M. G., De Rycker, M., Ajakane, M., Albrecht, S., Álvarez-Pedraglio, A. I., Boesche, M., Brand, S., Campbell, L., Cantizani-Perez, J., Cleghorn, L. A. T., Copley, R. C. B., Crouch, S. D., Daugan, A., Drewes, G., Ferrer, S., Ghidelli-Disse, S., Gonzalez, S., Gresham, S. L., Hill, A. P., Hindley, S. J., Lowe, R. M., MacKenzie, C. J., MacLean, L., Manthri, S., Martin, F., Miguel-Siles, J., Nguyen, V. L., Norval, S., Osuna-Cabello, M., Woodland, A., Patterson, S., Pena, I., Quesada-Campos, M. T., Reid, I. H., Revill, C., Riley, J., Ruiz-Gomez, J. R., Shishikura, Y., Simeons, F. R. C., Smith, A., Smith, V. C., Spinks, D., Stojanovski, L., Thomas, J., Thompson, S., Underwood, T., Gray, D. W., Fiandor, J. M., Gilbert, I. H., Wyatt, P. G., Read, K. D.Miles, T. J. Identification of GSK3186899/DDD853651 as a Preclinical Development Candidate for the Treatment of Visceral Leishmaniasis. *J Med Chem* **2019**, *62*, 1180-1202.

8 Ryde, U.; Söderhjelm, P. Ligand-Binding Affinity Estimates Supported by Quantum-Mechanical Methods. *Chem Rev* **2016**, *116*, 5520-5566.

9 Lukac, I.; Abdelhakim, H.; Ward, R. A.; St-Gallay, S. A.; Madden, J. C.; Leach, A. G. Predicting protein-ligand binding affinity and correcting crystal structures with quantum mechanical calculations: lactate dehydrogenase A. *Chem Sci* **2019**, *10*, 2218-2227.

10 Mazanetz, M. P.; Chudyk, E.; Fedorov, D. G.; Alexeev, Y. in *Computer-Aided Drug Discovery* (ed W Zhang) 217-255 (Springer, 2015).

11 Suenaga, M. Facio: New Computational Chemistry Environment for PC GAMESS. *J. Comput. Chem. Jpn* **2005**, *4*, 25-32.

12 Suenaga, M. Development of gui for gamess/fmo calculation. *J. Comput. Chem. Jpn* **2008**, *7*, 33-54.

13 Schmidt, M. W.; Baldridge, K. K.; Boatz, J. A.; Elbert, S. T.; Gordon, M. S.; Jensen, J. H.; Koseki, S.; Matsunaga, N.; Nguyen, K. A.; Su, S. J.; Windus, T. L.; Dupuis, M.; Montgomery, J. A. General atomic and molecular electronic-structure system. *J. Comput. Chem.* **1993**, *14*, 1347-1363.

14 Gordon, M. S.; Schmidt, M. W. *Advances in electronic structure theory: GAMESS a decade later*. (Elsevier Science Bv, 2005).

15 Van Rossum, G.; Drake, F. L. Python 3 Reference Manual: CreateSpace: Scotts Valley, CA, 2009

16 Fedorov, D. G. The fragment molecular orbital method: theoretical development, implementation in GAMESS, and applications. *Wiley Interdiscip. Rev.-Comput. Mol. Sci.* **2017**, *7*, e1322.

17 Baker, N. A.; Sept, D.; Joseph, S.; Holst, M. J.; McCammon, J. A. Electrostatics of nanosystems: Application to microtubules and the ribosome. *Proc. Natl. Acad. Sci. U. S. A.* **2001**, *98*, 10037-10041.
